# Evaluation of Sensorial Markers in Deep-Fried Extra Virgin Olive Oils: First Report on the Role of Hydroxytyrosol and Its Derivatives

**DOI:** 10.3390/foods13233953

**Published:** 2024-12-07

**Authors:** Taha Mehany, José M. González-Sáiz, Jorge Martínez, Consuelo Pizarro

**Affiliations:** Department of Chemistry, University of La Rioja, 26006 Logroño, Spain; taha.abdellatif@unirioja.es (T.M.); josemaria.gonzalez@unirioja.es (J.M.G.-S.); jorge.martinez@unirioja.es (J.M.)

**Keywords:** EVOO, PCA, process analysis, deep-frying, experimental design, hydroxytyrosol, natural extract, sensory analysis, antioxidants

## Abstract

Extra virgin olive oil (EVOO) is one of the main daily food items consumed around the world, particularly in the Mediterranean region, and it has unique organoleptic properties. This study aims to determine the best frying conditions of EVOO supplemented with natural exogenous antioxidants enriched in hydroxytyrosol (HTyr) and its derivatives from olive fruit extract (OFE) to conserve its positive sensorial attributes while minimizing its sensorial defects, in particular, rancidity under high thermal processes. In this study, an experienced panel assessed the sensory attributes of nine EVOO varieties, olive oil 0.4° (mixed virgin olive oil (VOO) with refined olive oil (ROO)), Orujo olive oil, and olive oil 1° (EVOO mixed with ROO), compared with two sunflower oil types, performed using a deep-frying (D-F) process with numerous variables, i.e., frying time, frying temperature, and the addition of polyphenols enriched with HTyr. Results showed that most EVOO samples were stable under D-F at 170 °C for 3 h, with added polyphenols (∼650 mg/kg). Moreover, at these best values, the results stated that Arbequina, Picual, Royuella, Hojiblanca, Arbosana, and Manzanilla oils have low rancidity scores with values of 0, 1.7, 1.8, 2.3, 3.1, and 3.7, respectively, and stable/higher positive sensorial attributes, i.e., fruity, bitter, and pungent properties; however, olive oil 1° and olive oil 0.4° have high rancidity and low positive sensorial attributes. Notably, OFE helps maintain bitterness close to control in Picual, Koroneiki, Empeltre, and Arbosana oils. Furthermore, amongst the 19 tested sensory descriptors, only 8 descriptors—namely, fusty/muddy sediment, winey/vinegary/acid/sour, frostbitten olives (wet wood), rancid, fruity (green), fruity (ripe), bitter, and pungent—were successfully developed to allow characterization of the sensory quality of various olive oil categories under D-F. The present research confirmed that OFE may be used to provide stable EVOOs with higher positive sensorial qualities and lower defects and could be used as a natural antioxidant and promising strategy during the D-F process with EVOOs, not only for domestic practices but also at the industrial level.

## 1. Introduction

The Mediterranean diet (MD) plays a prominent fundamental role in preventing several important diseases, i.e., cancer and cardiovascular diseases. One of the main consumed food items in the MD is extra virgin olive oil (EVOO) for its health benefits, its phenolic compound content, and other minor bioactive substances that exert antimicrobial, antioxidant, anti-inflammatory, antitumor, and antidiabetic type-2 activities, and being utilized as salad dressing and for cooking and frying [1,2,3]. Additionally, EVOO can decrease systemic proinflammatory markers and fasting blood glucose and has a beneficial effect on the low-density lipoprotein (LDL) resistance to oxidative tension, decreases glycemia and dyslipidemia, and enhances anthropometric indicators [4]. The chemical composition of EVOO contains over 200 compounds. The health benefits of EVOO comprise its characteristic composition that can be divided into two main areas: (A) saponifiable division or major compounds (around 98%) containing glycerides (mono, di, and tri), phospholipids, and sterol esters, with monounsaturated fatty acids (MUFAs), particularly oleic acid, being the predominant fatty acid in EVOO (between 55 and 83%); and (B) unsaponifiable fraction or minor content (about 1–2%) consisting of several chemical constituents, including polyphenols, tocopherols, phytosterols, carotenoids, chlorophyll, volatile compounds, terpenes, squalene, and hydrocarbons, among others. These bioactive compounds are partly or entirely removed when olive oil is subjected to thermal, physical, and/or chemical refining treatments, such as refined olive oil (ROO) or lampante olive oil (LOO). For this reason, the minor fraction is mostly present in virgin olive oil (VOO) and, mainly, in EVOO [5,6]. Furthermore, the health claims regarding EVOO are mainly attributed to three areas: (a) oleic acid, which is found in abundance in olive oil and maintains normal blood cholesterol levels, (b) vitamin E and its contribution to the protection of cells from oxidative stress (this health claim can be used for food that is a source of vitamin E (at least 15% of 12 mg, which is the daily reference intake value for vitamin E, supplied by 100 g of the product)), and (c) polyphenols and their contribution to protecting blood lipids from oxidative stress (this claim presupposes that the consumption of 20 g of olive oil provides at least 5 mg of hydroxytyrosol (HTyr) and its derivatives (e.g., oleuropein derivatives and tyrosol (Tyr)) [7,8,9,10]. Several polyphenols in olive oils are hydrolysis products of oleuropein and ligstroside, and at least 30 different compounds have been identified so far. These compounds affect olive oil taste in terms of its bitterness and pungency, serve as antioxidant candidates, and contribute to several health values [11].

European legislation established that the VOO commercial classification is based on a series of chemical indicators and sensory attributes that are assessed through a panel test (PT) of selected descriptive sensorial attributes. According to this classification, EVOO is the premium class of VOO. VOO is classified as EVOO when the chemical indicators fall to the established limits, as well as the median of sensory defects = 0 and the median fruity property being > 0 [12,13]. The above legal classification does not reflect the nutraceutical aspects and the sensory attributes that make EVOO one of the healthful edible oils globally. The nutraceutical profile of EVOO is related to antioxidant compounds, e.g., tocopherols and hydrophilic polyphenols typical of *Olea europaea* L. [14]. The International Olive Council (IOC) proposes an efficient method by which experienced panelists qualify oils into categories such as EVOO [15]. These guidelines consist of assessing both positive qualities, such as pungency, bitterness, and fruitiness (green and ripe), and negative characteristics, such as rancidity, winey flavor, wet wood, and vinegary flavor [13,16].

Deep-frying (D-F) is one of the world’s most widespread culinary procedures, both for industrial and domestic food preparation purposes. During D-F, foodstuffs are immersed in heated oil at 150–190 °C. In the presence of O_2_, numerous complex reactions take place, e.g., hydrolysis, oxidation, and polymerization [17]. On the other hand, changes in chemical, functional, and sensory attributes (which are associated with minor hydrophilic and lipophilic phenolic compounds and volatile organic substances) of EVOOs can occur during D-F, mainly due to oxidative processes induced by the high thermal process [18]. These damaging oxidative reactions affect triglycerides, volatile compounds, and polyphenol content, consequently preceding modifications in the sensorial characteristics, including the deterioration of fresh notes and inception of defects and limiting the EVOO shelf-life, which is often downgraded to the lower olive oil category [5,14].

In this context, some questions are still unanswered: How long do the sensory properties of EVOOs rich in phenolic compounds (~650 mg/kg) under D-F at several conditions remain stable? What are the main sensory markers in natural exogenous bio-phenol-rich EVOOs under the best high thermal processing?

Recently, Pierguidi et al. [13] investigated the markers of sensory dynamics in VOOs enriched with polyphenols under optimal storage conditions. On the other hand, and to the best of our knowledge, no reports are available in the literature regarding the sensorial analyses of EVOO under D-F and/or with or without added olive fruit extract (OFE). Therefore, to answer the above research questions, the current study aims to prepare functional EVOO by supplementing original EVOO with OFE rich in HTyr as a source of natural phenolic compounds. A comparison between several varieties of EVOOs with Orujo oil, olive oil 0.4° and olive oil 1°, sunflower oil, and sunflower oil high-oleic acid was also conducted. Moreover, the sensorial markers by PT of the supplemented prepared olive oils after D-F that were enriched with OFE were thoroughly evaluated and compared with controls (original olive oil, supplemented olive oil, and mixed original and supplemented olive oil). This is a standpoint to bridge the gap between the low positive sensorial attributes of deep-fried olive oil and the improved sensorial profile of deep-fried olive oils, broadening the knowledge regarding the preservation and the self-life of deep-fried olive oils. Finally, PT analyses combined with PCA are employed in the current study to define the best D-F conditions of each investigated EVOO variety, along with other olive oil varieties and two sunflower oil categories.

## 2. Materials and Methods

### 2.1. Sensory Analyses Materials

Watch glasses, and standard cups according to the COI/T.20/Doc. No.15/Rev. 10, (2018) standard [15], odorless and indelible markers to mark the tasting glasses, tasting sheets, apples, bottle of water at room temperature, air oven (Super M 1072, Barcelona, Spain) to temper the samples and for maintaining the temperature of the sample at 28 ± 2 °C, balance (Sartorius AG120S, Göttingen, Germany) with precision of ±0.1 g, thermometer to control of room temperature, and thermometric probe to measure the temperature of the samples were used in the sensory analyses.

### 2.2. Olive Fruit Extract (OFE) and Oil Sampling

In total, 1 kg of olive fruit dry extract (20% hydroxytyrosol) was obtained from Natac BioTech with code number N20100126, and batch number 202304121627 (Natac, HQ-Europe, Alcorcon, Madrid, Spain). The extract was obtained in a sealed package, protected from light, and stored at 7 °C ± 2 for further processing and analysis.

Several vegetable oils including four categories from olive oil and two categories from sunflower oil were investigated as follows: (1) nine various EVOOs were selected in this study: cv. Picual (La casa del aceite, S.L. Cascante, Navarra, Spain), cv. Cornicabra (Aceite del Sur, COOSUR, Vilches, Jaen, Spain), cv. Empeltre (La casa del aceite, Navarra, Spain), cv. Arbequina (Aceite del Sur, COOSUR, Vilches, Jaen, Spain), cv. Hojiblanca (Aceite del Sur, COOSUR, Vilches, Jaen, Spain), cv. Manzanilla Cacereña (Aceite Artajo, Finca Los Llanos s/n, Fontellas, Navarra, Spain), cv. Royuela/Arróniz (Aceite Artajo, Finca Los Llanos s/n, Fontellas, Navarra, Spain), cv. Koroneiki (Aceite Artajo, Finca Los Llanos s/n, Fontellas, Navarra, Spain), cv. Arbosana (Aceite Artajo, Finca Los Llanos s/n, Fontellas, Navarra, Spain); (2) one EVOO mixed with ROO namely olive oil 1° (1° corresponds to a maximum acidity equal 1) (obtained from La Masia, Oleo Masia, S.A. Sevilla, Spain); (3) one Orujo refined olive oil mixed with EVOO namely Orujo oil (simply, Spain); (4) one virgin olive oil mixed with ROO, namely olive oil 0.4° (0.4° corresponds to a maximum acidity equal 0.4) (purchased from La Española oils, Seville Spain); (5) one refined sunflower oil (Abaco, Tarragona, Spain); and (6) one refined sunflower oil-high oleic acid (Mercadona, Hacendado, Logroño, Spain).

**Figure 1:**
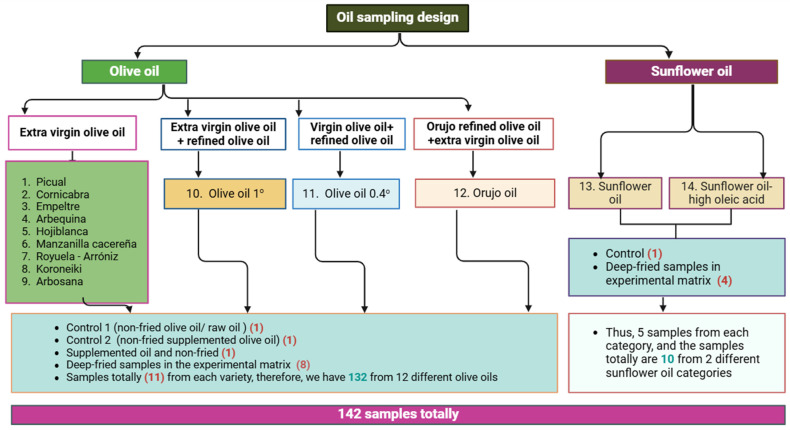
Research flow chart of distribution of olive oil and sunflower samples from various categories.

Therefore, 11 samples from each olive oil category were investigated, including Control 1 (original non-fried olive oil), Control 2 (non-fried mixed original olive oil with supplemented olive oil with OFE, supplemented non-fried olive oil, and 8 samples that underwent D-F experiments under different conditions (time, temperature, and polyphenols addition). Meanwhile, 5 samples were studied from each sunflower category. Thus, 142 samples in this study were acquired from all oils, including oils under the D-F experiment and controls. Figure 1 illustrates the sampling plan in the current study.

### 2.3. Supplementation of Olive Oil with Olive Fruit Extract

OFE was used to reinforce olive oil samples. OFE is a natural source of hydroxytyrosol and its derivatives. Exogenous OFEs are soluble in water and only slightly soluble in oil due to the polarity of hydroxytyrosol. Before studying the polyphenolic profile contained in the supplemented olive oil used to reinforce the same olive oil variety, it is necessary to know in detail the phenolic content of the oils to be reinforced, as the polyphenol profiles of olive oil varieties can vary significantly in both amount and composition.

The optimized conditions to resolve HTyr and its derivatives in olive oil samples and to obtain a high phenolics ratio, developed by our research group were as follows: Firstly, an aqueous solution was prepared from the OFE rich in HTyr (10% HTyr/H_2_O *w*/*w*). Thus, 40 g of hydroxytyrosol extract was added to 400 g of H_2_O. After that, the solution was stirred mechanically using a magnetic stirrer (RET Control-Visc, IKA-WERKE, GMBH&CO.KG, D-79219, Staufen, Germany) at room temperature for 30 min. Further, 200 g of this prepared solution was added to 500 g of nine extra virgin olive oils and other olive oil samples, and it was stirred mechanically at room temperature for 60 min. The prepared emulsion was then centrifuged at 9961× *g* for 20 min with an SLA-1500 rotor using a centrifuge (Thermo electron corporation, Model Sorvall RC-6 Plus, 37520, Osterode, Germany). Additionally, the oil was separated from the aqueous phase, and the remaining aqueous phase could be reused for new reinforcements. Furthermore, the supplemented olive oil (supernatant) was transferred to an amber bottle and kept at 7 °C ± 2 for further experiments and analysis. Each individual original olive oil variety/category was supplemented up to 650 mg/kg (this concentration was selected to simulate oils that naturally contain a high amount of polyphenols) from the prepared supplemented olive oil, depending on the total polyphenol content (TPC) of each olive oil sample after the quantification by high-performance liquid chromatography (HPLC).

### 2.4. HPLC Analysis to Determine TPC

The total phenolic content of each original olive oil, the supplemented olive oil, as well as the mixed samples between the supplemented and the original oil, up to 650 mg/kg was quantified according to the procedure of the International Olive Council [19] using the HPLC system. Chromatographic analysis was performed by Hewlett Packard 1100 series system (Agilent Technologies, Germany) allied with a high-pressure gradient pump, a photodiode array detector (DAD), and a Spherisorb octadecyl silyl 2 (ODS) chromatographic column (250 mm ×4.6 id, 5.0 μm particle size, supplied by Waters, Dublin, Ireland). The analyses were adjusted at 25 °C. Then, 20 µL of each oil sample was injected and analyzed in triplicates. The separation was carried out using a mobile phase of 0.2% H_3_PO_4_/methanol/acetonitrile. Furthermore, 20 µL of the external calibration standard solution (tyrosol and syringic acid) was injected and the chromatogram at 280 nm was recorded. The total phenolic content was calculated by summing the areas of the different chromatographic peaks using the following equation:

$$TPC\;\left(\frac{mg}{kg}\right)=\frac{\left(\Sigma \;areas\;of\;the\;peaks\right)\times 1.000\times \frac{RRF\;syringic\;acid}{tyrosol}\times \left(Weight\;of\;syringic\;acid\right)}{\left(Area\;of\;syringic\;acid\right)\times \left(Weight\;of\;the\;sample\right)}$$

where, RRF syringic acid/tyrosol: the multiplication coefficient to express the results in tyrosol.

Moreover, the polyphenolic concentration of each original olive oil, the supplemented olive oil, as well as the mixed samples between the supplemented and the original oil, up to 650 mg/kg, is shown in Table 1.

### 2.5. Full Factorial Experimental Design

Based on the preliminary experiments conducted by our research group (EVOO was fried at various times, i.e., 3, 6, 12, 18, 24, and 48 h, at two different temperatures, i.e., 170 and 210 °C). The prolonged frying time above 6 h had a great negative effect on the polyphenolic profile and sensory properties of EVOO. Therefore, extended frying times at both temperatures were not considered in the factorial design experiment. 

A full factorial experimental design (2^3^ design) for olive oil categories and a 2^2^ design for sunflower oil categories were employed in this study, as described by Box et al. [20]. To explain such an influence, it was essential to measure both the direct impact of the experimental variables and their potential interaction impact. The experimental design delineating the factors and corresponding levels for each factor for each olive oil category is shown in Table S1, while Table S2 presents the experimental design for each sunflower oil category. 

Three mixture design factors—frying time (x_l_), frying temperature (x_2_), and added natural extract enriched with polyphenols particularly hydroxytyrosol and its derivatives (x_3_)—were selected for the experiments in olive oil categories. Meanwhile, frying time and frying temperature were chosen for sunflower oil categories because sunflower oil does not contain phenolic content (Table 1). Two coded levels (−1 and +1) were set for each factor to carry out the analyses, where 3 and 6 h for frying time, 170 and 210 °C for frying temperature, and 0 and 650.0 mg/kg for polyphenols addition, respectively. The lower temperature, 170 °C, was set according to the procedures of Casal et al. [21]. Additionally, 0 mg/kg polyphenols addition (Table S1) indicates that there is no exogenous OFE addition in specific experiments, leaving only the original polyphenol content, which varied widely among the samples, as illustrated in Table 1. 

Furthermore, the linear regression model of the factors of each response in each oil category was calculated as follows:

y = b_0_ + b_1_x_1_ + b_2_x_2_ + b_3_x_3_ + b_12_x_1_x_2_ + b_13_x_1_x_3_ + b_23_x_2_x_3_ + b_123_x_1_x_2_x_3_ (1)

where y is the predicted value of the response. The linear regression coefficients are as follows: b_0_ is the mean of the response; b_1_, b_2_, and b_3_ are the main effects of the independent variables; b_12_, b_13_, b_23_, and b_123_ are the interaction effects of the independent variables. Moreover, x_l_, x_2_, and x_3_ are coded variables (time, temperature, and polyphenol addition, respectively) for the experimental design in the D-F process. 

For modeling purposes, the mathematical equation that models and predicts the relationship between the independent variables (time, temperature, and polyphenol addition) and the response variable (fusty, winey, wet wood, rancidity, fruity green, fruity ripe, bitter, and pungent) of each oil category under D-F is shown in Tables S3–S16.

### 2.6. Deep-Frying Experiment

Various olive oil and sunflower oil categories, as shown previously in Figure 1, were heated using a 0.5 L flask with a Soxhlet heating instrument (model J. P. SELECTA, s. a. u., Abrera, Barcelona, Spain) and equipped with an ELECTTEMP-BASIC (00-B, 50/60 Hz, J. P. SELECTA, s. a. u., Abrera, Barcelona, Spain) to control the time and monitor the temperature throughout the experiments using a thermometer. For each D-F experiment, 0.4 L of oil was placed in the fryer and subjected to continuous heating at 170 ± 10 °C for 3 or 6 h and 210 ± 10 °C for 3 or 6 h. After the D-F process of oil sample at several conditions (e.g., oil variety, frying time, frying temperature, and added natural antioxidants), a volume of 400 mL was collected in standard amber glass vials to track oil degradation during the D-F process and to determine the dynamic alterations in oxidized oil. The collected oil samples were stored at 5 °C in dark conditions to prevent further oxidation before analysis. The sensorial profile of the deep-fried oils was evaluated for controls (non-fried) and fried samples for each oil category under investigation.

### 2.7. Sensory Analysis

The sensory analysis was based on the assessment of the perception of the olfactory-gustatory stimuli of olive oil. Each taster evaluated the intensity of perception of the positive attributes present in the oil sample, as well as any negative attributes. The classification of each sample was conducted by measuring the intensity of its defects and attributes as perceived by a panel of tasters. The panelists used tasting glasses characterized by a broad base, which provided stability, and a narrow mouth, which concentrated odors for attribute identification. The official procedures described in Commission Regulation (EEC) No 2568/91 [22] with subsequent amendments [12,23], and the procedure of International Olive Council (COI/T.20/Doc. No.15/Rev. 10, (2018) [15], were followed to conduct the organoleptic assessment of the olive oil samples. The panel test was led by a panel leader and comprised 10 trained assessors. Informed consent was obtained from all individual participants included in the study. Furthermore, each taster first smelled the sample, then tasted it, and completed a tasting form recording the intensity of positive attributes and the existence of negative attributes of the oil sample. The descriptors of the negative attributes, which marked the intensity of defects, included fusty/muddy sediment, musty/humid/earthy, winey/vinegary/acid/sour, frostbitten olives (wet wood), and rancid. Additionally, the panelists assessed other negative attributes, such as metallic, dry hay, grubby, rough, brine, heated or burnt, vegetable water, esparto, cucumber, and greasy. Finally, they evaluated the positive attributes, such as green fruity, ripe fruity, bitter, and pungency, using a 10 cm unstructured line scale. The results were entered in Microsoft Excel (Office 365, Microsoft Corporation, Redmond, WA, USA), following the statistical method outlined in the IOC Standard T20 Doc. no. 15. The results were rounded to one decimal place. Additionally, the median values were computed for further statistical analyses.

### 2.8. Statistical Analyses

SPSS (SPSS version 28, IBM SPSS Statistics, Chicago, IL, USA) was used to analyze experimental data and to obtain the mean ± SD of total polyphenols. Moreover, mean sensory scores were calculated and illustrated in a spider diagram using Origin 2022 software (Origin Lab, Northampton, MA, USA). Additionally, principal component analysis (PCA), including scores and loadings (Biplot), was presented using the Origin 2022 package. To study the significant impacts of the independent coded factors on the response for the full factorial experimental designs (2^3^ design and 2^2^ design), single-factor or combined interaction factors with lower or equal absolute values than b_123_ were considered non-significant, while higher values of single factor or combined interaction factors than absolute value (b_123_) were considered significant. The mean data (responses or 19 sensory descriptors) obtained from the experimental designs of the studied deep-fried oil samples were analyzed and processed using Nemrod-W software (NEMROD-W, Conception, and treatments of experimental strategies, version 2000-D, Marseille, France), according to Mathieu et al. [24], to determine the approximate descriptions of the interactions of the factors. This was achieved by plotting the experimental data and clarifying the combined interactions between the independent variables on the mean sensory scores (responses) of each investigated oil.

## 3. Results 

### 3.1. Median Defects of Fusty/Muddy Sediment, Winey/Vinegary/Acid/Sour, and Frostbitten Olives

**Figure 2:**
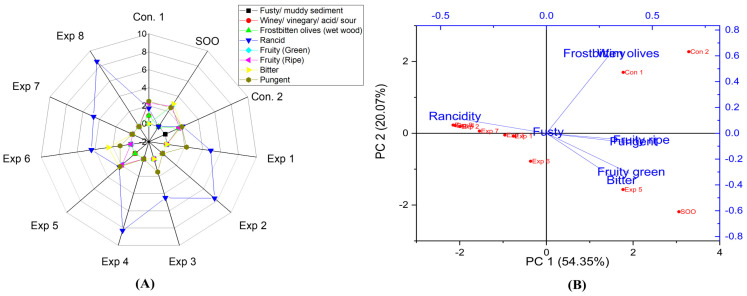
(**A**) Spider diagram of the mean sensory evaluation of EVOO cv. Hojiblanca under D-F conditions; (**B**) PCA biplot (score and loading plots) of EVOO cv. Hojiblanca under D-F conditions. Con. 1: non-fried original EVOO cv. Hojiblanca; SOO: non-fried supplemented EVOO cv. Hojiblanca with polyphenols; Con. 2: non-fried supplemented EVOO cv. Hojiblanca mixed with original EVOO cv. Hojiblanca up to 650 mg/kg of polyphenols; Exp.1: Hojiblanca deep-fried at 170 °C for 3 h without polyphenol addition; Exp. 2: Hojiblanca deep-fried at 170 °C for 6 h without polyphenol addition; Exp. 3: Hojiblanca deep-fried at 210 °C for 3 h without polyphenol addition; Exp. 4: Hojiblanca deep-fried at 210 °C for 6 h without polyphenol addition; Exp. 5: deep-fried Hojiblanca at 170 °C for 3 h with polyphenol addition; Exp. 6: Hojiblanca deep-fried at 170 °C for 6 h with polyphenol addition; Exp. 7: Hojiblanca deep-fried at 210 °C for 3 h with polyphenol addition; Exp. 8: Hojiblanca deep-fried at 210 °C for 6 h with polyphenol addition.

Based on the present findings, EVOO varieties, such as Picual, Cornicabra, Empeltre, Arbequina, Manzanilla, Royuela, Koroneiki, Arbosana, olive oil 0.4°, Orujo oil, in both fried and non-fried EVOOs (Control 1, Control 2, and supplemented olive oil), and sunflower oils did not exhibit defects such as fusty, winey, and frostbitten olives. Thus, there were no significant differences between the experimental treatments and the control. This indicates that these oils are resistant to these defects under D-F conditions, even at 210 °C for 6 h. In addition, EVOO cv. Hojiblanca in both fried and non-fried EVOO (Control 1, Control 2, and supplemented olive oil) did not exhibit median defects of fusty. However, winey and frostbitten defects were observed in the original Hojiblanca oil (Control 1) as well as in the mixed one (Control 2) with scores of 0.9 and 1.6, respectively. Therefore, Hojiblanca oil was the only EVOO that displayed these defects in non-fried olive oils (Figure 2A,B). 

**Figure 3:**
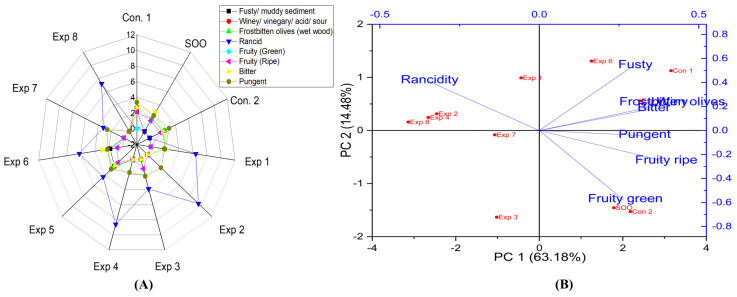
(**A**) Spider diagram of the mean sensory evaluation of olive oil 1° under D-F conditions; (**B**) PCA biplot (score and loading plots) of olive oil 1° under D-F conditions. Con. 1: non-fried original olive oil 1°; SOO: non-fried supplemented olive oil 1° with polyphenols; Con. 2: non-fried supplemented olive oil 1° mixed with original olive oil 1° up to 650 mg/kg of polyphenols; Exp.1: olive oil 1° deep-fried at 170 °C for 3 h without polyphenol addition; Exp. 2: olive oil 1° deep-fried at 170 °C for 6 h without polyphenol addition; Exp. 3: olive oil 1° deep-fried at 210 °C for 3 h without polyphenol addition; Exp. 4: olive oil 1° deep-fried at 210 °C for 6 h without polyphenol addition; Exp. 5: deep-fried olive oil 1° at 170 °C for 3 h with polyphenol addition; Exp. 6: olive oil 1° deep-fried at 170 °C for 6 h with polyphenol addition; Exp. 7: olive oil 1° deep-fried at 210 °C for 3 h with polyphenol addition; Exp. 8: olive oil 1° deep-fried at 210 °C for 6 h with polyphenol addition.

On the contrary to the EVOO varieties, olive oil 1°, in both fried and non-fried oil (Control 1, Control 2, and supplemented olive oil) exhibited median defects of fusty, winey, and frostbitten olives (Figure 3A,B). Moreover, PCA indicated that the contribution rates of PC1 and PC2 were 63.18% and 14.48%, respectively. Exp. 5, 6, and Con 1 showed a pronounced fusty characteristic (Figure 3B). A significant effect of the Temperature–Polyphenols interaction under experimental conditions was observed on fusty properties. However, there were no significant differences in the interaction between Time–Polyphenols and Time–Temperature on fusty properties (Figure S1D). In addition, there were no significant differences in the interaction between Time–Polyphenols on winey/frostbitten olives. Conversely, Time–Temperature and Temperature–Polyphenols interactions had a notable significant impact on winey/frostbitten olives properties (Figure S2D). Exp. 5, 6, and Con 1 showed pronounced winey/frostbitten olives (Figure 3B). Therefore, olive oil 1° is a low-stability oil against these mentioned defects under D-F conditions due to the inferior sensory qualities of the original oil compared to EVOO varieties.

### 3.2. Median Defects of Rancidity

**Figure 4:**
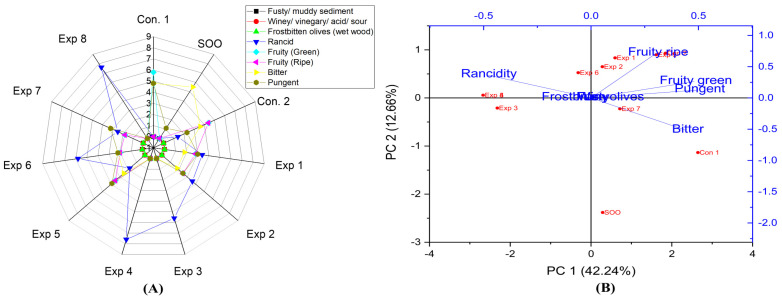
(**A**) Spider diagram of the mean sensory evaluation of EVOO cv. Picual under D-F conditions; (**B**) PCA biplot (score and loading plots) of EVOO cv. Picual under D-F conditions. Con. 1: non-fried original EVOO cv. Picual; SOO: non-fried supplemented EVOO cv. Picual with polyphenols; Con. 2: non-fried supplemented EVOO cv. Picual mixed with original EVOO cv. Picual up to 650 mg/kg of polyphenols; Exp.1: Picual deep-fried at 170 °C for 3 h without polyphenol addition; Exp. 2: Picual deep-fried at 170 °C for 6 h without polyphenol addition; Exp. 3: Picual deep-fried at 210 °C for 3 h without polyphenol addition; Exp. 4: Picual deep-fried at 210 °C for 6 h without polyphenol addition; Exp. 5: Picual deep-fried at 170 °C for 3 h with polyphenol addition; Exp. 6: Picual deep-fried at 170 °C for 6 h with polyphenol addition; Exp. 7: Picual deep-fried at 210 °C for 3 h with polyphenol addition; Exp. 8: Picual deep-fried at 210 °C for 6 h with polyphenol addition.

**Figure 5:**
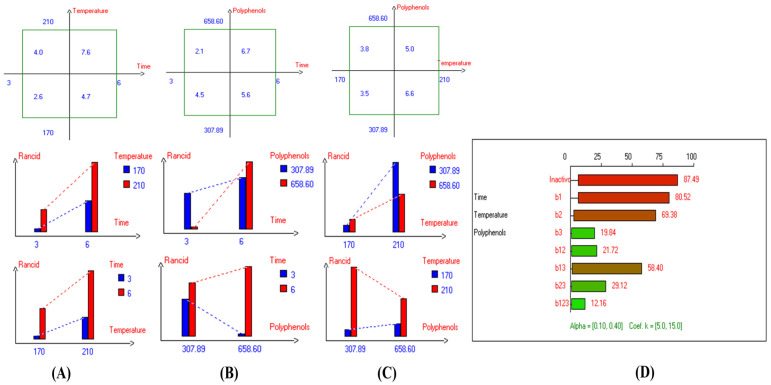
Combined interactions between the independent variables on a response variable (rancidity) in EVOO cv. Picual under D-F: (**A**) x_1_ and x_2_, (**B**) x_1_ and x_3_, (**C**) x_2_ and x_3_, and (**D**) results of variance analysis of regression equation model and the significance changes in each individual independent variable and interaction between the combined independent variables on rancidity score; b represents a significant difference when be >b_123_, while b represents no significant difference when be ≤b_123_; x_1_: time, x_2_: temperature, x_3_: polyphenols.

The present research demonstrated that OFE inhibited and/or reduced oxidation compared to controls. The best results were achieved at 170 °C for 3 h of frying with added polyphenols (658.60 mg/kg) in EVOO cv. Picual (Exp. 5) under the three investigated factors, resulting in low rancidity (1.8) (Figure 4A). Additionally, PCA showed that Exp. 4, 6, and 8 were clustered near rancidity, while Exp. 1, 2, 5, and 7 had low rancidity values. The contribution rates of PC1 and PC2 were 42.24% and 12.66%, respectively (Figure 4B). As observed from the analysis of model findings (Figure 5D), this model revealed that the three experimental factors had extremely significant effects on the rancidity level in EVOO cv. Picual. Notably, the ideal combined interaction factors were frying Picual oil at a short frying time (3 h) with the addition of the extract containing a high polyphenol content (650 mg/kg) (Figure 5B), which recorded the lowest rancidity value (2.1). This was followed by treatment where Picual oil was deep-fried for a short frying time (3 h) at a low temperature (170 °C), resulting in a rancidity value of 2.6 (Figure 5A). Conversely, a high rancidity score (7.6) was observed when Picual oil was fried for 6 h at 210 °C (Figure 5A). 

**Figure 6:**
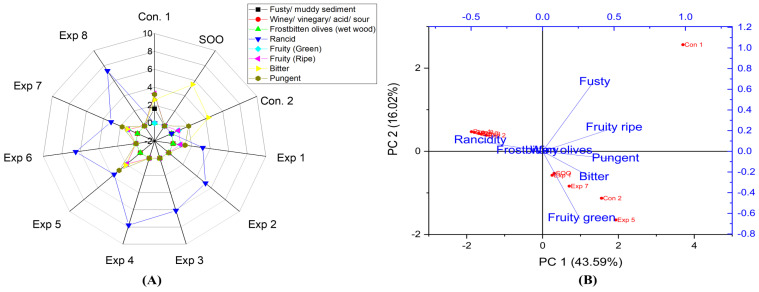
(**A**) Spider diagram of the mean sensory evaluation of EVOO cv. Cornicabra under D-F conditions; (**B**) PCA biplot (score and loading plots) of EVOO cv. Cornicabra under D-F conditions. Con. 1: non-fried original EVOO cv. Cornicabra; SOO: non-fried supplemented EVOO cv. Cornicabra with polyphenols; Con. 2: non-fried supplemented EVOO cv. Cornicabra mixed with original EVOO cv. Cornicabra up to 650 mg/kg of polyphenols; Exp. 1: Cornicabra deep-fried at 170 °C for 3 h without polyphenol addition; Exp. 2: Cornicabra deep-fried at 170 °C for 6 h without polyphenol addition; Exp. 3: Cornicabra deep-fried at 210 °C for 3 h without polyphenol addition; Exp. 4: Picual deep-fried at 210 °C for 6 h without polyphenol addition; Exp. 5: deep-fried Cornicabra at 170 °C for 3 h with polyphenol addition; Exp. 6: Cornicabra deep-fried at 170 °C for 6 h with polyphenol addition; Exp. 7: Cornicabra deep-fried at 210 °C for 3 h with polyphenol addition; Exp. 8: Cornicabra deep-fried at 210 °C for 6 h with polyphenol addition.

According to the data obtained (Figure S3D), significant differences were observed in the interaction between Time–Polyphenols factors and the interaction between Temperature-Polyphenol factors on the rancidity property in EVOO cv. Cornicabra. In contrast, there were no significant differences in the interaction between Time–Temperature factors on rancidity. Notably, the best combined factors values, which recorded the lowest rancidity, were achieved by frying Cornicabra oil at a short frying time (3 h) with the addition of OFE containing high polyphenols (658.15 mg/kg) and by frying at a short frying time (3 h) at a low temperature (170 °C), resulting in a rancidity value of 3.4 (Figure S3A,B). Conversely, a high rancidity score was observed when Cornicabra oil was deep-fried for 6 h at 210 °C, reaching a value of 7.5 (Figure S3A). This was followed by an oil sample treated at 210 °C without the addition of polyphenols (275.67 mg/kg), which exhibited a rancidity value of 6.9 (Figure S3C). The present research indicated that the best treatment to obtain deep-fried Cornicabra oil with low rancidity was achieved at 210 °C for 3 h with added exogenous polyphenols (Exp. 7), resulting in a rancidity value of 3.1. Thus, Cornicabra oil demonstrates stability at high temperatures of frying (Figure 6A). Furthermore, PCA showed that the contribution rate of PC1 and PC2 were 43.59% and 16.02%, respectively. PCA revealed that Exp. 2, 3, 4, 6, and 8 were clustered around rancidity, while Exp. 1, 5, and 7 exhibited low rancidity values (Figure 6B).

**Figure 7:**
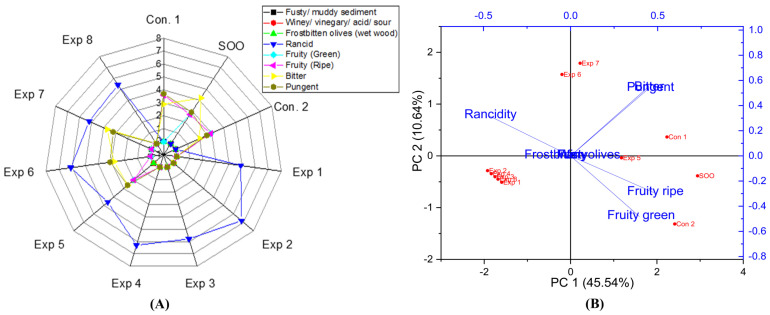
(**A**) Spider diagram of the mean sensory evaluation of EVOO cv. Empeltre under D-F conditions; (**B**) PCA biplot (score and loading plots) of EVOO cv. Empeltre under D-F conditions. Con. 1: non-fried original EVOO cv. Empeltre; SOO: non-fried supplemented EVOO cv. Empeltre with polyphenols; Con. 2: non-fried supplemented EVOO cv. Empeltre mixed with original EVOO cv. Empeltre up to 650 mg/kg of polyphenols; Exp.1: Empeltre deep-fried at 170 °C for 3 h without polyphenol addition; Exp. 2: Empeltre deep-fried at 170 °C for 6 h without polyphenol addition; Exp. 3: Empeltre deep-fried at 210 °C for 3 h without polyphenol addition; Exp. 4: Empeltre deep-fried at 210 °C for 6 h without polyphenol addition; Exp. 5: deep-fried Empeltre at 170 °C for 3 h with polyphenol addition; Exp. 6: Empeltre deep-fried at 170 °C for 6 h with polyphenol addition; Exp. 7: Empeltre deep-fried at 210 °C for 3 h with polyphenol addition; Exp. 8: Empeltre deep-fried at 210 °C for 6 h with polyphenol addition.

The interaction between all factors selected in the experiment had a significant impact on the rancidity level in EVOO cv. Empeltre (Figure S4D). Furthermore, the best combined values to obtain deep-fried oil with low rancidity were the frying at 170 °C for 3 h, which recorded the lowest rancidity value (4.7) (Figure S4A), followed by frying for 3 h, with the addition of OFE (647.23 mg/kg of polyphenols), which resulted in a rancidity value of 4.9 (Figure S4B). Conversely, a high rancidity score was observed when Empeltre oil fried for 6 h without the addition of a polyphenol extract, recording a rancidity value of 6.5 (Figure S4B), followed by Empeltre oil fried for 6 h at 170 °C, which recorded a rancidity value of 6.4 (Figure S4A). Consequently, prolonged frying times contributed significantly to the rancidity of Empeltre. Moreover, the present research demonstrated that OFE inhibited and/or reduced oxidation. The best conditions to obtain a low rancid EVOO cv. Empeltre were achieved at 170 °C for 3 h with polyphenol supplementation up to 647.23 mg/kg (Exp. 5), resulting in a rancidity value of 4.6 (Figure 7A). Furthermore, PCA analyses showed that the contribution rates of PC1 and PC2 were 45.54% and 10.64%, respectively. PCA revealed that Exp. 1, 2, 3, 4, 6, and 8 were clustered near rancidity, while Exp. 5 and 7 exhibited low rancidity values (Figure 7B).

**Figure 8:**
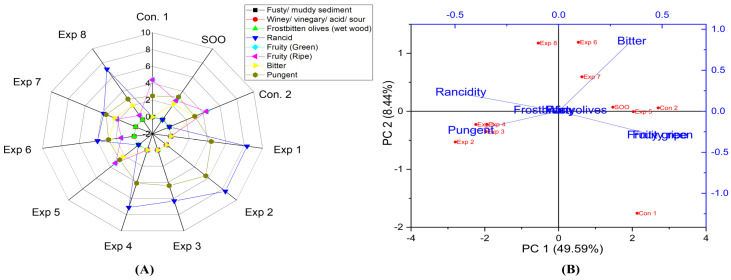
(**A**) Spider diagram of the mean sensory evaluation of EVOO cv. Arbequina under D-F conditions; (**B**) PCA biplot (score and loading plots) of EVOO cv. Arbequina under D-F conditions. Con. 1: non-fried original EVOO cv. Arbequina; SOO: non-fried supplemented EVOO cv. Arbequina with polyphenols; Con. 2: non-fried supplemented EVOO cv. Arbequina mixed with original EVOO cv. Arbequina up to 650 mg/kg of polyphenols; Exp.1: Arbequina deep-fried at 170 °C for 3 h without polyphenol addition; Exp. 2: Arbequina deep-fried at 170 °C for 6 h without polyphenol addition; Exp. 3: Arbequina deep-fried at 210 °C for 3 h without polyphenol addition; Exp. 4: Arbequina deep-fried at 210 °C for 6 h without polyphenol addition; Exp. 5: deep-fried Arbequina at 170 °C for 3 h with polyphenol addition; Exp. 6: Arbequina deep-fried at 170 °C for 6 h with polyphenol addition; Exp. 7: Arbequina deep-fried at 210 °C for 3 h with polyphenol addition; Exp. 8: Arbequina deep-fried at 210 °C for 6 h with polyphenol addition.

Based on the current findings (Figure S5D), significant differences were observed in the interaction between Time–Polyphenols factors. However, there was no significant difference in the combined effect of Time–Temperature factors on the rancidity. Conspicuously, the best conditions resulting in low rancidity were achieved by frying Arbequina oil for 3 h with added polyphenols (663.96 mg/kg), recording the lowest rancidity value (1.9) (Figure S5B), followed by frying at 170 °C with added polyphenols, which resulted in a rancidity value of 2.0 (Figure S5C). Conversely, a high rancidity score was observed when the oil was fried at 170 °C without the addition of extract, reaching a rancidity value of 8.3 (Figure S5C). This was followed by oil fried for 3 h without the addition of polyphenol extract (227.71 mg/kg), which reached a rancidity value of 7.7 (Figure S5B). Thus, OFE inhibits oxidation in oil samples under D-F. The best conditions to obtain a low-rancid EVOO were achieved at 170 °C for 3 h with supplementation polyphenol supplementation (663.96 mg/kg), which recorded a value of 0.0 (Figure 8A). One of the most interesting results in this research was that EVOO cv. Arbequina was the best choice amongst the studied varieties to obtain oil without rancidity under D-F conditions. Additionally, PCA also showed that Exp. 5 had the lowest rancidity among all treatments (Figure 8B). PCA showed that the contribution rates of PC1 and PC2 were 49.59% and 8.44%, respectively.

The combined interaction between Time–Temperature and Temperature–Polyphenols factors showed a significant variance, whereas Time–Polyphenols combined factors did not affect the rancidity property (Figure S6D). Furthermore, the optimum conditions were achieved by frying Hojiblanca oil at 170 °C with added OFE, recording the lowest rancidity value (3.3) (Figure S6C), followed by frying for 3 h, with added polyphenols (655.73 mg/kg) with a value of 3.5 (Figure S6B). Conversely, a high rancidity score was observed when Hojiblanca oil was deep-fried at 210 °C for 6 h, reaching a value of 8.4 (Figure S6A). This was followed by oil fried for 6 h without polyphenol supplementation (209.51 mg/kg) which recorded a rancidity value of 8.0 (Figure S6B). It can be stated that Hojiblanca is a non-stable oil under high temperatures for prolonged periods. However, the present research indicated that OFE inhibited or reduced oxidation. The best conditions to obtain a low-rancid EVOO cv. Hojiblanca were achieved at 170 °C for 3 h with supplementation polyphenols (650 mg/kg), resulting in a rancidity value of 2.3 (Exp. 5) (Figure 2A). Furthermore, PCA revealed that Exp. 5 was the best treatment among all treatments, along with Con 1, Con 2, and SOO samples (Figure 2B). PCA plots showed that the contribution rates of PC1 and PC2 were 54.35% and 20.07%, respectively. However, Exp. 1, 2, 3, 4, 6, 7, and 8 showed close distribution distances around rancidity.

**Figure 9:**
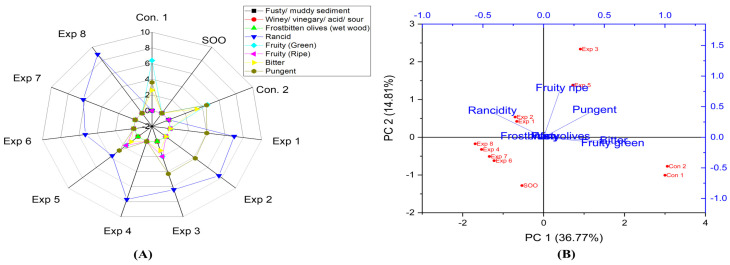
(**A**) Spider diagram of the mean sensory evaluation of EVOO cv. Manzanilla under D-F conditions; (**B**) PCA biplot (score and loading plots) of EVOO cv. Manzanilla under D-F conditions. Con. 1: non-fried original EVOO cv. Manzanilla; SOO: non-fried supplemented EVOO cv. Manzanilla with polyphenols; Con. 2: non-fried supplemented EVOO cv. Manzanilla mixed with original EVOO cv. Manzanilla up to 650 mg/kg of polyphenols; Exp. 1: Manzanilla deep-fried at 170 °C for 3 h without polyphenol addition; Exp. 2: Manzanilla deep-fried at 170 °C for 6 h without polyphenol addition; Exp. 3: Manzanilla deep-fried at 210 °C for 3 h without polyphenol addition; Exp. 4: Manzanilla deep-fried at 210 °C for 6 h without polyphenol addition; Exp. 5: deep-fried Manzanilla at 170 °C for 3 h with polyphenol addition; Exp. 6: Manzanilla deep-fried at 170 °C for 6 h with polyphenol addition; Exp. 7: Manzanilla deep-fried at 210 °C for 3 h with polyphenol addition; Exp. 8: Manzanilla deep-fried at 210 °C for 6 h with polyphenol addition.

According to the present results (Figure S7D), significant differences were observed in all the studied combined interactions regarding the rancidity property. Notably, the best combined values were achieved by frying Manzanilla oil at 170 °C with added polyphenols (661.28 mg/kg), recording the lowest rancidity value (4.5) (Figure S7C). On the other hand, a high rancidity score was observed when Manzanilla oil was deep-fried at 210 °C for 6 h, reaching a value of 8.3 (Figure S7A). The best conditions to obtain low-rancid EVOO cv. Manzanilla were achieved at 170 °C for 3 h with polyphenol supplementation (661.28 mg/kg), resulting in a rancidity value of 3.7 (Exp. 5) (Figure 9A). Thus, the present research demonstrated that OFE inhibited or reduced oxidation. Furthermore, PCA revealed that the contribution rates of PC1 and PC2 were 36.77% and 14.81%, respectively. In addition, Exp. 1, 2, 4, and 8 showed close distribution distances around rancidity (Figure 9B).

**Figure 10:**
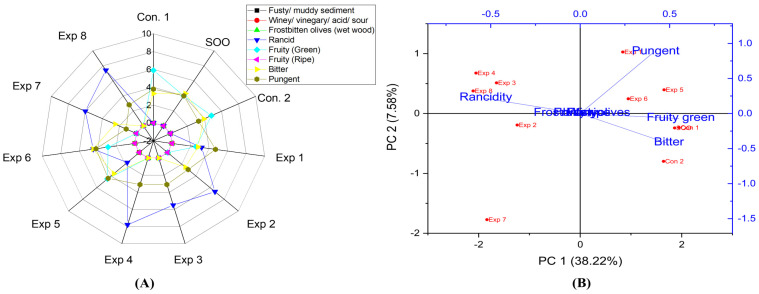
(**A**) Spider diagram of the mean sensory evaluation of EVOO cv. Royuela under D-F conditions; (**B**) PCA biplot (score and loading plots) of EVOO cv. Royuela under D-F conditions. Con. 1: non-fried original EVOO cv. Royuela; SOO: non-fried supplemented EVOO cv. Royuela with polyphenols; Con. 2: non-fried supplemented EVOO cv. Royuela mixed with original EVOO cv. Royuela up to 650 mg/kg of polyphenols; Exp. 1: Royuela deep-fried at 170 °C for 3 h without polyphenol addition; Exp. 2: Royuela deep-fried at 170 °C for 6 h without polyphenol addition; Exp. 3: Royuela deep-fried at 210 °C for 3 h without polyphenol addition; Exp. 4: Royuela deep-fried at 210 °C for 6 h without polyphenol addition; Exp. 5: deep-fried Royuela at 170 °C for 3 h with polyphenol addition; Exp. 6: Royuela deep-fried at 170 °C for 6 h with polyphenol addition; Exp. 7: Royuela deep-fried at 210 °C for 3 h with polyphenol addition; Exp. 8: Royuela deep-fried at 210 °C for 6 h with polyphenol addition.

The interaction between frying time, frying temperature, and polyphenols had significant effects on the rancidity level in EVOO cv. Royuela (Figure S8D). Notably, the best combined values were achieved by frying Royuela oil at 170 °C for 3 h, recording the lowest rancidity value (2.4) (Figure S8A), followed by frying at 170 °C with added OFE (662.38 mg/kg of polyphenols), which resulted in a rancidity value of 3.1 (Figure S8C). By contrast, a high rancidity score was observed when Royuela oil was treated at 210 °C for 6 h, reaching 7.6 (Figure S8A). This was followed by an oil sample fried for 6 h without the addition of polyphenol extract (400.63 mg/kg), which recorded a rancidity value of 7.2 (Figure S8B). Thus, the present research demonstrated that OFE inhibited or reduced oxidation. The best conditions to obtain a low-rancid EVOO cv. Royuela were achieved at 170 °C for 3 h with polyphenol supplementation (662.38 mg/kg), resulting in a rancidity value of 1.7 (Exp. 5) (Figure 10A). Additionally, PCA showed that the contribution rates of PC1 and PC2 were 38.22% and 7.58%, respectively. Exp. 1, 5, and 6 showed low rancidity, while other treatments presented clustering near rancidity (Figure 10B).

**Figure 11:**
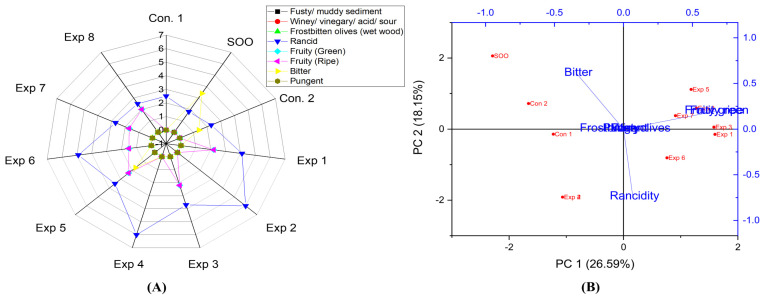
(**A**) Spider diagram of the mean sensory evaluation of Orujo oil under D-F conditions; (**B**) PCA biplot (score and loading plots) of Orujo oil under D-F conditions. Con. 1: non-fried original Orujo oil; SOO: non-fried supplemented Orujo oil with polyphenols; Con. 2: non-fried supplemented Orujo oil mixed with original Orujo oil up to 650 mg/kg of polyphenols; Exp. 1: Orujo oil deep-fried at 170 °C for 3 h without polyphenol addition; Exp. 2: Orujo oil deep-fried at 170 °C for 6 h without polyphenol addition; Exp. 3: Orujo oil deep-fried at 210 °C for 3 h without polyphenol addition; Exp. 4: Orujo oil deep-fried at 210 °C for 6 h without polyphenol addition; Exp. 5: deep-fried Orujo oil at 170 °C for 3 h with polyphenol addition; Exp. 6: Orujo oil deep-fried at 170 °C for 6 h with polyphenol addition; Exp. 7: Orujo oil deep-fried at 210 °C for 3 h with polyphenol addition; Exp. 8: Orujo oil deep-fried at 210 °C for 6 h with polyphenol addition.

In addition, significant differences were observed in the interaction between Time–Polyphenols, and Temperature–Polyphenols factors on the rancidity property of Orujo oil, whereas no significant difference was observed in the interaction between Time–Temperature factors (Figure S9D). Notably, the best combined conditions were achieved by frying Orujo oil at 210 °C with added polyphenols, recording the lowest rancidity value (2.6) (Figure S9C), followed by frying for 3 with added olive fruit extract, resulting in a rancidity value of 3.1 (Figure S9C). Therefore, the present research indicated that Orujo oil is highly stable under D-F at high temperatures when supplemented with OFE. The best conditions to obtain a low-rancid Orujo oil were achieved at 210 °C for 6 h with polyphenol supplementation (652.25 mg/kg), resulting in a rancidity value of 2.5 (Exp. 8) (Figure 11A). Additionally, PCA showed that the contribution rates of PC1 and PC2 were 26.59% and 18.15%, respectively. Exp. 2, 4, and 6 clustered near rancidity, whereas, Exp 1, 3, 5, and 7 exhibited low rancidity values along with SOO, Con 1, and Con 2 (Figure 11B). Thus, Orujo oil emerged as one of the most thermally stable olive oils under high-temperature conditions for prolonged periods when supplemented with exogenous polyphenols.

**Figure 12:**
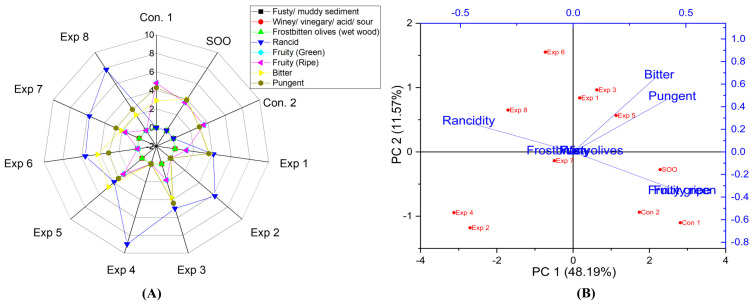
(**A**) Spider diagram of the mean sensory evaluation of EVOO cv. Koroneiki under D-F condition; (**B**) PCA biplot (score and loading plots) of EVOO cv. Koroneiki under D-F conditions. Con. 1: non-fried original EVOO cv. Koroneiki; SOO: non-fried supplemented EVOO cv. Koroneiki with polyphenols; Con. 2: non-fried supplemented EVOO cv. Koroneiki mixed with original EVOO cv. Koroneiki up to 650 mg/kg of polyphenols; Exp. 1: Koroneiki deep-fried at 170 °C for 3 h without polyphenol addition; Exp. 2: Koroneiki deep-fried at 170 °C for 6 h without polyphenol addition; Exp. 3: Koroneiki deep-fried at 210 °C for 3 h without polyphenol addition; Exp. 4: Koroneiki deep-fried at 210 °C for 6 h without polyphenol addition; Exp. 5: Koroneiki deep-fried at 170 °C for 3 h with polyphenol addition; Exp. 6: Koroneiki deep-fried at 170 °C for 6 h with polyphenol addition; Exp. 7: Koroneiki deep-fried at 210 °C for 3 h with polyphenol addition; Exp. 8: Koroneiki deep-fried at 210 °C for 6 h with polyphenol addition.

Regarding EVOO cv. Koroneiki, significant differences were observed in the interaction between Time–Temperature and Time–Polyphenols independent variables, whereas the Time–Temperature combined interaction had no significant impact on the rancidity (Figure S10D). Notably, the best combined values with low rancidity were achieved by frying EVOO cv. Koroneiki oil at 170 °C for 3 h, recording the lowest rancidity value (4.0) (Figure S10A). In contrast, a high rancidity score was observed when Koroneiki oil was fried at 210 °C for 6 h, reaching 8.4 (Figure S10A). This indicates that prolonged exposure to high temperatures gradually increases rancidity in Koroneiki oil. The best conditions to obtain a low-rancid EVOO cv. Koroneiki oil was achieved at 170 °C for 3 h with polyphenol supplementation (663.93 mg/kg), resulting in a rancidity value of 3.9 (Exp. 5) (Figure 12A). Thus, the present research indicated that OFE inhibited or reduced oxidation. Furthermore, PCA revealed that the contribution rates of PC1 and PC2 were 48.19% and 11.57%, respectively. Exp. 2, 4, 6, and 8 presented high rancidity values (Figure 12B). 

**Figure 13:**
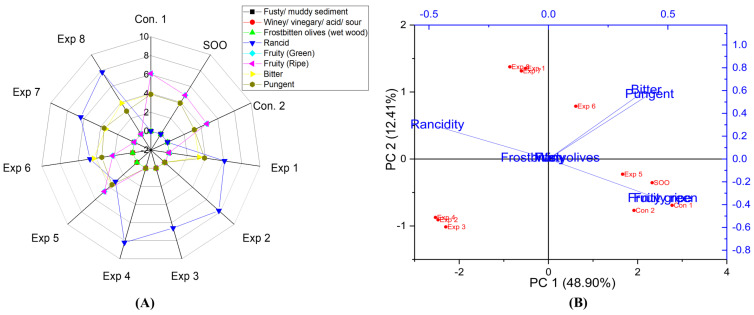
(**A**) Spider diagram of the mean sensory evaluation of EVOO cv. Arbosana under D-F conditions; (**B**) PCA biplot (score and loading plots) of EVOO cv. Arbosana under D-F conditions. Con. 1: non-fried original EVOO cv. Arbosana; SOO: non-fried supplemented EVOO cv. Arbosana with polyphenols; Con. 2: non-fried supplemented EVOO cv. Arbosana mixed with original EVOO cv. Arbosana up to 650 mg/kg of polyphenols; Exp. 1: Arbosana deep-fried at 170 °C for 3 h without polyphenol addition; Exp. 2: Arbosana deep-fried at 170 °C for 6 h without polyphenol addition; Exp. 3: Arbosana deep-fried at 210 °C for 3 h without polyphenol addition; Exp. 4: Arbosana deep-fried at 210 °C for 6 h without polyphenol addition; Exp. 5: deep-fried Arbosana at 170 °C for 3 h with polyphenol addition; Exp. 6: Arbosana deep-fried at 170 °C for 6 h with polyphenol addition; Exp. 7: Arbosana deep-fried at 210 °C for 3 h with polyphenol addition; Exp. 8: Arbosana deep-fried at 210 °C for 6 h with polyphenol addition.

The results presented that there is a significant difference in the interaction between Temperature–Polyphenols factors on pungent properties in EVOO cv. Arbosana. In contrast, there were no significant differences in the interaction between Time–Temperature and Time–Polyphenols factors (Figure S11D). Remarkably, the best combined conditions were achieved by frying Arbosana oil at 170 °C with added polyphenols, recording the lowest rancidity value (3.9) (Figure S11C), followed by frying at 170 °C for 3 h (4.6) (Figure S11A). Conversely, a high rancidity score was observed in samples fried at 210 °C for 6 h, recording a value of 8.0 (Figure S11A). The best conditions to obtain a low-rancid EVOO cv. Arbosana were achieved at 170 °C for 3 h with polyphenols supplementation (666.95 mg/kg), which recorded a value of 3.1 (Exp. 5) (Figure 13A). Additionally, PCA revealed that the contribution rates of PC1 and PC2 were 48.90% and 12.41%, respectively. Exp. 1, 2, 3, 4, 7, and 8 presented high rancidity, whereas Exp 5 and 6 exhibited low rancidity (Figure 13B). 

Frying time, frying temperature, and polyphenols had significant effects on the rancidity level in olive oil 1° (Figure S12D). The best combined conditions were achieved by frying olive oil at 1° at 210 °C for 3 h, recording the lowest rancidity value (3.5) (Figure S12A), followed by frying for 3 h with added olive fruit extract, resulting in a rancidity value of 3.7 (Figure S12B). By contrast, a high rancidity score was observed when olive oil was 1° fried for 6 h without adding polyphenols, reaching 9.0 (Figure S12B). Thus, the present research indicated that OFE plays a crucial role in reducing oxidation. The best conditions to obtain a low-rancid oil were achieved at 210 °C for 3 h with added polyphenols (650 mg/kg), resulting in a rancidity value of 3.2 (Exp 7) (see Figure 3A). Additionally, PCA showed that Exp. 2, 4, and 8 clustered near rancidity (Figure 3B). This indicates that olive oil 1° can be deep-fried at high temperatures (210 °C) with added OFE. However, without exogenous polyphenols, olive oil 1° exhibited the highest rancid value among all studied oils.

**Figure 14:**
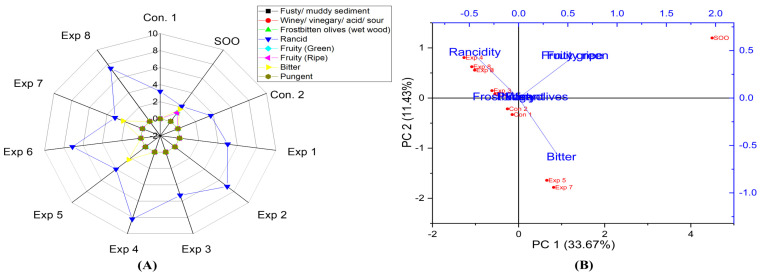
(**A**) Spider diagram of the mean sensory evaluation of olive oil 0.4° under D-F conditions; (**B**) PCA biplot (score and loading plots) of olive oil 0.4° under D-F conditions. Con. 1: non-fried original olive oil 0.4°, SOO: non-fried supplemented olive oil 0.4° with polyphenols; Con. 2: non-fried supplemented olive oil 0.4° mixed with original olive oil 0.4° up to 650 mg/kg of polyphenols; Exp. 1: olive oil 0.4° deep-fried at 170 °C for 3 h without polyphenol addition; Exp. 2: olive oil 0.4° deep-fried at 170 °C for 6 h without polyphenol addition; Exp. 3: olive oil 0.4° deep-fried at 210 °C for 3 h without polyphenol addition; Exp. 4: olive oil 0.4° deep-fried at 210 °C for 6 h without polyphenol addition; Exp. 5: deep-fried olive oil 0.4° at 170 °C for 3 h with polyphenol addition; Exp. 6: olive oil 0.4° deep-fried at 170 °C for 6 h with polyphenol addition; Exp. 7: olive oil 0.4° deep-fried at 210 °C for 3 h with polyphenol addition; Exp. 8: olive oil 0.4° deep-fried at 210 °C for 6 h with polyphenol addition.

The results also indicated that all interactions between the factors selected in the experiment had significant effects on the rancidity level in olive oil at 0.4° (Figure S13D). Notably, the best combined conditions were frying the oil for 3 h with added polyphenols, recording the lowest rancidity value (3.6) (Figure S13B). The best conditions to obtain low-rancid olive oil at 0.4° were achieved at 210 °C for 3 h with added polyphenols (650.87 mg/kg), resulting in a rancidity value of 3.1 (Exp. 7) (Figure 14A). The contribution rates of PC1 and PC2 were 33.67% and 11.43%, respectively. Exp. 2, 4, 6, and 8 presented clustering near rancidity, whereas Exp 1, 3, 5, and 7 exhibited low rancidity values, along with SOO, Con 1, and Con 2 (Figure 14B). Thus, olive oil 0.4 emerged as one of the most thermally stable olive oils under high temperatures, but for short durations with added exogenous polyphenols.

**Figure 15:**
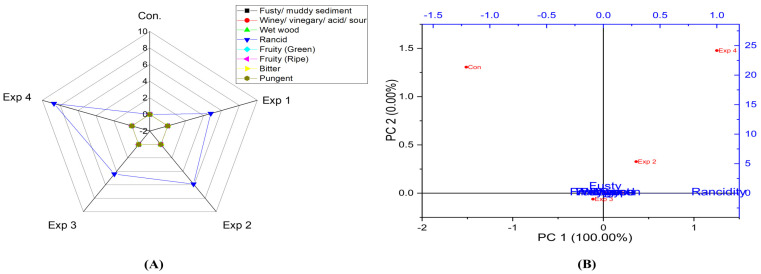
(**A**) Spider diagram of the mean sensory evaluation of sunflower oil under D-F conditions; (**B**) PCA biplot (score and loading plots) of sunflower oil under D-F conditions. Con.: non-fried original sunflower oil; Exp. 1: sunflower oil deep-fried at 170 °C for 3 h; Exp. 2: sunflower oil deep-fried at 170 °C for 6 h; Exp. 3: sunflower oil deep-fried at 210 °C for 3 h; Exp. 4: sunflower oil deep-fried at 210 °C for 6 h.

**Figure 16:**
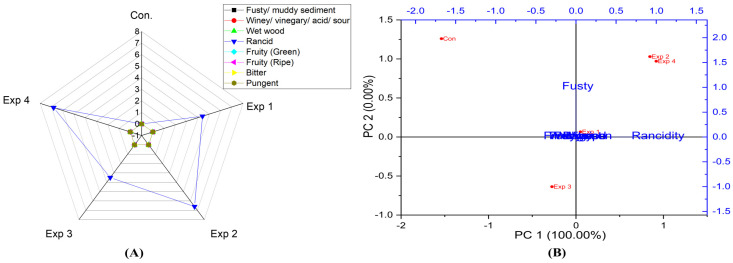
(**A**) Spider diagram of the mean sensory evaluation of sunflower oil-high oleic acid under D-F conditions; (**B**) PCA biplot (score and loading plots) of sunflower oil under D-F conditions. Con.: non-fried original sunflower oil; Exp. 1: sunflower oil deep-fried at 170 °C for 3 h; Exp. 2: sunflower oil deep-fried at 170 °C for 6 h; Exp. 3: sunflower oil deep-fried at 210 °C for 3 h; Exp. 4: sunflower oil deep-fried at 210 °C for 6 h.

According to the analysis model results (Figure S14B), the model revealed that frying time had significant effects on the rancidity level in sunflower oil. However, frying temperature did not affect rancidity. Notably, the best conditions were achieved by frying sunflower oil at 210 °C for 3 h, recording the lowest rancidity value (4.4) (Figure S14A). In contrast, a high rancidity score was observed when sunflower oil was deep-fried at 210 °C for 6 h, reaching a value of 8.7 (Figure S14A). Moreover, PCA showed that the contribution rates of PC1 and PC2 were 100.00% and 0.0%, respectively. Therefore, PC1 was entirely explained by rancidity values. Additionally, Exp. 2 and 4 clustered near rancidity, whereas Exp 1, 3, and the control exhibited low rancidity values (Figure 15A,B). Furthermore, frying time and frying temperature significantly affected the rancidity level in sunflower oil-high oleic acid (Figure S14D). The best combined conditions were achieved by frying sunflower oil-high oleic acid oil at 210 °C for 3 h (Exp. 3), recording the lowest rancidity value (3.5) (Figure S14C). Oppositely, a high rancidity score was observed when SOHO was deep-fried at 210 °C for 6 h (Exp. 4), reaching a value of 6.8 (Figure S14C). PC1 was entirely explained by rancidity values (100%). Moreover, the radar chart and PCA showed that Exp 2 and 4 had high rancidity (Figure 16A,B). Generally, the present research demonstrated that SOHO had lower rancidity under high temperatures (210 °C) and was more stable than SO. Interestingly, and as expected, sunflower oil and sunflower-high oleic acid oil did not exhibit fruity green and fruity ripe, bitter, and pungent properties.

### 3.3. Fruity Green and Ripe

**Figure 17:**
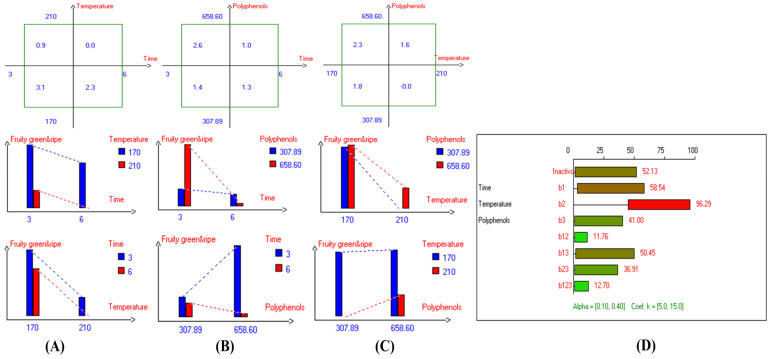
Combined interactions between the independent variables on response variable (fruity green& ripe) in EVOO cv. Picual under D-F: (**A**) x_1_ and x_2_, (**B**) x_1_ and x_3_, (**C**) x_2_ and x_3_, and (**D**) results of variance analysis of regression equation model and the significance changes in each individual independent variable and interaction between the combined independent variables on fruity green& ripe scores; b represents a significant difference when be >b_123_, while b represents no significant difference when be ≤b_123_; x_1_: time, x_2_: temperature, x_3_: polyphenols.

From the present research results, significant differences were observed in the interaction between Time–Polyphenols and Temperature–Polyphenols factors on the fruity property (Figure 17D). In contrast, there was no significant difference in the interaction between Time–Temperature factors on fruity green and ripe attributes. Notably, the best factors to preserve this vital attribute as possible in EVOO cv. Picual under the high thermal processing were D-F at low frying time (3 h) and low temperature, resulting in a value of 3.1 (Figure 17A). This was followed by frying the samples at a low time (170 °C) and with added polyphenols (658.60 mg/kg), which recorded a value of 2.6 (Figure 17B). On the other hand, a low fruity score was observed when EVOO cv. Picual was fried at 6 h for 210 °C, as well as in Picual samples deep-fried at 210 °C, without the addition of polyphenol extract. Both conditions recorded a score of 0.0 (Figure 17A,C). From the radar graph results, the best conditions across the three studied factors were achieved by deep-frying Picual oil samples at 170 °C for 3 h with added polyphenols extract up to 658.60 mg/kg (Exp. 5), resulting in a fruity score of 3.5 (Figure 4A). Additionally, Exp. 1, 2, 5, and Con 2 were clustered near the fruity ripe and green attributes (Figure 4B).

There are significant differences in the interaction between Time–Temperature and Time–Polyphenols factors on the fruity property (Figure S15D). However, no significant differences were observed in the interaction between Temperature–Polyphenols factors on the fruity property (green and ripe) in EVOO cv. Cornicabra. The best combined factors to preserve this vital attribute under high thermal processing were achieved by D-F at low frying time (3 h) and high polyphenol content (658.15 mg/kg), resulting in a fruity value of 1.5 (Figure S15B). This was followed by D-F for 3 h at low frying temperature (170 °C), with a value of 1.3 (Figure S15A). Conversely, a low fruity score was observed when EVOO cv. Cornicabra was fried for 6 h at 210 °C, 6 h at 170 °C, or 6 h with or without added olive fruit extract. Similarly, oil samples deep-fried at 210 °C without added polyphenol extract recorded a fruity value of 0.0 in all cases (Figure S15A–C). Thus, D-F EVOO cv. Cornicabra at 210 °C dramatically impacts the fruity characteristics. The highest fruity value under high thermal processing was achieved at 170 °C for 3 h under D-F with a polyphenol content of 658.15 mg/kg in EVOO cv. Cornicabra sample (Exp. 5), resulting in a fruity score of 1.9 (Figure 6A). Additionally, PCA (Figure 6B) illustrated that Exp. 1, 5, 7, SOO, Con 1, and Con 2 were clustered near fruity, whereas Exp. 2, 3, 4, 6, and 8 exhibited low fruitiness (Figure 6B).

Regarding EVOO cv. Empeltre, no significant differences were observed in the interaction between Time–Temperature, Time–Polyphenols, and Temperature–Polyphenols independent variables on the fruity (green and ripe) properties (Figure S16D). The best combined conditions to preserve this vital attribute were achieved by D-F the oils at 170 °C for 3 h, frying for 3 h with added polyphenols (647.23 mg/kg), or frying at 170 °C with added polyphenols extract (650 mg/kg), all of which recorded a fruity value of 1.0 (Figure S16A–C). In contrast, other interaction factors reduced the fruity property up to 0.0 (Figure S16A–C). Additionally, D-F of EVOO cv. Empeltre at 210 °C had a dramatic negative impact on fruity characteristics. The best conditions of the three studied factors were achieved by frying the sample at 170 °C for 3 h with added polyphenol extract (Exp. 5), resulting in a fruity value of 2.0 (Figure 7A). Furthermore, PCA presented that Exp. 5, SOO, and Con 2 were clustered near the fruity green and ripeness attributes (Figure 7B). 

The findings about EVOO cv. Arbequina showed significant differences in the interaction between Time–Polyphenols and Temperature–Polyphenols factors. In contrast, there was no significant change in the combined effect of Time–Temperature factors on the fruity (green and ripe) properties (Figure S17D). The best combined conditions to preserve this vital attribute under high thermal processing were achieved by D-F the oil for 3 with added polyphenols, resulting in a fruity value of 2.7 (Figure S17B). This was followed by frying the sample at 170 °C with added olive fruit extract (2.3) (see Figure S17C). Conversely, other combined interaction factors—frying for 3–6 h and frying at 170–210 °C without added extract—reduced the fruity property up to 0.0 (Figure S17B,C). The radar graph results confirmed that the best conditions for the three studied factors were frying the sample at 170 °C for 3 h with added polyphenol extract, resulting in a fruity value of 3.3 (Exp. 5) (See Figure 8A). Additionally, PCA revealed that Con 1, Con 2, SOO, Exp. 5, 6, 7, and 8 exhibited stable fruity properties (Figure 8B).

In the case of EVOO cv. Hojiblanca, the results illustrated that there were no significant differences in the interaction between Time–Temperature, Time–Polyphenols, and Temperature–Polyphenols independent variables on the fruity (green and ripe) properties (Figure S18D). This was attributed to the low values of positive attributes such as fruitiness, in the original oil. The best combined conditions to preserve this vital attribute in EVOO cv. Hojiblanca under high thermal processing were achieved by D-F the oil at 170 °C for 3 h (Figure S18A), 6 h with added polyphenols up to 655.73 mg/kg (Figure S18B), or 170 °C with added polyphenols (Figure S18C). All three interactions recorded a fruity value of 0.9. Conversely, other interaction factors reduced the fruity property up to 0.0. Furthermore, D-F EVOO cv. Hojiblanca at 210 °C for 6 h had a dramatic negative impact on fruity characteristics. The best conditions of the three studied factors, as illustrated in the radar graph, were achieved by frying the sample at 170 °C for 3 h with added polyphenols extract, resulting in a fruity value of 1.9 (Exp. 5) (see Figure 2A). Additionally, PCA also showed that Exp. 5 and 6 exhibited the best values among all treatments, clustering near fruitiness attributes (Figure 2B).

From the obtained results, no significant differences were detected in the interaction between Time–Temperature and Time–Polyphenols variables on the fruity (green and ripe) properties in EVOO cv. Manzanilla oil. However, a significant impact was observed in the combined interaction between Temperature–Polyphenols on fruitiness (Figure S19D). The best conditions of the three studied factors were achieved by frying the sample at 210 °C for 3 h without added polyphenols (309.05 mg/kg), resulting in a fruity value of 2.0 (Exp. 3), followed by oil sample deep-fried at 170 °C for 3 h with added polyphenols (661.28 mg/kg), which recorded a fruity value of 1.7 (Exp. 5) (see Figure 9A). Additionally, PCA displayed that Exp 3 and 5 clustered near fruity attributes (Figure 9B).

The results revealed significant differences in the interaction between Time–Temperature and Temperature–Polyphenols independent variables on the fruity green property in EVOO cv. Royuela. In contrast, there was no significant alteration of the combined interaction between Time–Polyphenols (Figure S20D). The best combined conditions to preserve this vital attribute were achieved by D-F the oil at 170 °C with added OFE (3.7) (Figure S20B), followed by frying at 170 °C for 3 h (3.5) (Figure S20A). Furthermore, the best conditions of the three studied factors were achieved by frying the sample at 170 °C for 3 h with added polyphenols extract, reaching a value of 4.5 (Exp. 5). This highlights Royuela’s high fruity green value compared to other EVOO varieties, and Con 1 (original non-fried oil) showed a value of 5.9 (Figure 10A). Additionally, PCA demonstrated that Exp. 1, 5, and 6 displayed low rancidity, while other treatments clustered near rancidity (Figure 10B). One of the most interesting findings of the current study is that no values of the fruity ripe property were observed in the controls or the treatments under D-F, this might be attributed to the fact that Royuela was harvested at the green stage, giving it unique sensorial properties compared to other studied EVOO varieties in the present research.

Regarding Orujo oils, there were no significant differences in the interaction between Temperature–Polyphenols independent variables, whilst Time–Polyphenols and Time–Temperature combined interaction had a significant impact on the fruity (green and ripe) properties (Figure S21D). Notably, the best combined values were achieved by frying Orujo oil at 170 °C for 3 h or frying for 3 h without added OFE, both recording the highest fruity value (2.2) (Figure S21A,B). This was followed by frying at 3 h, with added polyphenols (652.25 mg/kg), which recorded a value of 3.1 (Figure S21B). The best conditions of the 3 studied factors, as presented in the radar graph, were achieved by frying the sample at 170 °C for 3 h with added polyphenols extract, resulting in a fruity value of 2.3 (Exp. 5) (Figure 11A). PCA showed that Exp. 2 and 4 exhibited low fruitiness, while Exp 1, 3, 5, and 7 clustered near the fruity property, along with SOO, Con 1, and Con 2 (Figure 11B).

In addition, it was observed that there are no significant differences in the interaction between Time–Temperature, Time–Polyphenols, and Temperature–Polyphenols independent variables on the fruity (green and ripe) properties in EVOO cv. Koroneiki (Figure S22D). The best combined factors interactions to preserve fruity in the fried oil were achieved by frying for 3 h with added polyphenols (663.93 mg/kg) (Figure S22B). Furthermore, radar graph findings indicated that the best conditions of the 3 studied factors were frying the sample at 170 °C for 3 h with added polyphenols, resulting in a fruity value of 2.6 (Exp. 5) (Figure 12A). Additionally, PCA showed that Exp. 5, SOO, Con 2, and Con 1 exhibited high fruitiness (Figure 12B).

No significant differences were observed in the combined interaction between Time–Temperature and Time–Polyphenols, while Temperature–Polyphenols had a notable significant impact on the fruity (green and ripe) properties in EVOO cv. Arbosana (Figure S23D). The best combined conditions to preserve this vital attribute were achieved by D-F the oil at 170 °C with added polyphenols (666.95 mg/kg) and recorded a value of 3.4 (Figure S23C). The best conditions of the three studied factors are frying the oil sample at 170 °C for 3 h with added polyphenols, resulting in a fruity value of 4.7 (Exp. 5) (Figure 13A). Moreover, PCA revealed that Exp. 1, 5, 6, 7, and 8 exhibited high bitterness (Figure 13B). Thus, the addition of exogenous polyphenol had a remarkable impact on preserving the fruitiness attribute of the deep-fried Arbosana oil.

This research also showed that there were no significant differences in the interaction between Time–Temperature and Time–Polyphenols on the fruity (green and ripe) properties in olive oil 1°. However, Temperature–Polyphenols independent variables had a significant impact (Figure S24D). The best combined conditions to preserve this vital attribute were achieved by D-F the oil at 170 °C with added polyphenols extract (653.11 mg/kg), recording a fruity value of 1.1 (Figure S24C). Furthermore, the best conditions of the three studied factors were frying the sample at 170 °C for 3 h with added OFE, resulting in a fruity value of 1.5 (Exp. 5) (Figure 3A). Additionally, PCA revealed that SOO, Con 2, Exp. 3 clustered near the fruity attribute, indicating that polyphenols played a vital role in introducing this functional property to low-quality olive oil (Figure 3B). Interestingly, according to the present data, the panelists did not detect the fruity attribute in olive oil 0.4°, likely due to the absence of this property in control oil. However, the SOO sample only exhibited fruitiness properties solely because of the added OFE (Figure 14A,B). 

### 3.4. Bitter Property

**Figure 18:**
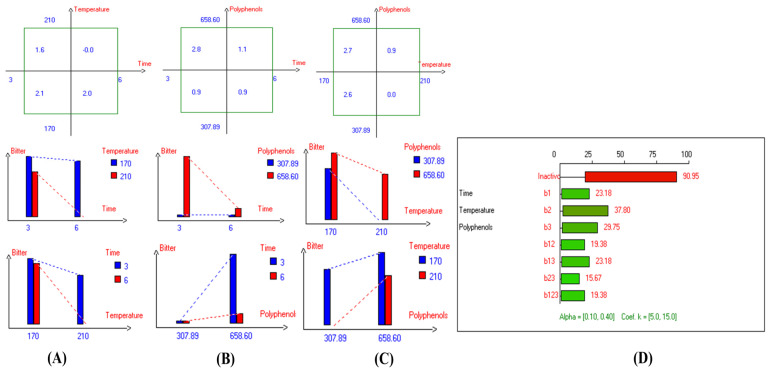
Combined interactions between the independent variables on a response variable (bitterness) in EVOO cv. Picual under D-F: (**A**) x_1_ and x_2_, (**B**) x_1_ and x_3_, (**C**) x_2_ and x_3_, and (**D**) results of variance analysis of regression equation model and the significance changes in each individual independent variable and interaction between the combined independent variables on bitter score; b represents a significant difference when be >b_123_, while b represents no significant difference when be ≤b_123_; x_1_: time, x_2_: temperature, x_3_: polyphenols.

The present research reported a significant difference in the interaction between Time–Polyphenols factors on the bitter property. Similarly, every single factor had a significant impact on bitterness in EVOO cv. Picual. However, there are no significant differences in the interaction between Time–Temperature factors and the interaction between Temperature–Polyphenols factors on bitterness (Figure 18D). The best combined factors that preserve this attribute were achieved by D-F the samples at 170 °C for 3 h, resulting in a value of 2.8 (Figure 18B), followed by frying the oil at 170 °C with added polyphenols (658.60 mg/kg), which recorded a value of 2.7. Conversely, a low bitterness score was observed when EVOO cv. Picual was deep-fried at 210 °C for 6 h, as well as in Picual samples deep-fried at 210 °C without added OFE, both of which recorded a value of 0.0 (Figure 18A,C). From the radar graph, the best conditions of the three studied factors were achieved by frying the sample at 210 °C for 3 h with added OFE, resulting in a value of 3.2. This indicates that Picual is remarkably stable at high temperatures (Figure 4A). Additionally, PCA showed that SOO, Exp. 7, and Con 1 exhibited higher bitterness (Figure 4B).

In addition, regarding the bitterness of EVOO cv. Cornicabra, it was observed that there are significant differences in the interaction between Time–Temperature factors and Time–Polyphenols factors on the bitter property (Figure S25D). However, there were no significant differences in the interaction between Temperature–Polyphenols factors. The best combined factors that preserve this attribute in Cornicabra oil were achieved by D-F at 170 °C for 3 h with the value of 1.7 (Figure S25A), followed by frying for 3 h with polyphenols addition (658.15 mg/kg), which recorded a value of 1.6 (Figure S25B). Notably, OFE provided antioxidant potential against rancidity and helped maintain a bitter score close to control. Conversely, a lower bitterness score was observed when EVOO cv. Cornicabra was deep-fried at 210 °C for 6 h or treated at 170 °C for 6 h, as well as in oil samples fried for 6 h with or without added OFE, all of which recorded a value of 0.0. Thus, prolonged high temperatures significantly reduced the bitter property (Figure S25A–C). Hence, the best conditions across the three independent variables to preserve the bitterness of fried Cornicabra were achieved by D-F at 170 °C for 3 h with added OFE (Exp. 5), maintaining the bitter property as high as possible and recording a value of 2.1 (Figure 6A). Additionally, PCA showed that SOO, Con 2, Exp. 1, 5, and 7 clustered near bitterness, while Exp. 1, 2, 3, 4, 6, and 8 exhibited low bitterness (Figure 6B).

Regarding EVOO cv. Empeltre, there were no significant differences in all combined interaction factors on the bitter property, nor in time and temperature as main individual factors. However, the only single factor that had a significant impact was polyphenol addition (Figure S26D). The best combined factors to preserve this attribute were achieved by frying the oil for 3 h with added OFE, recording a value of 3.1 (Figure S26B), followed by D-F at 170 °C with polyphenol addition, which recorded a value of 2.7 (Figure S26C). Conversely, D-F at 210 °C for 6 h, or at 170–210 °C, as well as oil fried for 3–6 h without added OFE, reduced the bitterness to zero (Figure S26A–C). Notably, OFE provided antioxidant potential against rancidity and helped maintain a bitter score close to the control. Furthermore, the best conditions to preserve this vital attribute across the three independent variables were achieved by frying Empeltre oils at 210 °C for 3 h with added exogenous polyphenols, recording a value of 3.7 (Exp. 7) (see Figure 7A). Additionally, PCA illustrated that SOO, Control 1, and Experiments 5, 6, and 7 clustered near bitterness (Figure 7B).

The results indicated that there are no significant differences in the combined interaction between Time–Temperature, Time–Polyphenols, and Temperature–Polyphenols factors on the bitter property in EVOO cv. Arbequina (Figure S27D). However, only the polyphenol individual factor had a significant effect on the bitterness. The best combined factors to preserve this attribute were achieved by frying the oil at 170 °C with added OFE recording a value of 2.4 (Figure S27C), followed by frying for 3–6 h with polyphenols addition (663.96 mg/kg), which recorded a value of 2.3 (Figure S27B,C). Thus, we can propose that Arbequina can be fried for 6 h at 170 °C without a negative impact on bitterness. Notably, polyphenols extract provides antioxidant potential against rancidity and helps maintain a bitter score close to control. Furthermore, the best conditions to preserve this vital attribute across the three independent variables were achieved by frying Arbequina oil at 170 °C for 6 h with added olive fruit extract, recording a value of 2.7 (Exp. 6) (Figure 8A). This indicates that Arbequina is a stable oil for a long frying time, thanks to OFE and the higher sensory attributes present in the control. Additionally, PCA showed that Control 1, Control 2, SOO, Experiments. 5, 6, 7, and 8 exhibited higher bitterness (Figure 8B).

The results regarding bitterness in EVOO cv. Hojiblanca indicated that there are no significant differences in the interaction between Time–Temperature factors and Time–Polyphenols factors. However, there is a significant difference in the interaction between Temperature–Polyphenols factors (Figure S28D). The best combined factors to preserve this attribute were achieved by frying the oil at 170 °C with added polyphenols, recording a value of 2.4 (Figure S28C). Notably, OFE provides antioxidant potential against rancidity and helps maintain the bitter score. The best conditions to preserve this attribute across the three independent variables were achieved by deep-frying Hojiblanca oil at 170 °C for 6 h with added polyphenols (655.73 mg/kg), recording a value of 2.5 (Exp. 6) (see Figure 2A). Additionally, PCA showed that Exp. 5 and 6 clustered near bitterness (Figure 2B).

There are no significant differences in the interaction between Time–Temperature and Time–Polyphenols variables on bitter properties in EVOO cv. Manzanilla. However, there is a significant impact observed in the combined interaction between Temperature–Polyphenols (Figure S29D). The best conditions for the three studied factors were achieved by frying the sample at 210 °C for 3 h without OFE, recording a value of 1.2 (Exp. 3), followed by deep-fried the oil sample at 170 °C for 3 h with added polyphenols (Exp. 5) with recorded a value of 1.0 (Figure 9A). Additionally, PCA showed that Exp. 3 and 5 exhibited stable bitterness compared to other treatments (Figure 9B).

Regarding EVOO cv. Royuela, the results showed that there were no significant differences in the interaction between Time–Temperature factors, Time–Polyphenols, and Temperature–Polyphenols combined factors on the bitter property (Figure S30D). The best conditions to preserve this attribute were achieved by frying the oil at 210 °C for 3 h with added OFE, recording a value of 4.5 (Experiment 6). Consequently, Royuela was a stable oil at high temperatures, thanks to the added polyphenols and unique organoleptic properties of the original Royuela oil (Figure 10A). Moreover, PCA showed that Experiments. 1, 2, 5, and 6 exhibited high bitterness along with SOO, Control 1, and Control 2 (Figure 10B).

Furthermore, there were no significant differences in the interaction between all combined factors on the bitter property in Orujo oil. This was attributed to low bitterness in the original Orujo oil due to the refining process (Figure S31D). The best conditions to preserve this attribute across the three independent variables were achieved by deep-frying Orujo oil at 170 °C for 3 h with added OFE which recorded a value of 1.7 (. 5) (Figure 11A). Additionally, PCA showed that only Exp. 5 approximately clustered near the bitter property along with SOO, Control 1, and Control 2 (Figure 11B).

In the case of EVOO cv. Koroneiki, there were significant differences in the interaction between Time–Polyphenols and Temperature–Polyphenols factors on bitter properties. However, there was no significant difference in the interaction between Time–Temperature factors (Figure S32D). The best combined factors to preserve this attribute were achieved by frying the oil at 170 °C, with added polyphenols (4.5) (Figure S32C), followed by frying the oil at 170 °C for 3 h (4.2) (Figure S32A). Remarkably, the polyphenols extract provided antioxidant potential against rancidity and helped maintain a bitter score close to control. Furthermore, the best conditions to preserve this vital attribute across three independent variables were achieved by frying Koroneiki at 170 °C for 3 h with added OFE, recording a value of 4.7 (Exp. 5) (Figure 12A). Additionally, PCA showed that Exp. 1, 3, 5, and 6 clustered near bitterness (Figure 12B).

The results indicated that there were no significant differences in the interaction between Time–Temperature factors and Temperature–Polyphenols factors on the bitter property in EVOO cv. Arbosana. However, there was a significant difference in the interaction between Time–Polyphenols factors (Figure S33D). The best combined factors to preserve this attribute were achieved by frying the oil for 6 h with added extract, recording a value of 4.1 (Figure S33B). Interestingly, OFE provided antioxidant potential against rancidity and maintained a bitter score close to control. One of the most notable findings was that Arbosana oil enriched with OFE could be fried for 6 h without affecting the bitter property. Therefore, Arbosana is a stable oil compared to other EVOOs under the same conditions. Furthermore, the best conditions to preserve this vital attribute across the three independent variables were achieved by frying the oil at 170 °C for 6 h with added olive fruit extract, recording a value of 4.3 (Exp. 6) (Figure 13A). Additionally, PCA showed that Exp. 1, 5, 6, 7, and 8 exhibited high bitterness along with SOO, Con 2, and Con 1 (Figure 13B). 

Regarding the bitterness in the deep-fried olive oil 1°, the results showed that there were no significant differences in the interaction between Temperature–Polyphenols and Time–Polyphenols. However, Time–Temperature independent variables had a significant impact (Figure S34D). The findings also showed that the best conditions to preserve this attribute were achieved by frying olive oil 1° at 170 °C with added polyphenols (653.11 mg/kg), recording a value of 2.5 (Figure S34C). Moreover, the best conditions to preserve bitterness across the three independent variables were achieved by frying olive oil 1° at 170 °C for 6 h with added OFE, recording of 2.9 (Exp 6) (Figure 3A). PCA also showed that Exp. 1, 5, 6, 7, and 8 exhibited high bitter properties along with SOO, Con 2, and Con 1 amongst all treatments (Figure 3B). 

Finally, the current data indicated that there were no significant differences in the interaction between Time–Temperature and Temperature–Polyphenols factors and factors on the bitter property in olive oil 0.4°. However, there was a significant difference in the interaction between Time–Polyphenols factors (Figure S35D). The best combined factors that conserved this functional attribute were achieved by frying the oil for 3 h with added exogenous polyphenols, recording a value of 2.2 (Figure S35B). Moreover, from the radar graph, the best conditions to preserve the bitterness were achieved by frying the samples at 170 °C for 3 h with added OFE, recording 2.3 (Exp. 5) (Figure 14A). However, the bitter score in olive oil 0.4° was non-stable at high temperatures (210 °C) for long durations (6 h) whether supplemented or not. Additionally, PCA showed that Exp. 5 and 7 clustered around bitterness (Figure 14B). Therefore, polyphenol extract provides antioxidant potential against rancidity and helps maintain a bitter score close to the control.

### 3.5. Pungent Property

**Figure 19:**
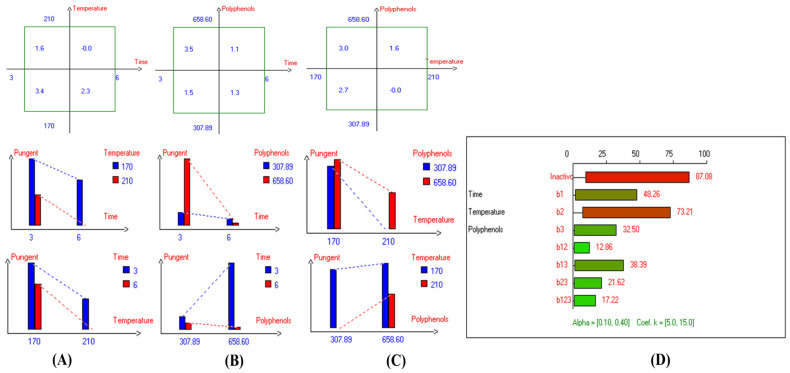
Combined interactions between the independent variables on the response variable (pungent) in EVOO cv. Picual under D-F: (**A**) x_1_ and x_2_, (**B**) x_1_ and x_3_, (**C**) x_2_ and x_3_, and (**D**) results of variance analysis of regression equation model and the significance changes in each individual independent variable and interaction between the combined independent variables on the pungent score; b represents significant a difference when be >b_123_, while b represents no significant difference when be ≤b_123_; x_1_: time, x_2_: temperature, x_3_: polyphenols.

The findings indicated that there was no significant difference in the interaction between Time–Temperature factors on the pungent sensory score in EVOO cv. Picual (Figure 19D). However, there were significant variations in the interaction between Time–Polyphenols factors and the interaction between Temperature–Polyphenols factors. A similar trend was observed for all the main individual factors, which showed a significant impact on pungent characteristics (Figure 19D). The best combined factors that kept this functional attribute were achieved through thermal processing at low frying time (3 h) with polyphenol addition, recording a value of 3.5 (Figure 19B), followed by interaction of low frying temperature (170 °C) and low frying time (3 h), with a value of 3.4 (Figure 19A). Conversely, a low pungent value was perceived when EVOO cv. Picual was fried for 6 h at 210 °C, as well as in Picual samples deep-fried at 210 °C without added OFE, both of which recorded a value of 0.0 (Figure 19A,C). The best conditions of the three studied factors were achieved by frying the oil samples at 170 °C for 3 h with added OFE (Exp. 5), recording a value of 3.9 (Figure 4A). Additionally, PCA also presented that Exp. 1, 2, 5, and Con 2 were clustered around pungent characteristics. Conversely, Exp. 3, 4, 6, and 8 exhibited low pungent properties (Figure 4B).

Additionally, the results indicated that there was no significant difference in the interaction between Temperature–Polyphenols factors on the pungent sensory score. However, there were significant variations in the interaction between Time–Temperature addition factors and the interaction between Time–Polyphenols addition factors on the pungent property in EVOO cv. Cornicabra (Figure S36D). In addition, the best combined factors that kept this property were achieved through low frying time (3 h) with polyphenols addition (658.15 mg/kg), recording a value of 2.4 (Figure S36B), followed by interaction of low frying temperature (170 °C) and low frying time (3 h), which recorded a value of 2.1 (Figure S36A). Conversely, low pungent values were observed when EVOO cv. Cornicabra was fried at 210 °C for 6 h, or at 170 °C for 6 h, as well as in oil samples fried for 6 h with or without added OFE, and in fried oil at 210 °C without added exogenous bio-phenols; all these treatments recorded a value of 0.0 (Figure S36A–C). The best condition across the three independent variables was frying the samples at 170 °C for 3 h with added OFE (Exp. 5), which maintained the pungent property as high as possible, recording a value of 3.0 (Figure 6A). Additionally, PCA showed that SOO, Con 2, Exp. 1, 5, and 7 clustered around the pungent property, while Exp. 1, 2, 3, 4, 6, and 8 exhibited low pungency (Figure 6B).

Regarding the effect of D-F on the pungent in EVOO cv. Empeltre, the results showed that there were no significant differences in all combined interaction factors, nor in time and temperature as the main individual factors. However, the only single factor that had a significant impact was polyphenol addition (Figure S37D). The best factors to maintain this attribute were achieved through low frying time (3 h) with added polyphenols (647.23 mg/kg), recording a value of 2.9 (Figure S37B), followed by the interaction of low frying temperature (170 °C) with added OFE, which recorded a value of 2.8 (Figure S37C). On the other hand, low pungent values were observed when EVOO cv. Empeltre was fried at 210 °C for 6 h, or in those fried at 170–210 °C, or in oils fried for 3–6 h without OFE; all these treatments recorded a value of 0.0 (Figure S37A–C). Thus, OFE plays a positive role in preserving pungency in EVOO cv. Empeltre under high thermal processing. Moreover, the best conditions to preserve the pungent attribute, as illustrated in the radar graph for the three independent variables, were achieved by frying the samples at 210 °C for 3 h with added polyphenols, recording a value of 3.2 (Exp. 7) (see Figure 7A). Consequently, Empeltre exhibited high stability at high temperatures, thanks to the added OFE. Additionally, PCA reported that SOO, Con 1, Exp. 5, 6, and 7 were clustered around pungency (Figure 7B).

Moreover, the findings indicated that there were significant differences in the combined interaction between Time–Temperature, Time–Polyphenols, and Temperature–Polyphenols factors on the pungent score in EVOO cv. Arbequina (Figure S38D). The best factors to maintain this characteristic were achieved by frying the oil samples at 170 °C without added OFE, recording 5.0 (Figure S38C), followed by frying for 6 h without added extract with a value of 4.8 (Figure S38B). Conversely, a low pungent value was observed when EVOO cv. Arbequina was fried at 170 °C with OFE, reaching a value of 2.7 (Figure S38C). Moreover, the best conditions to preserve the pungent attribute across the three independent variables are achieved by frying Arbequina at 170 °C for 6 h without added OFE, recording a value 5.6 (Exp. 2) (Figure 8A). Consequently, Arbequina is a stable oil during long continuous D-F time, thanks to the positive sensorial attributes of the original oil. Additionally, PCA showed that Exp. 1, 2, 3, and 4 clustered around pungency (Figure 8B).

For EVOO cv. Hojiblanca, the findings reported that there was a significant difference in all combined interactions under study on the pungent sensory score (Figure S39D). The best factors to maintain this attribute were achieved through D-F at 170 °C for 3 h, recording a value of 2.2 (Figure S39A). Additionally, as illustrated in the radar graph, the best conditions to preserve the important attribute across the three studied independent variables are achieved by frying Hojiblanca oil at 170 °C for 3 h with or without added polyphenols, both of which recorded a value of 2.2 (Exp. 1 and 5) (Figure 2A). Consequently, Hojiblanca was an unstable oil at high temperatures for long durations, even if supplemented with exogenous polyphenols, due to the low positive attribute in the original oil. Thus, olive oil variety is a critical criterion to preserve the positive sensorial attributes after exposure to high thermal processing. Moreover, PCA showed that Exp. 2, 4, 7, and 8 exhibited low pungency (Figure 2B).

In the case of EVOO cv. Manzanilla, it was observed that there are no significant differences in the interaction between all independent variables on pungent property. Thus, Manzanilla oil was a stable edible oil under D-F, thanks to the unique organoleptic properties of the original oil (Figure S40D). Further, the best conditions for the three studied factors were achieved by frying the samples at 210 °C for 3 h without OFE (Exp. 3), recording a value of 4.3 (although the panelists attributed this high value to the presence of rancid compounds) (Figure 9A). In addition, PCA showed that Exp. 1, 2, 3, and 5 exhibited close distribution distance around the pungent property (Figure 9B).

Additionally, the current findings indicated that there were significant differences in the combined studied interactions between the independent variables on the pungent sensory score of EVOO cv. Royuela (Figure S41D). The best factors to maintain this attribute were achieved through deep-frying at 170 °C for 3 h (4.5) (Figure S41A), followed by frying at 170 °C with polyphenols incorporation (4.3) (Figure S41C). Moreover, the best conditions to conserve the pungent attribute were achieved by frying the oil samples (170 °C/3 h) with or without added polyphenols, recording a value of 4.7 (Exp. 1) and 4.4 (Exp. 5, respectively) (Figure 10A). Thus, pungent characteristics were stable at low temperatures and short frying times. Moreover, PCA also illustrated that Exp. 1, 5, and 6 exhibited high pungency along with SOO, Con 1, and Con 2 (Figure 10B).

Interestingly, the panelists did not detect the pungent property in Orujo oil, either in the controls or in the deep-fried oils, due to the absence of this property in the original oil.

Furthermore, the results indicated that there were significant differences in the interaction between factors and Time–Polyphenols and Temperature–Polyphenols factors on pungent properties in EVOO cv. Koroneiki. However, there was no significant difference in the interaction between Time–Temperature factors (Figure S42D). The best combined factors to preserve this functional attribute were achieved by frying the oil for 3 h without adding polyphenols (4.0) (Figure S42B), followed by frying the oil at 210 °C for 3 h (3.6) (Figure S42A). Moreover, the best conditions to preserve the pungent attribute across the three independent variables were achieved by frying Koroneiki at 210 °C for 3 h without added olive fruit extract, recording a value of 4.4 (Exp. 3) (Figure 12A). This was attributed to the unique sensorial attributes of original, non-supplemented Koroneiki oil. Additionally, PCA showed that Exp. 1, 3, 5, and 6 clustered around the pungent characteristic (Figure 12B).

Regarding EVOO cv. Arbosana, the findings indicated that there were no significant differences in the interaction between all combined independent variables on the pungent property (Figure S43D). Thus, Arbosana oil exhibited high stability of pungent properties under high thermal processing owing to the high pungent score in the original oil. Moreover, the best conditions to preserve the vital pungent attribute across the three independent variables were achieved by frying Arbosana at 170 °C/3 h with or without added polyphenols, recording a value of 3.9 (Exp 1) and 3.6 (Exp 5, respectively) (Figure 13A). Additionally, PCA indicated that Exp. 1, 5, 6, 7, and 8 exhibited high pungent properties along with SOO, Con 2, and Con 1 (Figure 13B).

Furthermore, the current findings revealed that there are significant differences in the interaction between all combined factors on the pungent sensory score in olive oil 1°(Figure S44D). The best combined factors to maintain this functional attribute were achieved through low frying time (3 h) with polyphenol addition, recording a value of 2.7 (Figure S44B), followed by the interaction of low frying temperature (170 °C) with added OFE, which recorded a value of 2.5 (Figure S44C). Additionally, the best conditions to preserve the pungent attribute are achieved by frying olive oil 1° at 170 °C for 3 h with added OFE, recording a value of 3.2 (Exp. 5) (see Figure 3A). Moreover, PCA showed that Exp. 5 exhibited high pungent properties (Figure 3B). Accordingly, the pungent property in olive oil 1° was stable under these conditions. However, at high temperatures for a duration without added OFE, the pungent would decrease.

Finally, from the data obtained, the panelists did not detect a pungency attribute in olive oil 0.4°, due to the absence of this property in the original oil (control oil) (Figure 14A,B).

## 4. Discussion

Sensory properties of olive oils are classified as positive or negative. The positive attributes define the flavor of a certain olive oil, creating a balance of green, fruity, bitter, and pungent sensory notes [25]. Sensory scores vary primarily based on the volatile and non-volatile minor constituents. Volatile minor compounds, mainly resulting from the oxidation/rancidity of the fatty acids, stimulate the olfactory receptors. Furthermore, sensory defects in olive oils are primarily associated with the volatile profile, whether caused by chemical or enzymatic rancidity. For example, chemical oxidation generates off-flavors such as hept-2-enal and pent-2-enal. These volatile off-flavor compounds are responsible for objectionable flavors, including fusty/muddy sediments, musty, winey/vinegary/sour, and rancidity [26]. In contrast, non-volatile minor substances, such as polyphenols stimulate the taste receptors, enhancing sensitivity to astringency, pungency, bitterness, and metallic features. This combined effect of taste, odor (aroma), and chemical interactions, is perceived as the flavor of olive oils [27].

The current research evaluated 19 different sensorial descriptors of different olive oils in comparison to sunflower oils under several D-F conditions. Fifteen negative sensory attributes were assessed, including fusty/muddy sediment, musty/humid/earthy, winey/vinegary/acid/sour, frostbitten olives (wet wood), and rancid. Additionally, the panelists evaluated other negative attributes, such as metallic, dry hay, grubby, rough, brine, heated or burnt, vegetable water, esparto, cucumber, and greasy. Finally, four positive attributes were assessed, including, green fruity, ripe fruity, bitter, and pungency. Among the 19 sensory descriptors evaluated, only 8 descriptors (4 defects and 4 positive markers) namely, fusty/muddy sediment, winey/vinegary/acid/sour, frostbitten olives (wet wood), rancid, fruity (green), fruity (ripe), bitter, and pungent were successfully developed to characterize the sensory quality of various olive oil categories under D-F. Consequently, musty/humid/earthy, metallic, dry hay, grubby, rough, brine, heated or burnt, vegetable water, esparto, cucumber, and greasy were not perceived by the panelists in either olive oil categories or sunflower oils both fried and not.

Regarding the fusty/muddy sediment score, most of the studied twelve olive oil categories and the two sunflower categories did not exhibit fusty/muddy sediment defects. On the other hand, only EVOO cv. Cornicabra (control), olive oil 1° (control), and some treatments from olive oil 1°s under D-F recorded these defects. Fusty is the characteristic flavor of oils obtained from olives in an advanced stage of the fermentation process. The results also indicated that olive oil 1° is the least stable oil against these defects under D-F conditions, due to the low sensory qualities of the original oil compared to EVOO varieties. These defects are primarily attributed to poor practices during processing, inadequate fruit preservation prior to olive oil processing, and fermentation of olives stored in piles while awaiting pressing. Additionally, fusty defects are caused by enzymatic activities before oil extraction or alterations during olive oil storage [28]. Hence, Neugebauer et al. [26] reported that microbial spoilage of olive fruits is among the most common causes of two types of off-flavors in olive oils, identified as musty and fusty/muddy sediment. Thus, fusty markers could serve as a promising indicator for low-quality olive oils. The present research confirmed that D-F does not produce a fusty score if the original oil is fusty-free and can exhibit this defect if the original oil already contains it. 

In addition, concerning winey/vinegary/acid/sour; the results indicated that olive oil 1° (control) and some treatments of olive oil 1° under D-F exhibited these defects. Moreover, among all studied EVOO types, only Con 1 and Con 2 of Hojiblanca oils were found to have winey/vinegary/acid/sour defects. These defects can be attributed to the high concentration of acetic acid, ethyl acetate, and ethanol, as well as to inadequate fruit preservation before olive oil processing [29,30].

Observing frostbitten olives (wet wood) defects, among all the studied oils, only olive oil 1° (control) and some treatments of olive oil 1° under D-F exhibited these defects. The frostbitten olives or wet wood sensory defect is primarily caused by the freezing of olives while still on the tree [31]. This defect is also occasionally detected in virgin olive oil and EVOO blended with lower-quality olive oils [32]. EVOO may be blended with cheaper olive oils or refined olive oils (e.g., olive oil 1°), which are of lower quality than EVOO. Thus, wet wood defects can inadvertently arise due to inappropriate production practices and storage conditions [33].

Moreover, rancidity defect is one of the most critical sensory markers and is considered a key indicator of the oxidation/rancidity progress during the storage and/or D-F. During frying, olive oils can deteriorate and develop rancidity due to autoxidation and hydrolytic alterations. These changes are influenced by the fatty acid profile and endogenous minor antioxidants [34]. Additionally, rancid oil results from oil oxidation, characterized by the presence of several aldehydes and the absence of alcohols, C6 aldehydes, and esters [29]. Furthermore, oil oxidation/rancidity is commonly assessed by the formation of off-flavors, attributed to compounds such as nonanal and hexanal [35].

**Figure 20:**
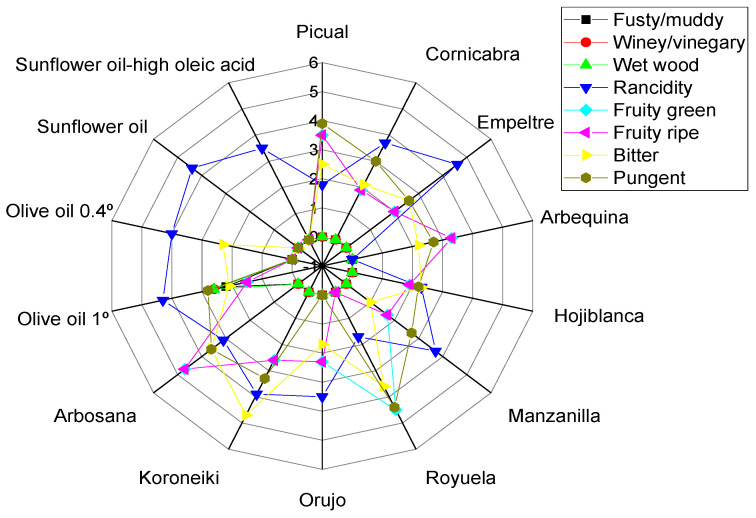
A summary of the best D-F conditions of the mean sensorial markers of several olive oil brands compared to sunflower oil and sunflower oil-high oleic acid. Exp. 5 was selected as the best treatment for several olive oil categories, while Exp. 3 was chosen for the sunflower oil categories.

The current data indicated that there are variations among all studied olive oil categories and sunflower types in perceiving rancidity. Moreover, the results confirmed that the lowest rancid values, along with higher positive sensorial attributes for most of the studied olive oils, were observed in oils deep-fried at 170 °C for 3 h with added polyphenols (see Table 2 and Figure 20). EVOOs under these conditions, such as Arbequina, Royuela, Picual, Hojiblanca, and Arbosana oils, exhibited low rancidity (≤3.5) among all oils. Therefore, the present study suggested that EVOO quality can be reduced to the following lowest quality of olive oil (VOO grade), which exhibits some negative sensory defects (≤3.5) [27]. Thus, the interaction between time and temperature alone has a greater negative impact on the sensory properties of EVOO in terms of high rancidity. On the other hand, the presence of the exogenous polyphenol factor resulted in a three-factor interaction with low rancidity values. Interestingly, the addition of exogenous polyphenols helped stabilize the oil under high temperatures, and these findings align with those reported in recent reports. For instance, Lozano-Castellón et al. [36] stated that minor constituents, particularly phenolic compounds, prevent fatty acid and vitamin oxidation during cooking, inhibit the formation of undesired compounds (e.g., acrylamide), and reduce degradation of EVOOs during cooking compared to other vegetable oils, thanks to their polyphenolic profile. Moreover, foodstuffs enriched with EVOO antioxidants are less prone to oxidation and to the formation of undesirable compounds, such as off-flavors. In addition, the present study confirmed that EVOOs have a low rancidity score compared to refined edible oils such as olive oil 0.4°, olive oil 1°, and sunflower oil. A recent study also reported that EVOO is a highly attractive medium for short-term deep-frying of French fries compared to refined oils like soybean oil and sunflower oil, due to its high oxidative stability, preservation of unsaturated fats, and low formation of free fatty acids and carbonyl compounds [37].

While the positive sensory attributes are appealing, the negative ones, like rancidity, play a decisive role in determining labeling. If sensory defects are detected, olive oil cannot be labeled as EVOO when the median defect exceeds 3.5 [38]. As a result, the above-mentioned EVOO varieties can be reclassified as VOO even under D-F at 170 °C/3 h/with added polyphenols (see Table 2 and Figure 20). These varieties could be utilized for D-F purposes at both the industrial level and for consumer practices, primarily due to their low defects. In summary, VOO-rich exogenous antioxidants from olive fruit extract are recommended for continuous D-F purposes for up to 3 h due to their well-documented nutritional benefits, uniquely stable positive sensory attributes, low rancidity, and enhanced oil stability.

Additionally, low rancidity values were achieved at 210 °C for 3 h under D-F with added polyphenols in EVOO cv. Cornicabra, recording a rancidity score of 3.1. Thus, Cornicabra is stable at high frying temperatures compared to other EVOOs, and therefore can also be reclassified as VOO even after D-F. This may be attributed to the unique organoleptic properties of EVOO cv. Cornicabra, which protect it from oxidation. Moreover, the Cornicabra cultivar is characterized by medium to intense levels of its most prominent sensory descriptors, such as bitterness and pungency [31].

One of the most interesting results in this research is that EVOO cv. Arbequina emerged as the best option among the studied varieties to obtain oil without rancidity under D-F conditions. Thus, deep-fried Arbequina oil remains classified as EVOO due to zero median defects and maintains maximum quality under low time/low temperature/with added polyphenols (Table 2 and Figure 20). This stability can be attributed to the cultivar and the exogenous phenolic content, which enhanced oxidative stability. A recent study by Borges et al. [39] also reported that the composition and antioxidant properties of the EVOO are strongly influenced by cultivars and, within cultivars, can also vary depending on the harvest year and the crop stage. On the other hand, D-F EVOO cv. Arbequina without added polyphenols exhibited a dramatically negative impact on fruity characteristics. Therefore, natural extract is of great importance to preserve this important property under D-F conditions. 

The present results also indicated that Hojiblanca is an unstable oil under high temperatures for a long time. In contrary, Orujo demonstrated high stability at high temperatures of D-F, but only when supplemented with OFE. In addition, olive oil 1° was found to be a stable oil under D-F at high temperature (210 °C) with added OFE. On the other hand, without exogenous polyphenols, olive oil 1° recorded the highest rancid value among all studied oils. Furthermore, olive oil rancidity is not a fixed characteristic; it depends on the antioxidant content, variety, fruit ripening stage, climate change, agroclimatic conditions, and olive growing techniques [40].

Moreover, according to the present research, SOHO demonstrated low rancidity under D-F and proved to be more stable than SO. In the European market, refined sunflower oil is known for its relatively low oxidative stability. This makes it suitable for cold applications such as salad oil or for short-term D-F, stewing, or baking. In the food industry, it is commonly used to produce margarines and cooking fats or for manufacturing mayonnaise. In contrast, HOSO (typically +80% oleic acid), is distinguished by its very high stability and is, consequently, well suited for long-term industrial D-F applications [41].

In summary, the present research indicated that OFE effectively inhibited and/or reduced oxidation. This effect is primarily attributed to the antioxidant potential of the natural olive fruit extract and its polyphenols e.g., HTyr [42,43]. Similarly, another study reported that natural antioxidants from natural sources, such as lyophilized pomegranate, orange, and beetroot leaf extracts, exhibited significant antioxidant potential and enhanced the stability of the soybean oil during the D-F [44]. Additionally, Oubannin et al. [45] reported that natural antioxidants can prevent oxidative degradation in edible oils enriched with natural antioxidants, thus preserving their quality. Likewise, a mixture of strawberry, ginger, cinnamon, and beetroot (1:1:1:1) proved to be effective in inhibiting the oxidation/rancidity of virgin coconut oil (VCO) with a concentration of 1500 ppm [46].

Regarding fruitiness, the fruity property is a crucial attribute that distinguishes the high-quality EVOO brand and allows us to classify the oils into various quality and sensorial grades. The organoleptic properties related to the fruity flavor of this valued product are attributed to specific compounds in its composition, such as carbonyl compounds, volatile compounds, and phenolic compounds [47].

Additionally, one of the most interesting findings of the current study was that EVOO cv. Royuela retained its fruity attributes completely. There were no values of the fruity ripe property in controls or treatments under D-F. This could be attributed to Royuela being harvested at the green stage, which imparts unique sensory properties compared to other studied EVOO varieties (Table 2 and Figure 20). Therefore, Royuela exhibited high stability, and there was a high possibility that fruity green might prevent oxidation and off-flavor formation. Indeed, recent studies have shown that olive oil cultivars harvested at the green stage possess higher polyphenolic content, β-sitosterol, and antioxidant activity than those harvested at the fully mature stage [48,49].

Furthermore, intensive D-F conditions at 210 °C for 6 h dramatically had a dramatic negative impact on the fruity characteristics of some investigated oils e.g., EVOO cv. Hojiblanca. Indeed, olive variety and ripeness stage (green, green/ripe, or fully ripe) play a crucial role in the oxidative stability of EVOOs [50]. In general, green fruity aspects are often preferred over full ripe EVOO for D-F purposes, as antioxidants and related parameters decrease as olive fruit ripens. Additionally, pigment content also declines during ripening progress [51]. 

Overall, this research stated that EVOO exhibited high fruity attributes, either fruity green and ripe or fruity green alone. However, mixed EVOO with VOO or refined VOO displayed reduced fruity properties. A similar trend was reported in a recent study, which confirmed that VOO showed only slight intensity in some sensory positive attributes [52]. The present study also indicated that olive fruit extract positively influenced EVOOs, either by enhancing positive attributes or preserving existing ones during D-F. 

Regarding bitterness attributes, the bitter property is one of the most essential characteristics that distinguish the high-quality EVOO. In fact, bitterness is closely associated with phenolic compounds [53]. Notably, the presence of pent-1-en-3-one is positively correlated with bitterness and pungent attributes, while hexanal shows a negative correlation with these attributes. Furthermore, Z-Hex-3-en-1-ol and E-hex-2-enal are negatively correlated with bitterness and pungency, respectively [54].

Notably, the present study confirmed that the addition of polyphenol extract enhances antioxidant potential against rancidity and helps maintain bitter scores close to control in EVOOs, such as Picual, Koroneiki, Empeltre, and Arbosana. Thus, EVOO variety plays a crucial role in bitterness properties, due to the variance observed among EVOOs perceiving bitter scores. Recent studies have also shown that phenolic compounds contribute significantly to several important organoleptic properties, such as bitterness, color, and astringency [55,56].

One of the most intriguing findings of this study is that Empeltre oil can be deep-fried without negatively affecting its bitterness for up to 3 h at a high temperature (210 °C) when supplemented with olive fruit extract. Consequently, Empeltre demonstrates high stability at elevated temperatures, thanks to the added OFE and/or original strong positive sensory attributes. Therefore, selecting an EVOO cultivar with naturally high bitterness could be advantageous for producing stable fried oil. In addition, Arbequina oil supplemented with exogenous natural polyphenols showed higher bitterness during D-F under prolonged frying conditions (170 ºC/6 h), thanks to the added olive oil extract. Furthermore, a recent study confirmed that hydroxytyrosol, tyrosyl, and their derivatives are key contributors to the sensory qualities of EVOO, including bitterness [57].

The current research also found that Orujo oil exhibited low bitterness, primarily due to the refining process, which reduced this vital property. As a result, the bitterness hierarchy observed was as follows: EVOO > mixed EVOO and VOO > VOO > refined olive oil. However, added polyphenol enhanced bitterness properties in low-quality olive oils. Thus, polyphenol extracts demonstrate antioxidant potential against rancidity and help maintain bitterness scores close to control. On the other hand, the bitter attribute in olive oil 0.4° was found to be unstable at high temperatures for extended periods, whether supplemented or not. Consequently, olive oil cultivars and brand categories should be prioritized when considering their suitability for D-F purposes.

Notably, polyphenol extracts not only reduce rancidity but also maintain bitterness scores. One of the most intriguing findings was that Koroneiki and Arbosana oils can be deep-fried for 6 h at 170 °C, with added OFE, without negatively affecting bitterness. Therefore, bitterness can persist under thermal process due to the transformation of polyphenols into aldehydic forms, as previously reported by Mateos et al. [58], who confirmed that at high D-F temperatures, bitterness strongly correlates with the aldehydic form of oleuropein aglycone.

Regarding pungency properties, pungency is one of the most essential attributes for identifying high-quality EVOO brands and is closely related to minor bioactive substances. Both pungency and bitterness scores in olive oil samples were highly correlated with their total phenolic compounds and volatile compounds [59]. Moreover, Ferrer-Gallego et al. [60] reported that polyphenol-rich olive oils contribute to higher bitter and pungent sensory scores.

The current results showed that there are variations among olive oil categories in perceiving pungency and in the stability of this functional attribute. For instance, under ideal conditions, namely Exp 5 (D-F for 6 h at 170 °C, with added polyphenols), it was observed that Picual, Cornicabra, Royuela, Arbosana, Koroneiki, Arbequina, and Manzanilla samples exhibited remarkably stable and intensive pungency among deep-fried EVOOs. These oils could be promising candidates for deep-frying applications, not only at the industrial level but also for home practices. On the other hand, Hojiblanca proved to be unstable at high temperatures and long time even with polyphenol supplementation, due to the low positive attributes in the original oil. Similar trends were observed in olive oil 0.4° and Orujo oil, which showed low pungency values under the best conditions. Additionally, the pungent property in olive oil 1° was stable at low frying time and low temperature with added polyphenols, However, at high temperatures for prolonged periods without supplementation, pungency declined. Consequently, olive oil variety is a critical factor in preserving positive sensory attributes, such as pungency after exposure to high thermal processing. Carmona et al. [61] studied the effect of D-F (190 °C/2 h) on the stability of different olive oil types (EVOO, pomace olive oil, and ROO). Using Raman spectra, they reported that EVOO was the most stable type compared to other olive oils, as indicated by its lowest intensity value at 3008 cm^−1^. Thus, the oxidative stability of EVOO may be linked to its minor bioactive compounds and tocopherol ratio.

## 5. Conclusions

High thermal processing induces sensorial changes in deep-fried EVOOs. Continuous deep-frying of olive oils affects their unique sensory attributes. Therefore, adjusting D-F conditions and selecting the best frying olive oils are essential to preserve unique sensory aspects and achieve highly stable oils. From the results obtained in the present research, significant differences were observed in all the sensory parameters assessed between the supplemented fried olive oil categories and non-supplemented ones mainly in rancidity and positive sensorial attributes. Moreover, the addition of the OFE enhanced sensory attributes, including fruity, bitterness, and pungency of the most deep-fried EVOOs. Based on these findings, natural OFE can be incorporated into EVOO to achieve higher sensory scores, in particular, lower median defects (e.g., rancidity) combined with high and stable positive sensory attributes.

Furthermore, most EVOOs remained thermally stable when fried at 170 °C for 3 h. However, they degraded and exhibited higher median defects, particularly rancidity at 210 °C, especially after 6 h. Additionally, the present study confirmed that D-F significantly reduced the intensity of fruity ripe, bitter, pungent, and fruity green characteristics, while increasing rancidity. Nevertheless, exogenous phenolic compounds enhanced the oxidative stability of the deep-fried oil, particularly at temperatures above 170 °C for extended periods. Moreover, variation in polyphenol concentration among original EVOOs and other olive oil types was observed depending on the cultivar. Royuella and Arbosana revealed higher polyphenol content, while Orujo recorded the lowest. Thus, a high initial polyphenolic content, not only protected the oil from rancidity but also contributed to preserving some desired sensory attributes. In summary, although some desired sensory properties of EVOOs may decrease after high thermal processing, they can be retained in the deep-fried EVOOs, thanks to natural exogenous polyphenols from OFE enriched with hydroxytyrosol and its derivatives. The present research confirmed that OFE can be used to provide stable EVOOs with enhanced positive sensory qualities and fewer defects. It also represents a natural antioxidant and a promising strategy for the D-F process with EVOOs, not only for domestic use but also on an industrial scale.

## Figures and Tables

**Table 1:** Mean total polyphenolic content (±SD) of the investigated oils by HPLC.

Oil Category	Original Oil	Supplemented Oil	Supplemented Oil and Original Oil Mix
Picual	307.89 ± 8.76	1524.33 ± 2.43	658.60 ± 4.46
Cornicabra	275.67 ± 9.37	1683.03 ± 32.09	658.15 ± 17.44
Empeltre	337.92 ± 11.58	1468.88 ± 47.30	647.23 ± 5.26
Arbequina	227.71 ± 6.32	1384.96 ± 31.46	663.96 ± 6.53
Hojiblanca	209.51 ± 9.19	1208.67 ± 41.67	655.73 ± 13.09
Manzanilla	309.05 ± 16.69	1255.28 ± 28.17	661.28 ± 7.78
Royuela	400.63 ± 10.17	1211.16 ± 30.83	662.38 ± 10.84
Orujo	3.89 ± 0.29	1259.06 ± 20.40	652.25 ± 5.55
Koroneiki	327.77 ± 12.99	1230.89 ± 18.22	663.93 ± 3.15
Arbosana	393.00 ± 6.59	1434.58 ± 27.86	666.95 ± 16.55
Olive oil 1°	181.88 ± 7.21	1152.24 ± 45.18	653.11 ± 17.59
Olive oil 0.4°	26.50 ± 2.40	1113.40 ± 14.77	650.87 ± 13.12
Sunflower oil *	0	-	-
Sunflower oil-high oleic acid *	0	-	-

*: non-applicable for the supplementation process with polyphenols.

**Table 2:** A summary of the best * D-F conditions of the mean sensorial markers of several olive oil brands compared to sunflower oil and sunflower oil-high oleic acid.

Vegetable Oil	Fusty/Muddy	Winey/ Vinegary	Wet Wood	Rancidity	Fruity Green	Fruity Ripe	Bitter	Pungent
EVOO cv. Picual	0.0	0.0	0.0	1.8	3.5	3.5	2.5	3.9
EVOO cv. Cornicabra	0.0	0.0	0.0	3.7	1.9	1.9	2.1	3.0
EVOO cv. Empeltre	0.0	0.0	0.0	4.6	2.0	2.0	2.6	2.6
EVOO cv. Arbequina	0.0	0.0	0.0	0.0	3.3	3.3	2.2	2.7
EVOO cv. Hojiblanca	0.0	0.0	0.0	2.3	1.9	1.9	2.3	2.2
EVOO cv. Manzanilla	0.0	0.0	0.0	3.7	1.7	1.7	1.0	2.7
EVOO cv. Royuela	0.0	0.0	0.0	1.7	4.5	0.0	3.6	4.4
Orujo olive oil	0.0	0.0	0.0	3.5	2.3	2.3	1.7	0.0
EVOO cv. Koroneiki	0.0	0.0	0.0	3.9	2.6	2.6	4.7	3.3
EVOO cv. Arbosana	0.0	0.0	0.0	3.1	4.7	4.7	3.6	3.6
Olive oil 1°	2.2	2.6	2.6	4.3	1.5	1.5	2.1	2.8
Olive oil 0.4°	0.0	0.0	0.0	4.0	0.0	0.0	2.3	0.0
Sunflower oil	0.0	0.0	0.0	4.4	0.0	0.0	0.0	0.0
Sunflower oil—high oleic acid	0.0	0.0	0.0	3.5	0.0	0.0	0.0	0.0

* Exp. 5 was selected as the best treatment for several olive oil categories while Exp. 3 was chosen for the sunflower oil categories.

## Data Availability

The original contributions presented in the study are included in the article/Supplementary Material, further inquiries can be directed to the corresponding author.

## Supplementary Materials

The following supporting information can be downloaded at https://www.mdpi.com/article/10.3390/foods13233953/s1, Table S1. Experimental design (2^3^) methodology of olive oils supplemented with olive fruit extract under different D-F conditions. Table S2. Experimental design (2^2^) methodology of sunflower oils under different D-F conditions. Tables S3–S16: Each Table illustrates the mathematical equation that predicts the relationship between the independent variables and the response variables (sensory scores) of each investigated oil category under D-F. Each corresponding title for each Table was provided in detailed captions in the Supplementary Materials file of this article. Figures S1–S44: Each Figure illustrates the results that show the effect of the independent variables on each response variable (sensory properties) of each investigated oil category under D-F conditions, as well as the results of variance analysis of regression equation model and the significance changes in each individual independent variable and interaction between the combined independent variables on each sensory score. Each corresponding title for each Figure was provided in detailed captions in the Supplementary Materials file of this article.
