# Supplementary material for: Evaluation of Sensorial Markers in Deep-Fried Extra Virgin Olive Oils: First Report on the Role of Hydroxytyrosol and Its Derivatives
Source: Foods. 2024 Dec 7;13(23):3953. doi: 10.3390/foods13233953 (PMC11641459; doi:10.3390/foods13233953)
Supplement: Supplementary file 1 [file foods-13-03953-s001.zip › foods-3324905-supplementary.pdf]

Supplementary Materials

Article

Evaluation of Sensorial Markers in Deep-Fried Extra Virgin Olive Oils: First Report on the Role of Hydroxytyrosol and Its Derivatives

Taha Mehany, José M. González-Sáiz, Jorge Martínez and Consuelo Pizarro *

Department of Chemistry, University of La Rioja, 26006 Logroño, Spain; taha.abdellatif@unirioja.es (T.M.); josemaria.gonzalez@unirioja.es (J.M.G.-S.); jorge.martinez@unirioja.es (J.M.)

* Correspondence: consuelo.pizarro@unirioja.es; Tel.: +34-941299626

Table S1. Experimental design (2^3) methodology of olive oils supplemented with olive fruit extract under different deep-frying conditions.

Experiment	Design Matrix			Independent Variables			Response
	X_1	X_2	X_3	Time (h)	Temp. (°C)	Polyphenols (mg/kg)	
1	-1	-1	-1	3	170	-	Sensory descriptors
2	+1	-1	-1	6	170	-	
3	-1	+1	-1	3	210	-	
4	+1	+1	-1	6	210	-	
5	-1	-1	+1	3	170	650	
6	+1	-1	+1	6	170	650	
7	-1	+1	+1	3	210	650	
8	+1	+1	+1	6	210	650	

Level	Time (h)	Temp. (°C)	Polyphenols (mg/kg)
-1	3	170	Original concentration (0 addition)
+1	6	210	650

Table S2. Experimental design (2^2) methodology of sunflower oils under different deep-frying conditions.

Experiment	X_1	X_2	Independent Variables		Response
			Time (h)	temperature (°C)	
1	-1	-1	3	170	Sensory descriptors
2	+1	-1	6	170	
3	-1	+1	3	210	
4	+1	+1	6	210	

Level	Time (h)	Temp. (°C)
-1	3	170
+1	6	210

Table S3. Mathematical equation that predicts the relationship between the independent variables (time, temperature, and polyphenols addition) and the response variables of EVOO cv. Picual under deep-frying.

Response (dependent variables)	Equation (predicted value)
Fusty/muddy sediment	$Y_1=0.00$
Winey/vinegary/acid/sour	$Y_2=0.00$
Frostbitten olives	$Y_3=0.00$
Rancidity	$Y_4=4.74+1.41x_1+1.09x_2-0.31x_3+0.36x_1x_2+0.86x_1x_3-0.46x_2x_3-0.09x_1x_2x_3$
Fruity green	$Y_5=1.58-0.45x_1+1.13x_2-0.25x_3+0.00x_1x_2-0.38x_1x_3-0.20x_2x_3-0.08x_1x_2x_3$
Fruity ripe	$Y_6=1.58-0.45x_1+1.13x_2-0.25x_3+0.00x_1x_2-0.38x_1x_3-0.20x_2x_3-0.08x_1x_2x_3$
Bitter	$Y_7=1.44-0.44x_1-0.64x_2-0.54x_3-0.36x_1x_2-0.44x_1x_3+0.26x_2x_3-0.36x_1x_2x_3$
Pungent	$Y_8=1.85-0.67x_1-1.05x_2+0.47x_3-0.12x_1x_2-0.55x_1x_3+0.32x_2x_3-0.25x_1x_2x_3$

where x_1= Time; x_2= temperature; x_3= Polyphenols supplementation.

Table S4. Mathematical equation that predicts the relationship between the independent variables (time, temperature, and polyphenols addition) and the response variables of EVOO cv. Cornicabra under deep-frying.

Response (dependent variables)	Equation (predicted value)
Fusty/muddy sediment	$Y_1=0.00$
Winey/vinegary/acid/sour	$Y_2=0.00$
Frostbitten olives	$Y_3=0.00$
Rancidity	$Y_1=5.36+1.34x_1+0.71x_2-0.21x_3+0.14x_1x_2+0.41x_1x_30.66x_2x_3+0.21x_1x_2x_3$
Fruity green	$Y_2=0.48-0.48x_1-0.20x_2+0.28x_3+0.20x_1x_2-0.28x_1x_3+0.00x_2x_3-0.00x_1x_2x_3$
Fruity ripe	$Y_3=0.48-0.48x_1-0.20x_2+0.28x_3+0.20x_1x_2-0.28x_1x_3+0.00x_2x_3-0.00x_1x_2x_3$
Bitter	$Y_4=0.58-0.58x_1-0.28x_2+0.25x_3+0.28x_1x_2-0.25x_1x_3+0.05x_2x_3-0.05x_1x_2x_3$
Pungent	$Y_5=0.76-0.76x_1-0.31x_2-0.44x_3+0.31x_1x_2-0.44x_1x_3+0.01x_2x_3-0.01x_1x_2x_3$

where x_1= Time; x_2= temperature; x_3= Polyphenols supplementation.

Table S5. Mathematical equation that predicts the relationship between the independent variables (time, temperature, and polyphenols addition) and the response variables of EVOO cv. Empeltre under deep-frying.

Response (dependent variables)	Equation (predicted value)
Fusty/muddy sediment	$Y_1=0.00$
Winey/vinegary/acid/sour	$Y_2=0.00$
Frostbitten olives	$Y_3=0.00$
Rancidity	$Y_4=5.64+0.51x_1+0.04x_2-0.31x_3-0.34x_1x_2-0.09x_1x_3-0.06x_2x_3+0.01x_1x_2x_3$
Fruity green	$Y_5=0.25-0.25x_1-0.25x_2+0.25x_3+0.25x_1x_2-0.25x_1x_3-0.25x_2x_3+0.25x_1x_2x_3$
Fruity ripe	$Y_6=0.25-0.25x_1-0.25x_2+0.25x_3+0.25x_1x_2-0.25x_1x_3-0.25x_2x_3+0.25x_1x_2x_3$
Bitter	$Y_7=1.14-0.44x_1-0.21x_2+1.14x_3-0.49x_1x_2-0.44x_1x_3-0.21x_2x_3-0.49x_1x_2x_3$
Pungent	$Y_8=1.11-0.34x_1-0.31x_2+1.11x_3-0.46x_1x_2-0.34x_1x_3-0.31x_2x_3-0.46x_1x_2x_3$

where x_1= Time; x_2= temperature; x_3= Polyphenols supplementation.

Table S6. Mathematical equation that predicts the relationship between the independent variables (time, temperature, and polyphenols addition) and the response variables of EVOO cv. Arbequina under deep-frying.

Response (dependent variables)	Equation (predicted value)
Fusty/muddy sediment	$Y_1=0.00$
Winey/vinegary/acid/sour	$Y_2=0.00$
Frostbitten olives	$Y_3=0.00$
Rancidity	$Y_4=5.62+1.02x_1+0.45x_2-1.90x_3-0.00x_1x_2+0.80x_1x_3+1.28x_2x_3-0.18x_1x_2x_3$
Fruity green	$Y_5=0.94-0.44x_1-0.24x_2+0.94x_3+0.04x_1x_2-0.44x_1x_3-0.24x_2x_3+0.04x_1x_2x_3$
Fruity ripe	$Y_6=0.94-0.44x_1-0.05x_2+1.17x_3-0.12x_1x_2+0.00x_1x_3-0.05x_2x_3-0.12x_1x_2x_3$
Bitter	$Y_7=1.17+0.00x_1-0.21x_2+1.14x_3-0.49x_1x_2-0.44x_1x_3-0.21x_2x_3-0.49x_1x_2x_3$
Pungent	$Y_8=3.80+0.05x_1-0.08x_2-0.82x_3-0.27x_1x_2-0.17x_1x_3+0.30x_2x_3+0.10x_1x_2x_3$

where x_1= Time; x_2= temperature; x_3= Polyphenols supplementation.

Table S7. Mathematical equation that predicts the relationship between the independent variables (time, temperature, and polyphenols addition) and the response variables of EVOO cv. Hojiblanca under deep-frying.

Response (dependent variables)	Equation (predicted value)
Fusty/muddy sediment	$Y_1=0.00$
Winey/vinegary/acid/sour	$Y_2=0.00$
Frostbitten olives	$Y_3=0.00$
Rancidity	$Y_1=5.66+1.56x_1+0.86x_2-0.66x_3+0.36x_1x_2-0.06x_1x_3+0.79x_2x_3-0.09x_1x_2x_3$
Fruity green	$Y_2=0.24-0.24x_1+0.24x_2+0.24x_3+0.24x_1x_2-0.24x_1x_3-0.24x_2x_3+0.24x_1x_2x_3$
Fruity ripe	$Y_3= 0.24-0.24x_1+0.24x_2+0.24x_3+0.24x_1x_2-0.24x_1x_3-0.24x_2x_3+0.24x_1x_2x_3$
Bitter	$Y_4=0.60+0.03x_1-0.60x_2+0.60x_3-0.03x_1x_2+0.03x_1x_3-0.60x_2x_3-0.03x_1x_2x_3$
Pungent	$Y_5=0.89-0.59x_1-0.51x_2-0.04x_3+0.21x_1x_2+0.34x_1x_3-0.34x_2x_3+0.04x_1x_2x_3$

where x_1= Time; x_2= temperature; x_3= Polyphenols supplementation.

Table S8. Mathematical equation that predicts the relationship between the independent variables (time, temperature, and polyphenols addition) and the response variables of EVOO cv. Manzanilla under deep-frying.

Response (dependent variables)	Equation (predicted value)
Fusty/muddy sediment	$Y_1=0.00$
Winey/vinegary/acid/sour	$Y_2=0.00$
Frostbitten olives	$Y_3=0.00$
Rancidity	$Y_4=6.60+0.78x_1+0.70x_2-0.58x_3+0.23x_1x_2+0.30x_1x_3+0.83x_2x_3-0.05x_1x_2x_3$
Fruity green	$Y_5=0.46-0.46x_1+0.04x_2-0.04x_3-0.04x_1x_2+0.04x_1x_3-0.46x_2x_3+0.46x_1x_2x_3$
Fruity ripe	$Y_6=0.46-0.46x_1+0.04x_2-0.04x_3-0.04x_1x_2+0.04x_1x_3-0.46x_2x_3+0.46x_1x_2x_3$
Bitter	$Y_7=0.28-0.28x_1+0.03x_2-0.03x_3-0.03x_1x_2+0.03x_1x_3-0.28x_2x_3+0.28x_1x_2x_3$
Pungent	$Y_8=1.90-0.85x_1-0.83x_2-1.23x_3-0.23x_1x_2+0.18x_1x_3+0.15x_2x_3+0.90x_1x_2x_3$

where x_1= Time; x_2= temperature; x_3= Polyphenols supplementation.

Table S9. Mathematical equation that predicts the relationship between the independent variables (time, temperature, and polyphenols addition) and the response variables of EVOO cv. Royuela under deep-frying.

Response (dependent variables)	Equation (predicted value)
Fusty/muddy sediment	Y_1=0.00
Winey/vinegary/acid/sour	Y_2=0.00
Frostbitten olives	Y_3=0.00
Rancidity	Y_4=5.35+1.25x_1+1.33x_2-0.45x_3-0.33x_1x_2-0.20x_1x_3+0.48x_2x_3-0.02$x_1x_2x_3$
Fruity green	Y_5=1.25-0.53x_1-1.25x_2+0.60x_3+0.53x_1x_2+0.13x_1x_3-0.60x_2x_3-0.13$x_1x_2x_3$
Fruity ripe	Y_6=0.00
Bitter	Y_7=1.99-0.24x_1-1.36x_2+0.66x_3-0.39x_1x_2-0.16x_1x_3-0.04x_2x_3-0.46$x_1x_2x_3$
Pungent	Y_8=3.30-0.05x_1-0.75x_2-0.15x_3+0.45x_1x_2+0.40x_1x_3-0.40x_2x_3+0.00$x_1x_2x_3$

where x_1= Time; x_2= temperature; x_3= Polyphenols supplementation.

Table S10. Mathematical equation that predicts the relationship between the independent variables (time, temperature, and polyphenols addition) and the response variables of Orujo olive oil under deep-frying.

Response (dependent variables)	Equation (predicted value)
Fusty/muddy sediment	Y_1=0.00
Winey/vinegary/acid/sour	Y_2=0.00
Frostbitten olives	Y_3=0.00
Rancidity	Y_4=4.18+0.68x_1-0.45x_2-0.78x_3-0.15x_1x_2-0.38x_1x_3-0.35x_2x_3-0.25$x_1x_2x_3$
Fruity green	Y_5=1.49-0.61x_1-0.01x_2+0.39x_3+0.14x_1x_2+0.49x_1x_3-0.01x_2x_3+0.14$x_1x_2x_3$
Fruity ripe	Y_6=1.49-0.61x_1-0.01x_2+0.39x_3+0.14x_1x_2+0.49x_1x_3-0.01x_2x_3+0.14$x_1x_2x_3$
Bitter	Y_7=0.21-0.21x_1-0.21x_2+0.21x_3+0.21x_1x_2-0.21x_1x_3-0.21x_2x_3+0.21$x_1x_2x_3$
Pungent	Y_8=0.00

where x_1= Time; x_2= temperature; x_3= Polyphenols supplementation.

Table S11. Mathematical equation that predicts the relationship between the independent variables (time, temperature, and polyphenols addition) and the response variables of EVOO cv. Koroneiki under deep-frying.

Response (dependent variables)	Equation (predicted value)
Fusty/muddy sediment	Y_1=0.00
Winey/vinegary/acid/sour	Y_2=0.00
Frostbitten olives	Y_3=0.00
Rancidity	Y_4=5.93+1.23x_1+0.98x_2-0.15x_3+0.28x_1x_2-0.30x_1x_3+0.05x_2x_3-0.25$x_1x_2x_3$
Fruity green	Y_5=0.90-0.90x_1-0.05x_2+0.15x_3+0.05x_1x_2-0.15x_1x_3-0.20x_2x_3+0.20$x_1x_2x_3$
Fruity ripe	Y_6=0.90-0.90x_1-0.05x_2+0.15x_3+0.05x_1x_2-0.15x_1x_3-0.20x_2x_3+0.20$x_1x_2x_3$
Bitter	Y_7=2.58-1.00x_1-0.60x_2+0.70x_3+0.03x_1x_2+0.88x_1x_3-0.63x_2x_3+0.05$x_1x_2x_3$
Pungent	Y_8=2.48-1.03x_1-0.02x_2+0.48x_3-0.08x_1x_2+0.98x_1x_3-0.23x_2x_3+0.13$x_1x_2x_3$

where x_1= Time; x_2= temperature; x_3= Polyphenols supplementation.

Table S12. Mathematical equation that predicts the relationship between the independent variables (time, temperature, and polyphenols addition) and the response variables of EVOO cv. Arbosana under deep-frying.

**Response (dependent variables)**	**Equation (predicted value)**
Fusty/muddy sediment	$Y_1=0.00$
Winey/vinegary/acid/sour	$Y_2=0.00$
Frostbitten olives	$Y_3=0.00$
Rancidity	$Y_1=6.34+0.79x_1+0.91x_2-0.84x_3-0.04x_1x_2-0.04x_1x_3+0.69x_2x_3-0.01x_1x_2x_3$
Fruity green	$Y_2=0.86-0.31x_1-0.86x_2+0.86x_3+0.31x_1x_2-0.31x_1x_3-0.86x_2x_3+0.31x_1x_2x_3$
Fruity ripe	$Y_3=0.86-0.31x_1-0.86x_2+0.86x_3+0.31x_1x_2-0.31x_1x_3-0.86x_2x_3+0.31x_1x_2x_3$
Bitter	$Y_4=2.31-0.26x_1-0.49x_2+1.49x_3+0.39x_1x_2+0.56x_1x_3+0.34x_2x_3-0.44x_1x_2x_3$
Pungent	$Y_5=2.18-0.60x_1-0.55x_2+1.20x_3+0.43x_1x_2+0.38x_1x_3+0.43x_2x_3-0.55x_1x_2x_3$

where x_1= Time; x_2= temperature; x_3= Polyphenols supplementation.

Table S13. Mathematical equation that predicts the relationship between the independent variables (time, temperature, and polyphenols addition) and the response variables of olive oil 1° under deep-frying.

**Response (dependent variables)**	**Equation (predicted value)**
Fusty/muddy sediment	$Y_1=0.50-0.05x_1-0.50x_2+0.50x_3+0.05x_1x_2-0.05x_1x_3-0.50x_2x_3+0.05x_1x_2x_3$
Winey/vinegary/acid/sour	$Y_2=0.84-0.29x_1-0.84x_2+0.36x_3+0.29x_1x_2+0.19x_1x_3-0.36x_2x_3-0.19x_1x_2x_3$
Frostbitten olives	$Y_3=0.84-0.29x_1-0.84x_2+0.36x_3+0.29x_1x_2+0.19x_1x_3-0.36x_2x_3-0.19x_1x_2x_3$
Rancidity	$Y_4=6.16+1.71x_1-0.44x_2-0.94x_3+0.46x_1x_2-0.24x_1x_3+0.41x_2x_3+0.06x_1x_2x_3$
Fruity green	$Y_5=0.44-0.24x_1-0.14x_2+0.14x_3-0.06x_1x_2+0.06x_1x_3-0.44x_2x_3+0.24x_1x_2x_3$
Fruity ripe	$Y_6=0.44-0.24x_1-0.14x_2+0.14x_3-0.06x_1x_2+0.06x_1x_3-0.44x_2x_3+0.24x_1x_2x_3$
Bitter	$Y_7=1.23-0.50x_1-0.58x_2+0.68x_3-0.15x_1x_2+0.05x_1x_3-0.03x_2x_3-0.70x_1x_2x_3$
Pungent	$Y_8=2.00-0.40x_1-0.38x_2-0.10x_3-0.38x_1x_2-0.40x_1x_3-0.23x_2x_3-0.13x_1x_2x_3$

where x_1= Time; x_2= temperature; x_3= Polyphenols supplementation.

Table S14. Mathematical equation that predicts the relationship between the independent variables (time, temperature, and polyphenols addition) and the response variables of olive oil 0.4° under deep-frying.

**Response (dependent variables)**	**Equation (predicted value)**
Fusty/muddy sediment	$Y_1=0.00$
Winey/vinegary/acid/sour	$Y_2=0.00$
Frostbitten olives	$Y_3=0.00$
Rancidity	$Y_4=5.90+1.55x_1+0.10x_2-0.50x_3+0.25x_1x_2+0.30x_1x_3-0.25x_2x_3+0.05x_1x_2x_3$
Fruity green	$Y_5=0.00$
Fruity ripe	$Y_6=0.00$
Bitter	$Y_7=0.56-0.56x_1-0.01x_2+0.56x_3+0.01x_1x_2-0.56x_1x_3-0.01x_2x_3+0.01x_1x_2x_3$
Pungent	$Y_8=0.00$

where x_1= Time; x_2= temperature; x_3= Polyphenols supplementation.

Table S15. Mathematical equation that predicts the relationship between the independent variables (time and temperature) and the response variables of sunflower oil under deep-frying.

Response (dependent variables)	Equation (predicted value)
Fusty/muddy sediment	Y_1=0.00
Winey/vinegary/acid/sour	Y_2=0.00
Frostbitten olives	Y_3=0.00
Rancidity	Y_4=5.95+1.35x_1+0.60x_2+0.80x_1x_2
Fruity green	Y_5=0.00
Fruity ripe	Y_6=0.00
Bitter	Y_7=0.00
Pungent	Y_8=0.00

where x_1= Time; x_2= temperature.

Table S16. Mathematical equation that predicts the relationship between the independent variables (time and temperature) and the response variables of sunflower oil-high oleic acid under deep-frying.

Response (dependent variables)	Equation (predicted value)
Fusty/muddy sediment	Y_1=0.00
Winey/vinegary/acid/sour	Y_2=0.00
Frostbitten olives	Y_3=0.00
Rancidity	Y_4=5.33+1.37x_1-0.18x_2+0.28x_1x_2
Fruity green	Y_5=0.00
Fruity ripe	Y_6=0.00
Bitter	Y_7=0.00
Pungent	Y_8=0.00

where x_1= Time; x_2= temperature.

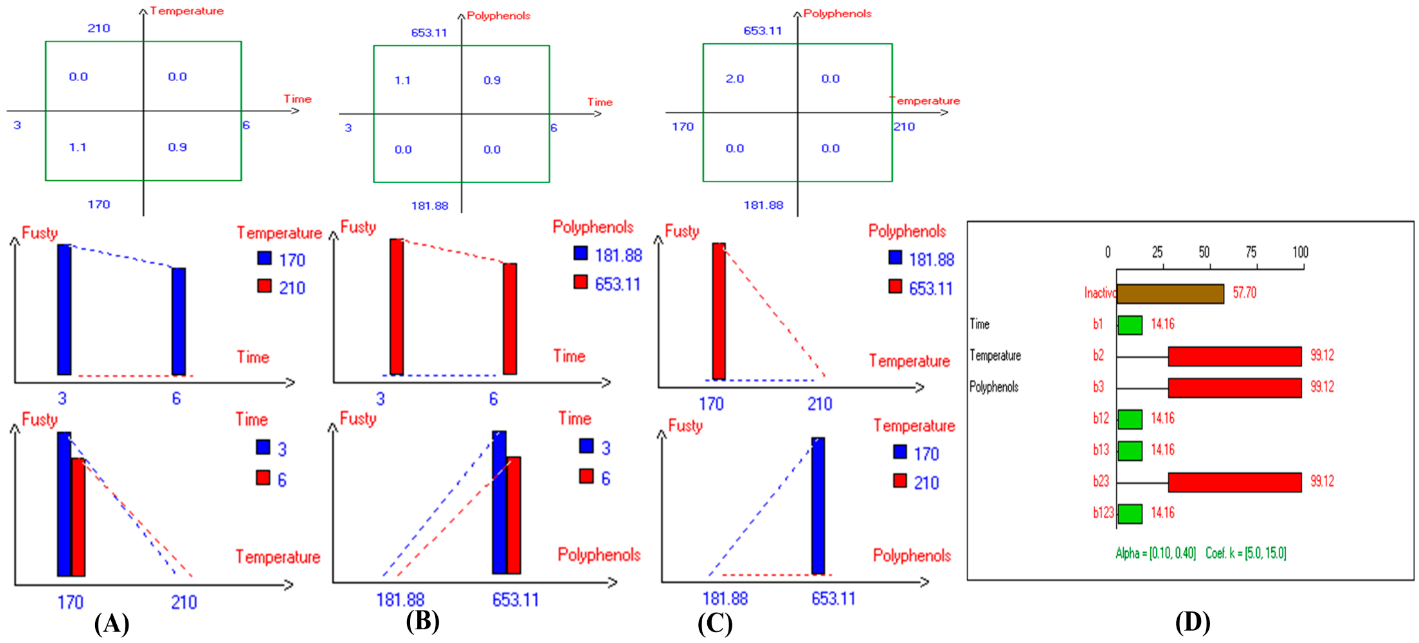

Figure S1. Combined interactions between the independent variables on fusty in olive oil 1°: (**A**) x_1 and x_2, (**B**) x_1 and x_3, (**C**) x_2 and x_3, and (**D**) Results of variance analysis of regression equation model and the significance changes of each indivdual independent variable and interaction between the combined independent variables on fusty score.

where, b represents significant difference when be >b_{123}; while b represents no significant difference when be ≤b_{123}. x_1: time, x_2: temperature, x_3: polyphenols.

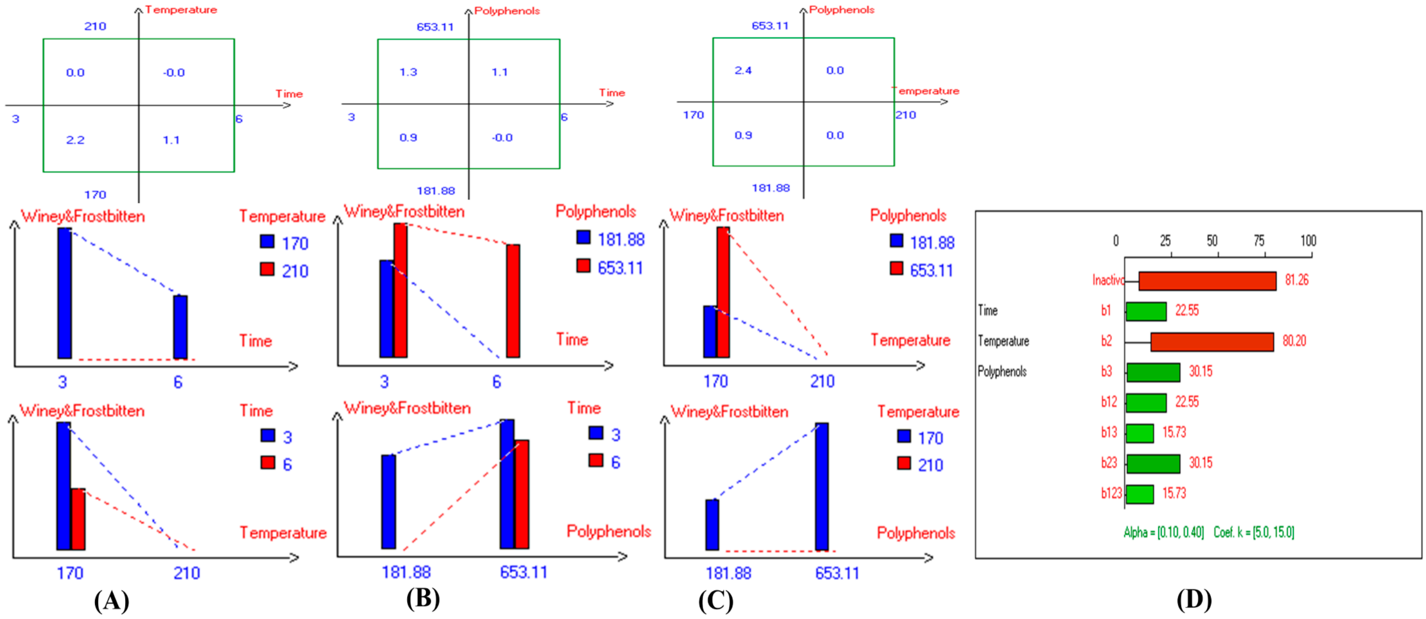

Figure S2. Combined interactions between the independent variables on winey and frostbitten olives in olive oil 1°: (**A**) x_1 and x_2, (**B**) x_1 and x_3, (**C**) x_2 and x_3, and (**D**) Results of variance analysis of regression equation model and the significance changes of each indivdual independent variable and interaction between the combined independent variables on winey and frostbitten olives scores.

where, b represents significant difference when be >b_{123}; while b represents no significant difference when be ≤b_{123}. x_1: time, x_2: temperature, x_3: polyphenols.

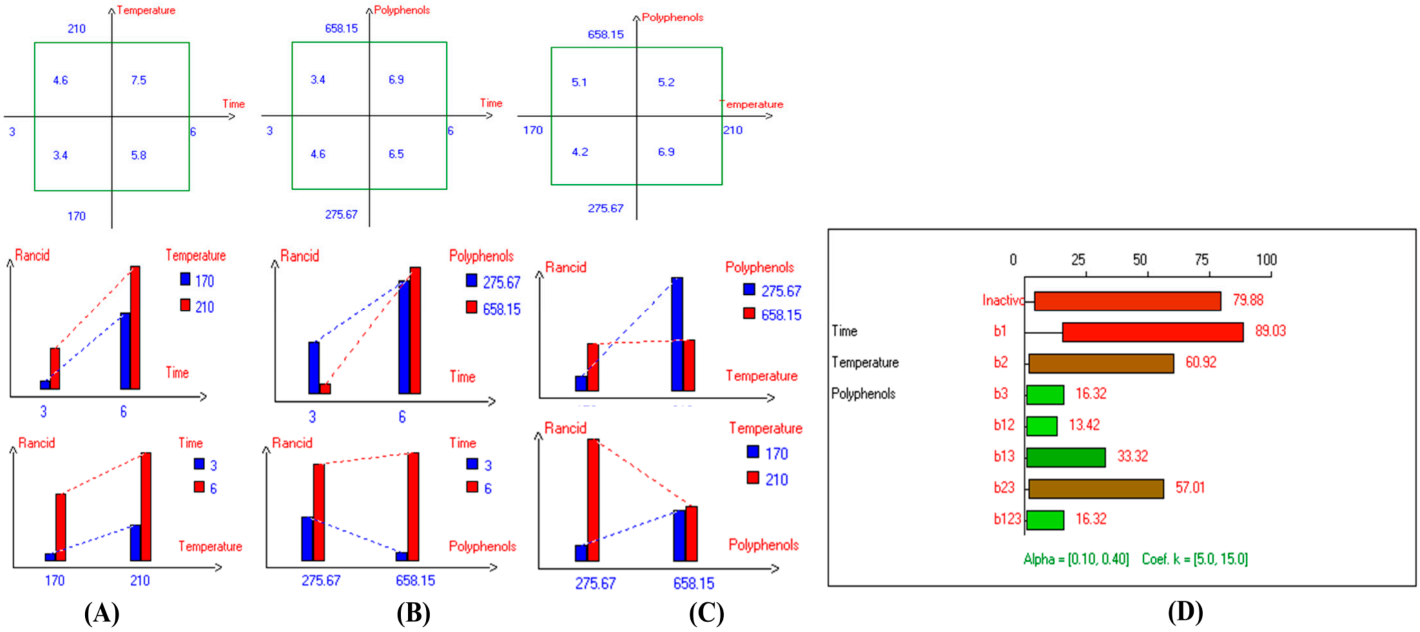

Figure S3. Combined interactions between the independent variables on rancidity in EVOO cv. Cornicabra: (**A**) x_1 and x_2, (**B**) x_1 and x_3, (**C**) x_2 and x_3, and (**D**) Results of variance analysis of regression equation model and the significance changes of each indivdual independent variable and interaction between the combined independent variables on rancid score.

where, b represents significant difference when be >b_{123}; while b represents no significant difference when be ≤b_{123}. x_1: time, x_2: temperature, x_3: polyphenols.

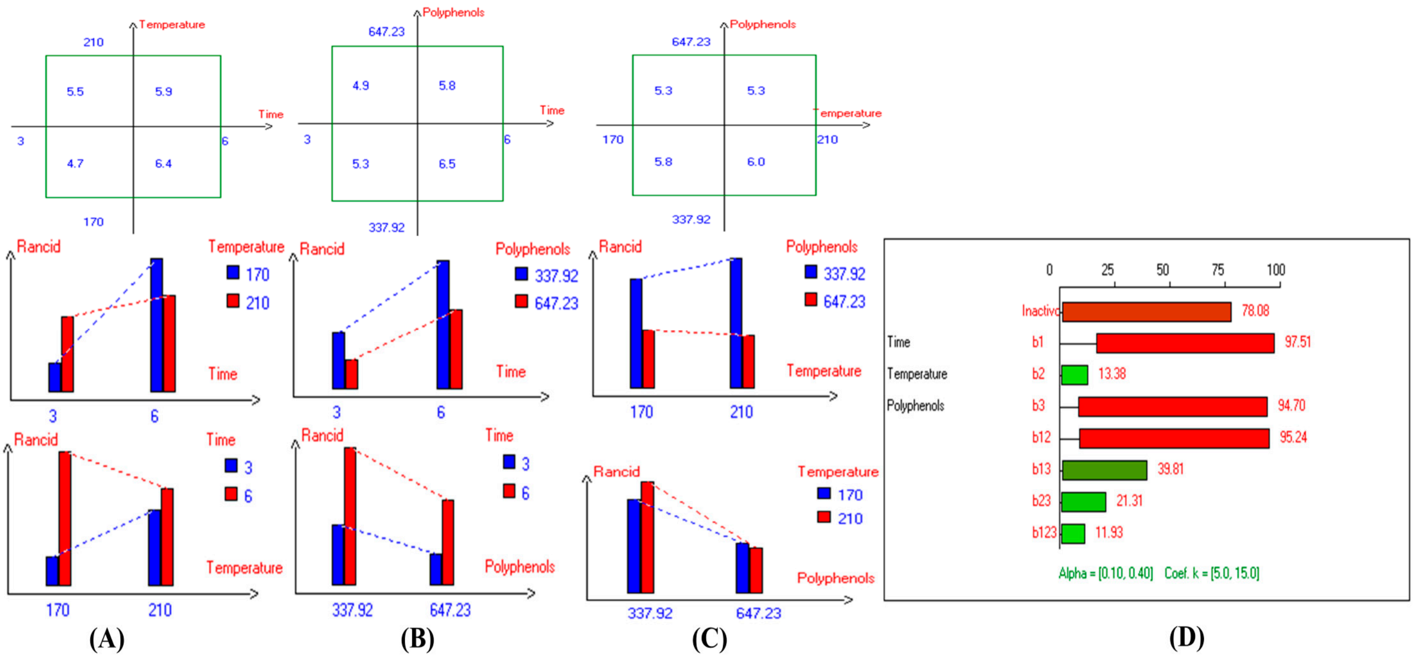

Figure S4. Combined interactions between the independent variables on rancidity in EVOO cv. Empeltre: (**A**) x_1 and x_2, (**B**) x_1 and x_3, (**C**) x_2 and x_3, and (**D**) Results of variance analysis of regression equation model and the significance changes of each indivdual independent variable and interaction between the combined independent variables on rancid score.

where, b represents significant difference when be >b_{123}; while b represents no significant difference when be ≤b_{123}. x_1: time, x_2: temperature, x_3: polyphenols.

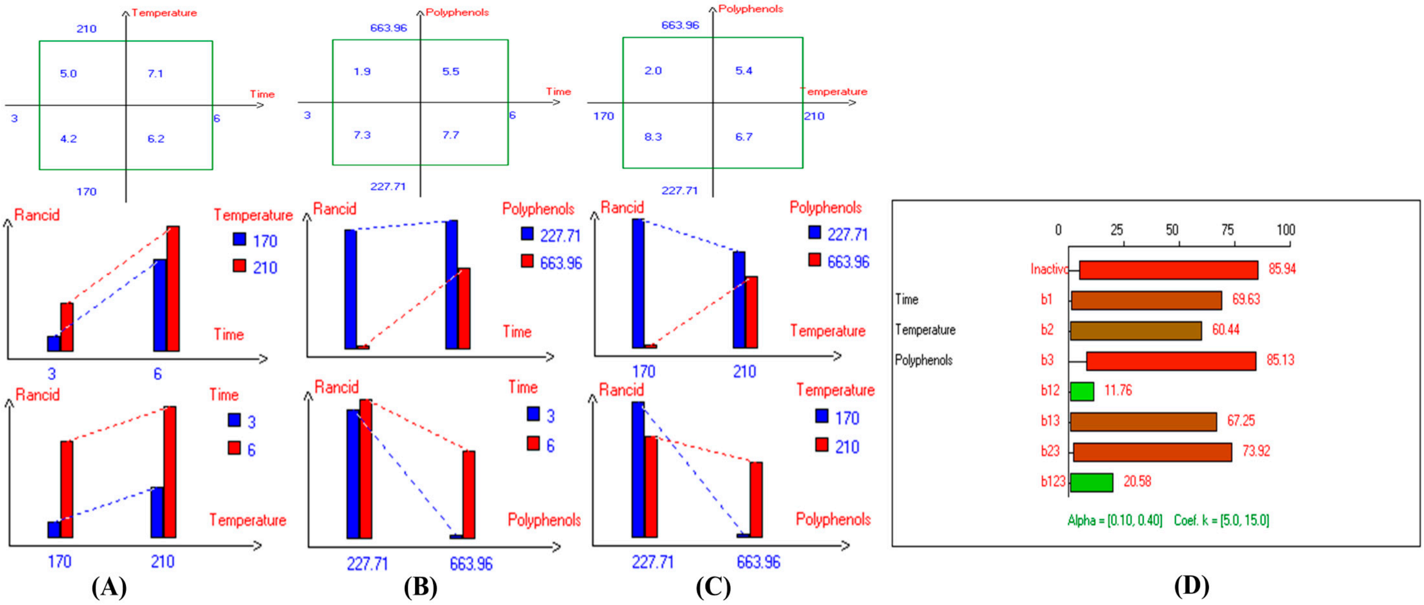

Figure S5. Combined interactions between the independent variables on rancidity in EVOO cv. Arbequina: (**A**) x_1 and x_2, (**B**) x_1 and x_3, (**C**) x_2 and x_3, and (**D**) Results of variance analysis of regression equation model and the significance changes of each indivdual independent variable and interaction between the combined independent variables on rancid score.

where, b represents significant difference when be $>b_{123}$; while b represents no significant difference when be $\leq b_{123}$. x_1: time, x_2: temperature, x_3: polyphenols.

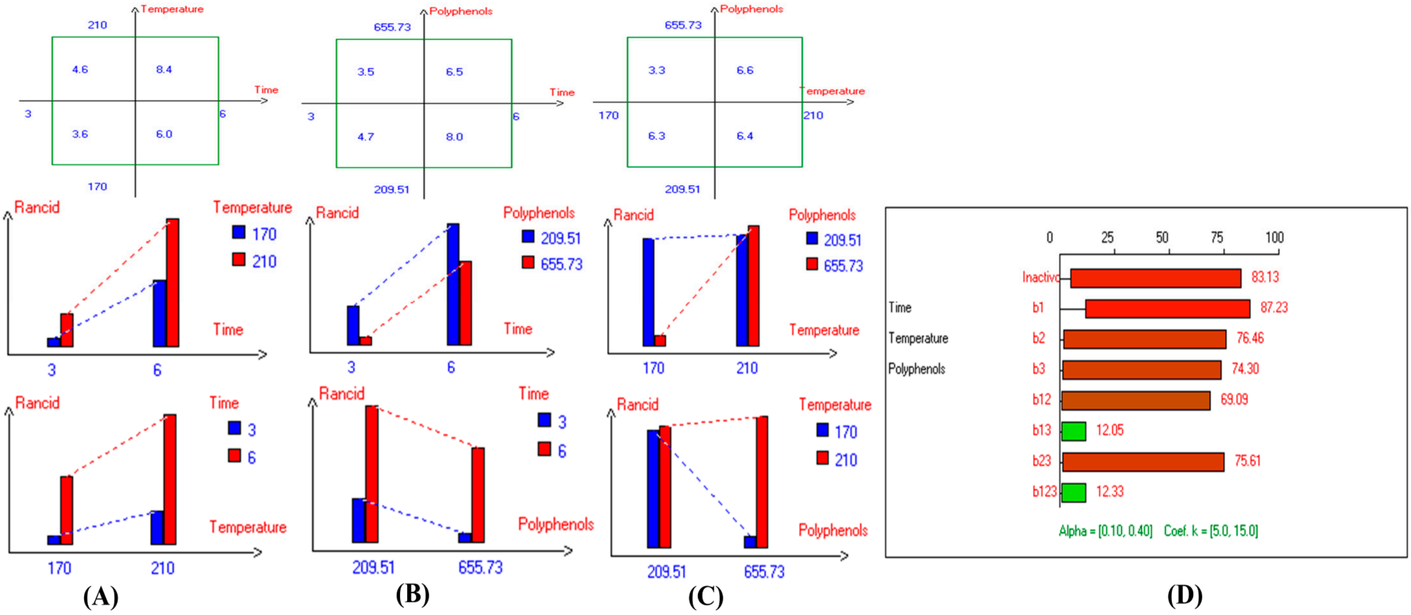

Figure S6. Combined interactions between the independent variables on rancidity in EVOO cv. Hojiblanca: (**A**) x_1 and x_2, (**B**) x_1 and x_3, (**C**) x_2 and x_3, and (**D**) Results of variance analysis of regression equation model and the significance changes of each indivdual independent variable and interaction between the combined independent variables on rancid score.

where, b represents significant difference when be $>b_{123}$; while b represents no significant difference when be $\leq b_{123}$. x_1: time, x_2: temperature, x_3: polyphenols.

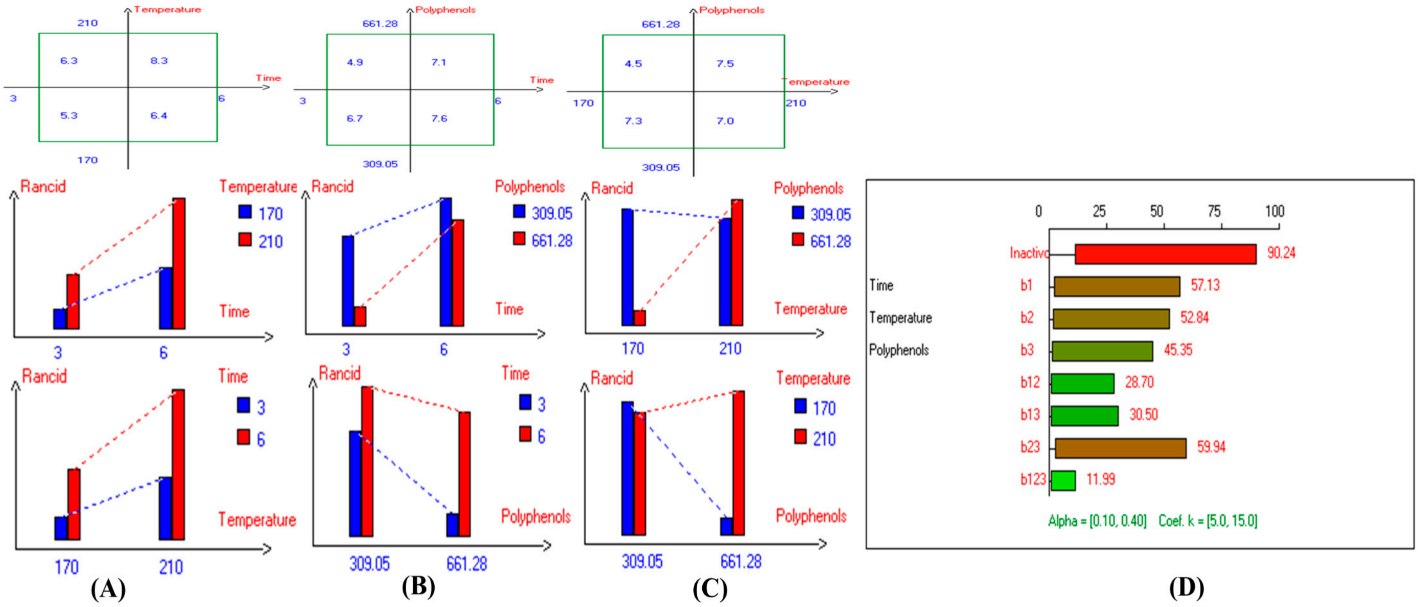

Figure S7. Combined interactions between the independent variables on rancidity in EVOO cv. Manzanilla: (**A**) x_1 and x_2, (**B**) x_1 and x_3, (**C**) x_2 and x_3, and (**D**) Results of variance analysis of regression equation model and the significance changes of each indivdual independent variable and interaction between the combined independent variables on rancid score.

where, b represents significant difference when be $>b_{123}$; while b represents no significant difference when be $\leq b_{123}$. x_1: time, x_2: temperature, x_3: polyphenols.

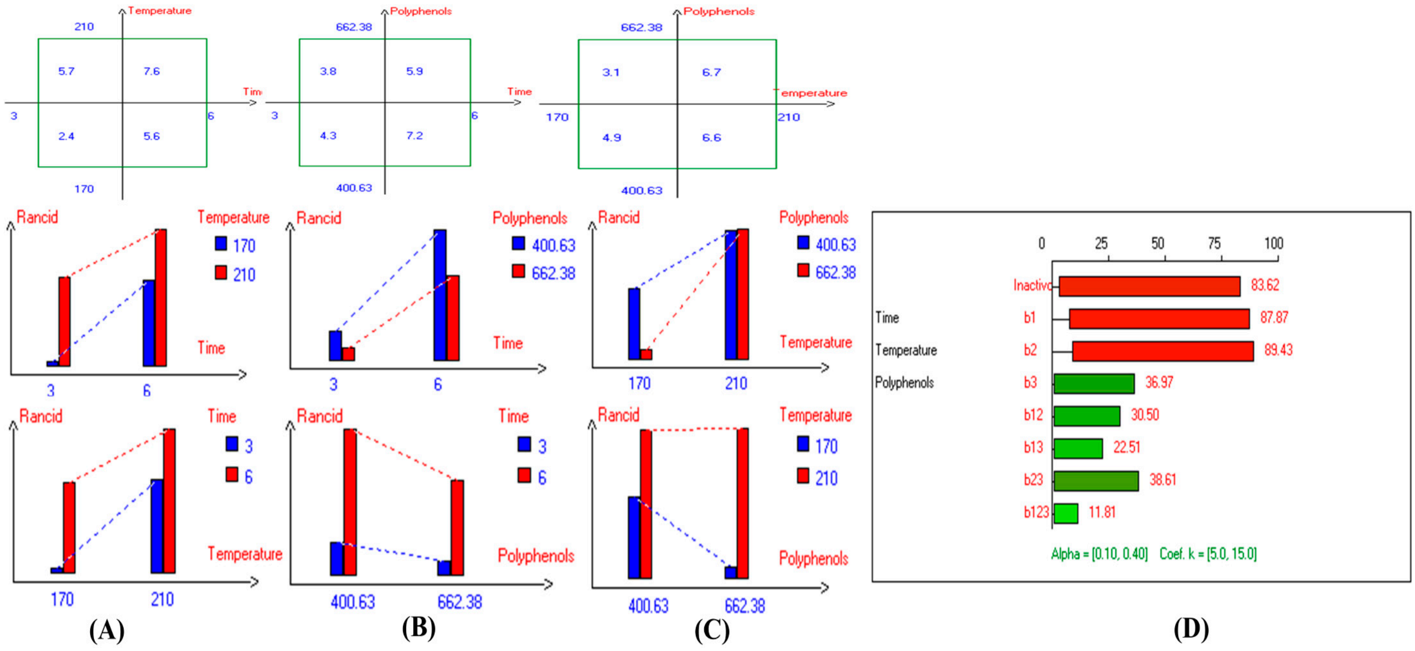

Figure S8. Combined interactions between the independent variables on rancidity in EVOO cv. Royuela: (**A**) x_1 and x_2, (**B**) x_1 and x_3, (**C**) x_2 and x_3, and (**D**) Results of variance analysis of regression equation model and the significance changes of each indivdual independent variable and interaction between the combined independent variables on rancid score.

where, b represents significant difference when be $>b_{123}$; while b represents no significant difference when be $\leq b_{123}$. x_1: time, x_2: temperature, x_3: polyphenols.

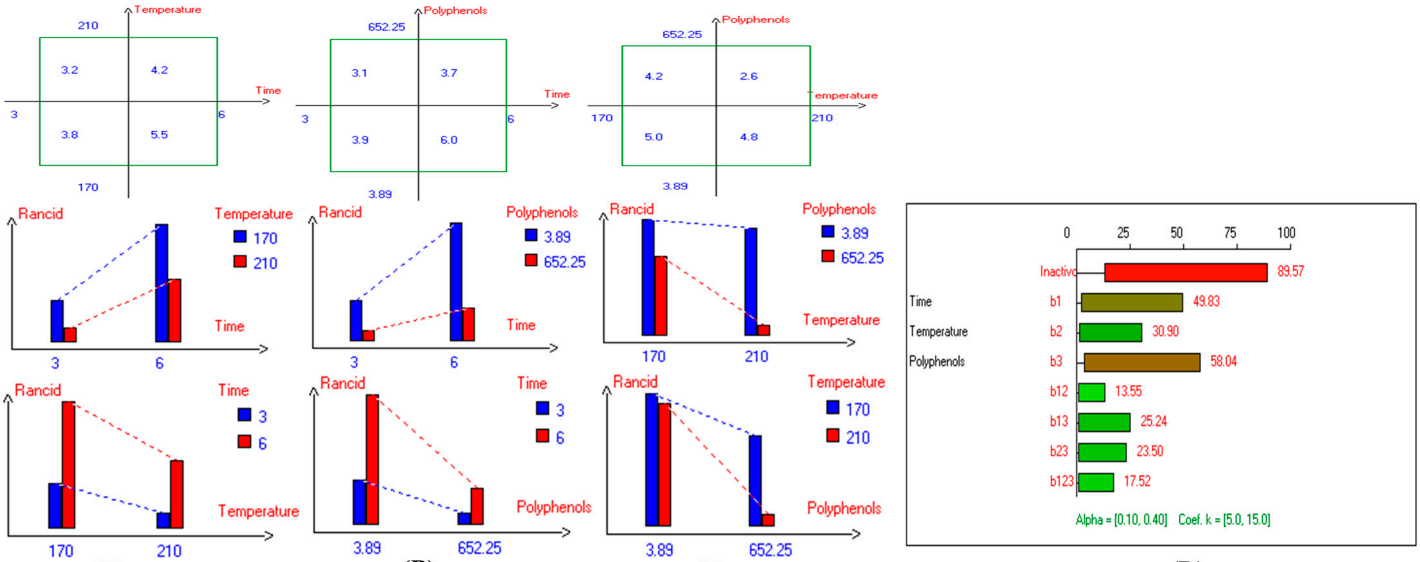

(A) (B) (C) (D)

Figure S9. Combined interactions between the independent variables on rancidity in Orujo oil: (**A**) x_1 and x_2, (**B**) x_1 and x_3, (**C**) x_2 and x_3, and (**D**) Results of variance analysis of regression equation model and the significance changes of each indivdual independent variable and interaction between the combined independent variables on rancid score.

where, b represents significant difference when be >b_{123}; while b represents no significant difference when be ≤b_{123}. x_1: time, x_2: temperature, x_3: polyphenols.

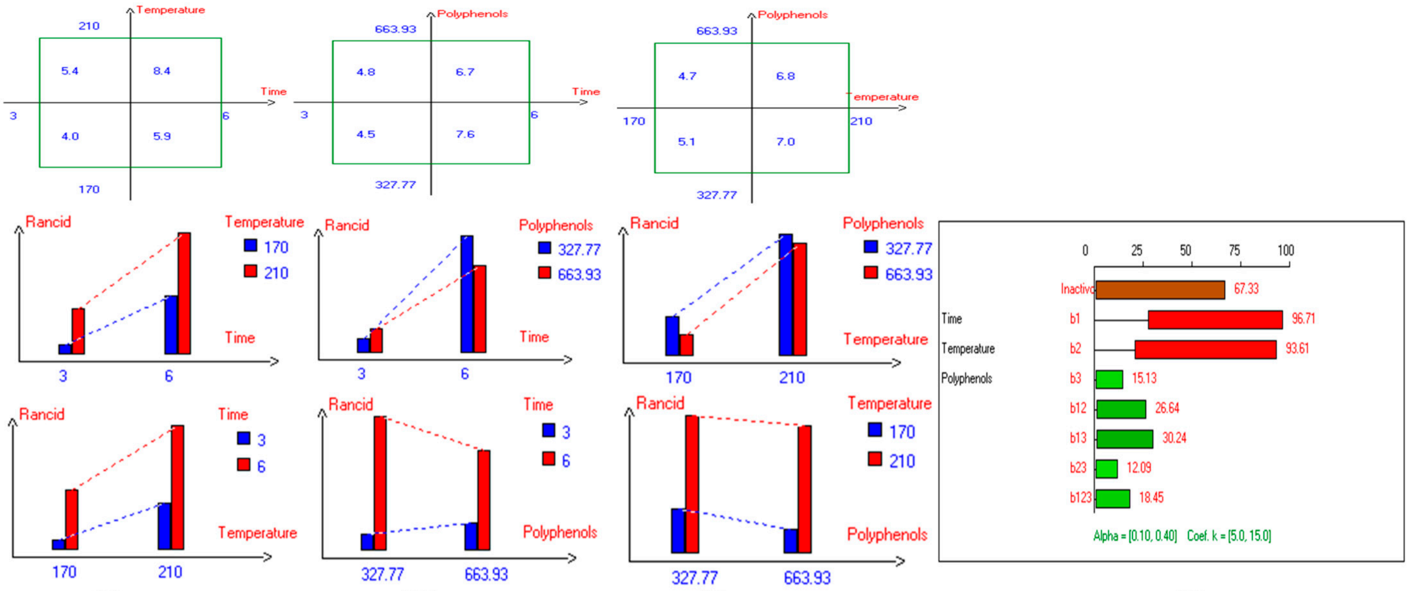

(A) (B) (C) (D)

Figure S10. Combined interactions between the independent variables on rancidity in EVOO cv. Koroneiki : (**A**) x_1 and x_2, (**B**) x_1 and x_3, (**C**) x_2 and x_3, and (**D**) Results of variance analysis of regression equation model and the significance changes of each indivdual independent variable and interaction between the combined independent variables on rancid score.

where, b represents significant difference when be >b_{123}; while b represents no significant difference when be ≤b_{123}. x_1: time, x_2: temperature, x_3: polyphenols.

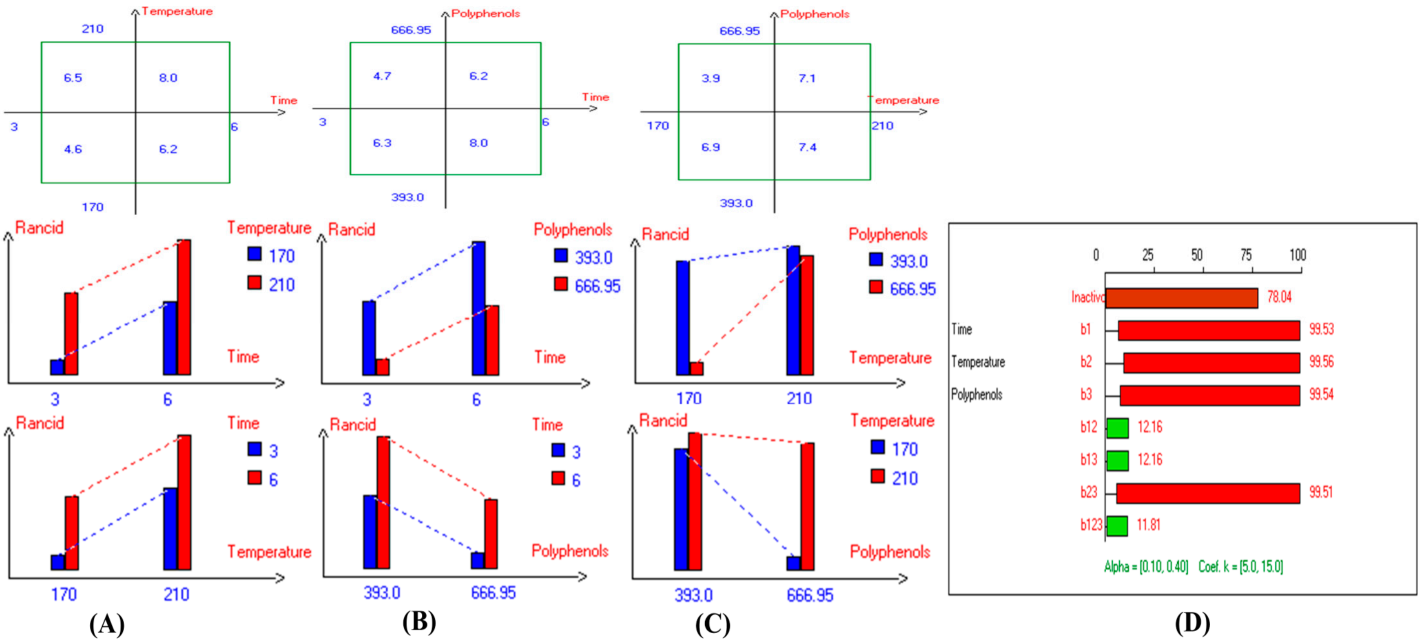

Figure S11. Combined interactions between the independent variables on rancidity in EVOO cv. Arbosana: (**A**) x_1 and x_2, (**B**) x_1 and x_3, (**C**) x_2 and x_3, and (**D**) Results of variance analysis of regression equation model and the significance changes of each indivdual independent variable and interaction between the combined independent variables on rancidity score.

where, b represents significant difference when be >b_{123}; while b represents no significant difference when be ≤b_{123}. x_1: time, x_2: temperature, x_3: polyphenols.

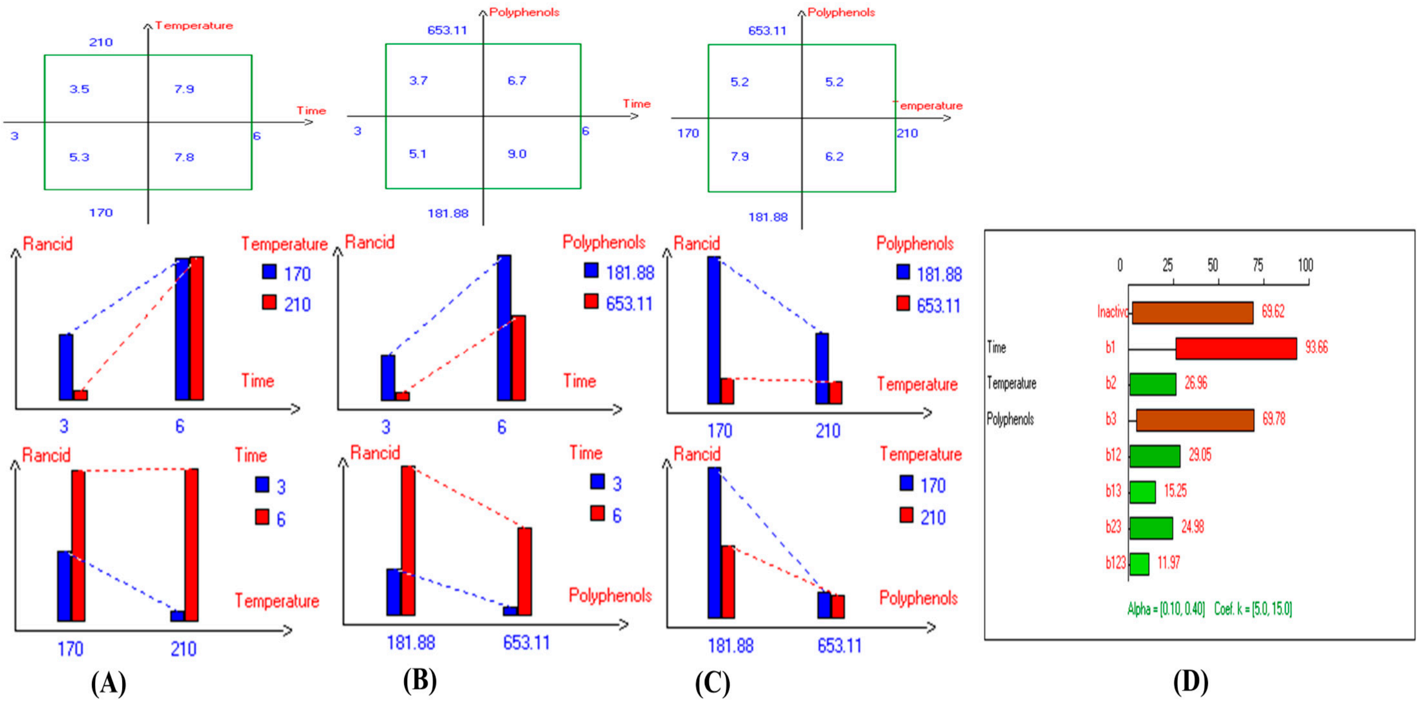

Figure S12. Combined interactions between the independent variables on rancidity in olive oil 1°: (**A**) x_1 and x_2, (**B**) x_1 and x_3, (**C**) x_2 and x_3, and (**D**) Results of variance analysis of regression equation model and the significance changes of each indivdual independent variable and interaction between the combined independent variables on rancid score.

where, b represents significant difference when be >b_{123}; while b represents no significant difference when be ≤b_{123}. x_1: time, x_2: temperature, x_3: polyphenols.

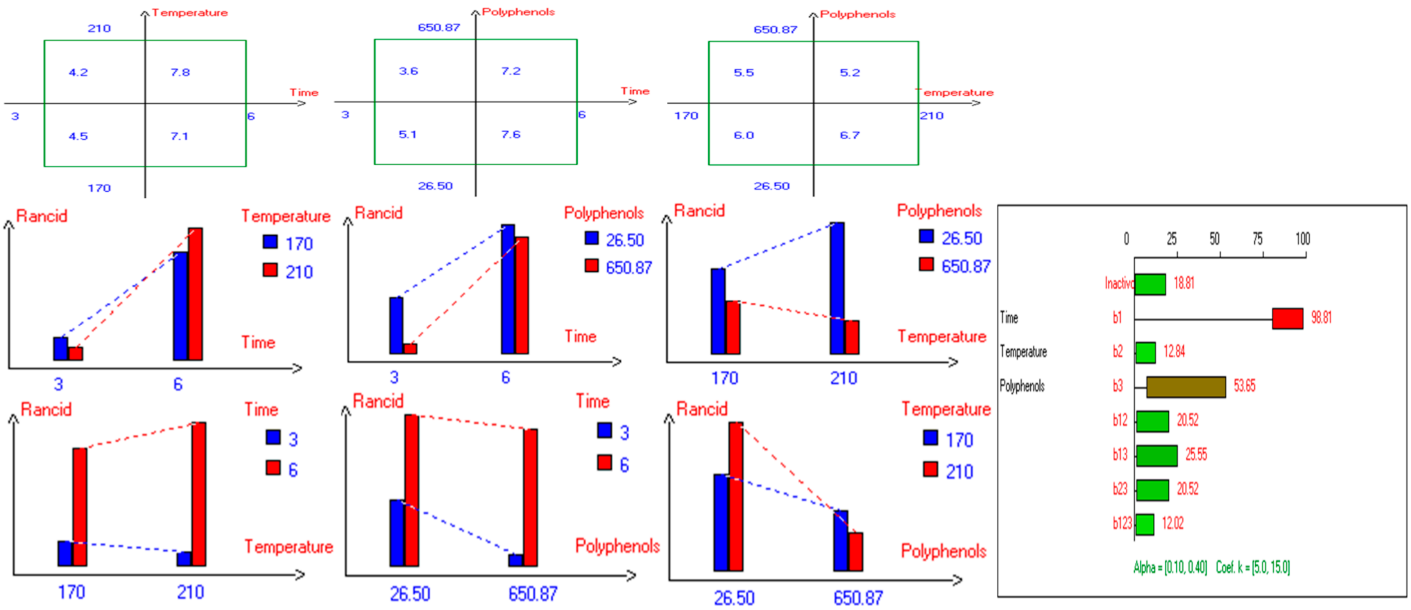

(A) (B) (C) (D)

Figure S13. Combined interactions between the independent variables on rancidity in olive oil 0.4°: (**A**) x_1 and x_2, (**B**) x_1 and x_3, (**C**) x_2 and x_3, and (**D**) Results of variance analysis of regression equation model and the significance changes of each indivdual independent variable and interaction between the combined independent variables on rancid score.

where, b represents significant difference when be >b_{123}; while b represents no significant difference when be ≤b_{123}. x_1: time, x_2: temperature, x_3: polyphenols.

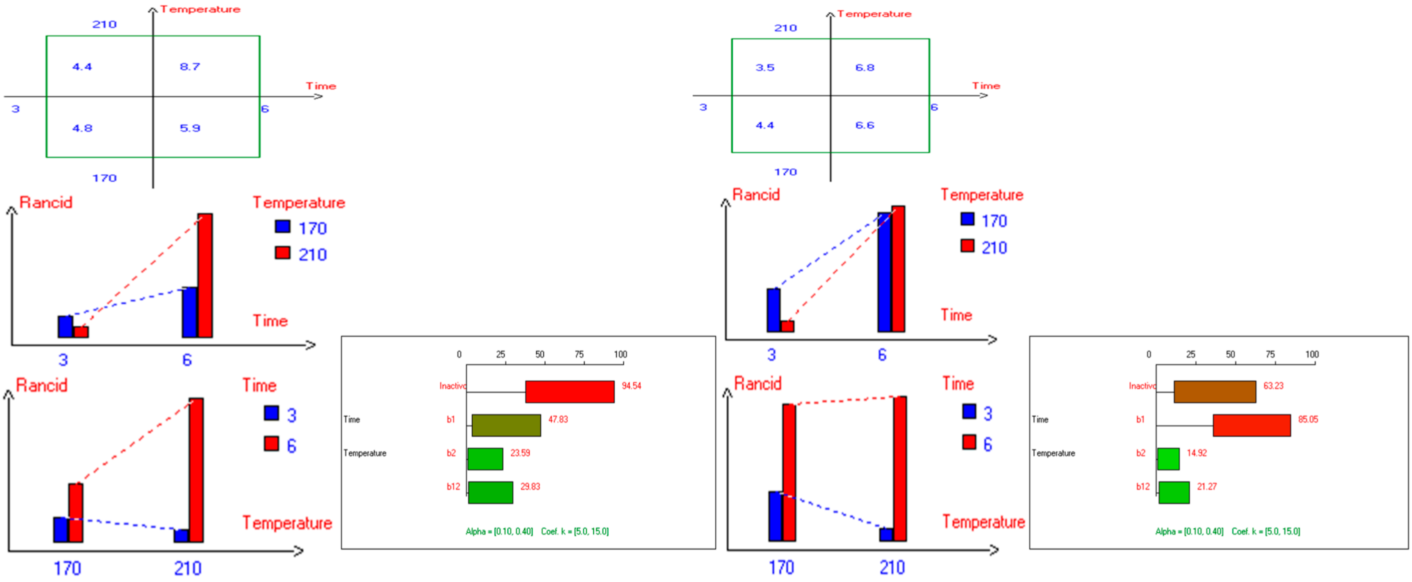

(A) (B) (C) (D)

Figure S14. (**A**) Combined interactions between the independent variables (x_1 and x_2) on rancidity in sunflower oil, (**B)** Results of variance analysis of regression equation model and the significance changes of each indivdual independent variable and interaction between the combined independent variables on rancid score in sunflower oil. (**C**) Combined interactions between the independent variables (x_1 and x_2) on rancidity in sunflower oil-high oleic acid, (**D**) Results of variance analysis of regression equation model and the significance changes of each indivdual independent variable and interaction between the combined independent variables on rancid score in sunflwoer oil-high oleic acid.

where, b represents significant difference when be >b_{12}; while b represents no significant difference when be ≤b_{12}. Where x_1: time, x_2: temperature.

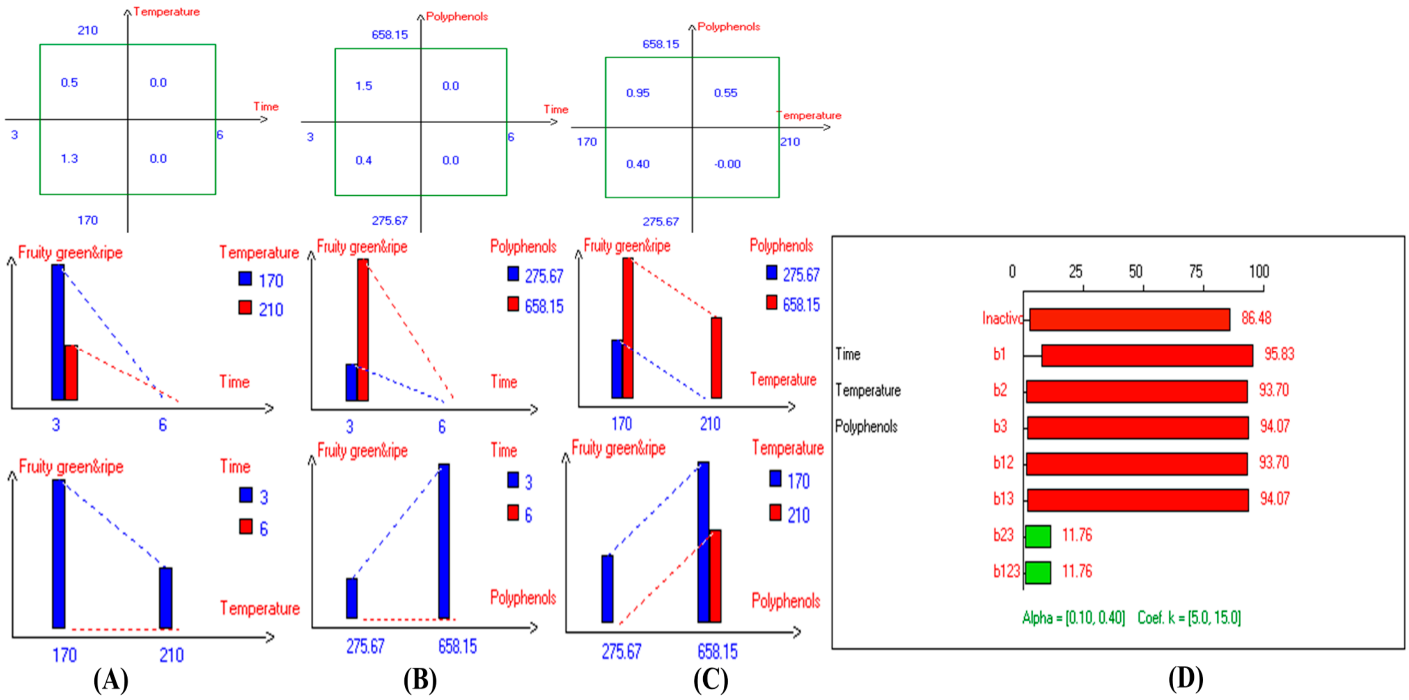

Figure S15. Combined interactions between the independent variables on fruity in EVOO cv. Cornicabra: (**A**) x_1 and x_2, (**B**) x_1 and x_3, (**C**) x_2 and x_3, and (**D**) Results of variance analysis of regression equation model and the significance changes of each indivdual independent variable and interaction between the combined independent variables on fruity green& ripe score.

where, b represents significant difference when be $>b_{123}$; while b represents no significant difference when be $\leq b_{123}$. x_1: time, x_2: temperature, x_3: polyphenols.

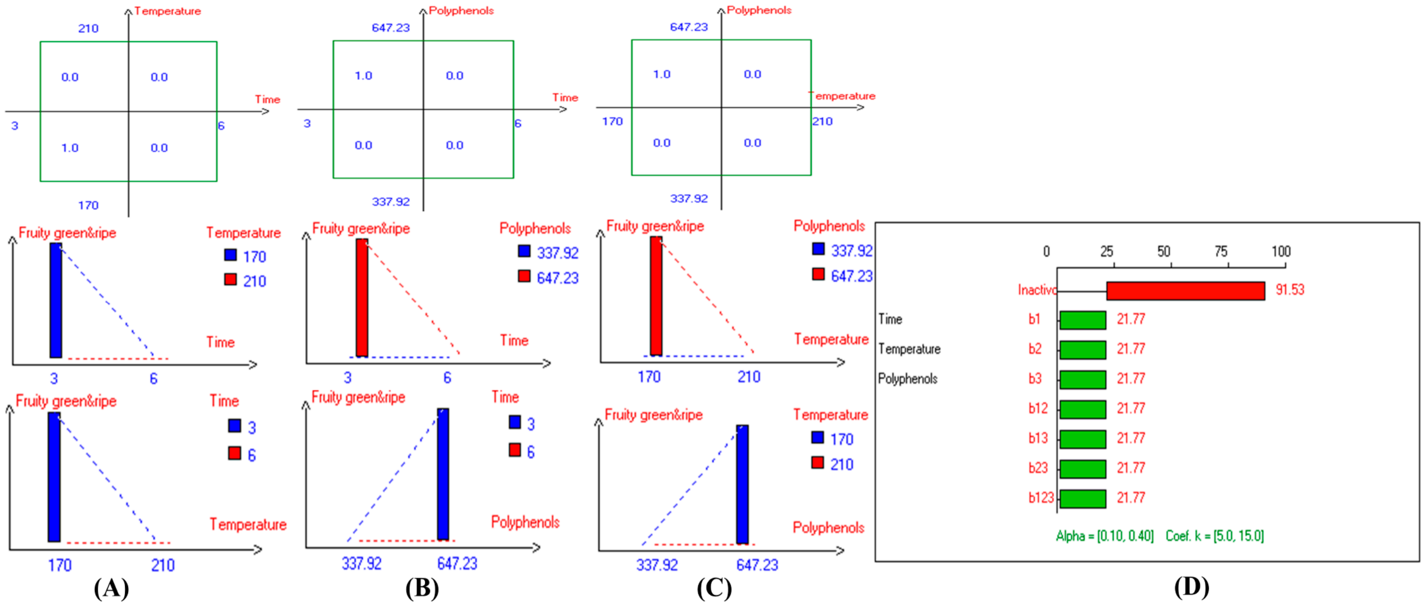

Figure S16. Combined interactions between the independent variables on fruity in EVOO cv. Empeltre: (**A**) x_1 and x_2, (**B**) x_1 and x_3, (**C**) x_2 and x_3, and (**D**) Results of variance analysis of regression equation model and the significance changes of each indivdual independent variable and interaction between the combined independent variables on fruity score.

where, b represents significant difference when be $>b_{123}$; while b represents no significant difference when be $\leq b_{123}$. x_1: time, x_2: temperature, x_3: polyphenols.

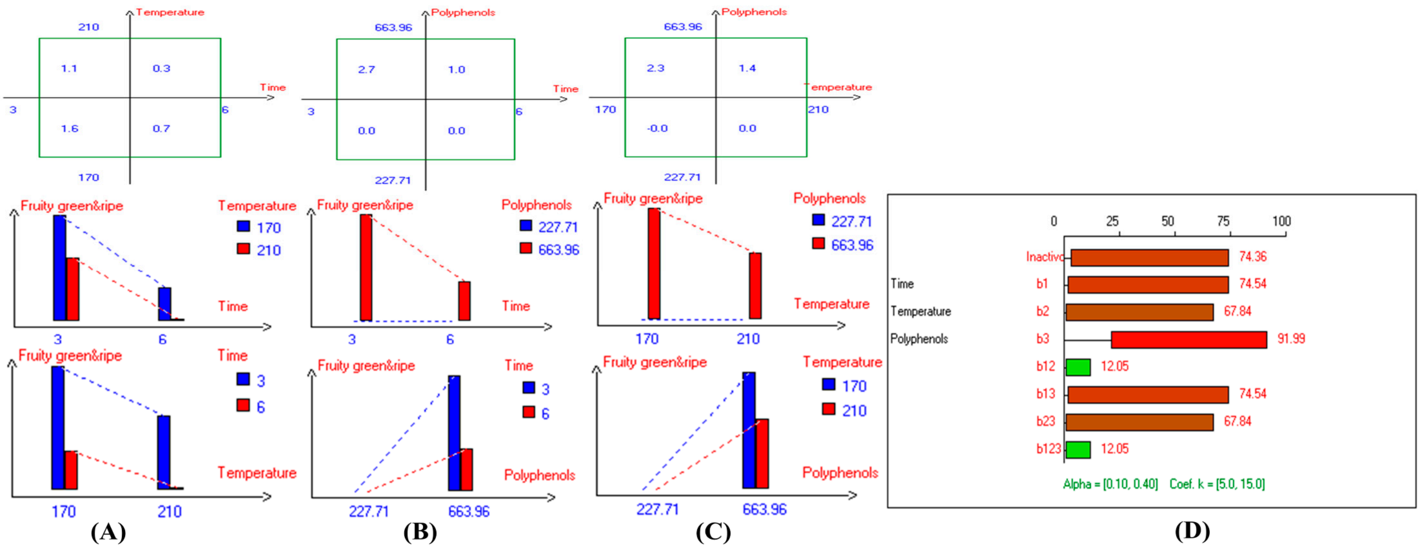

(A) **(B)** **(C)** **(D)**

Figure S17. Combined interactions between the independent variables on fruity in EVOO cv. Arbequina: (**A**) x_1 and x_2, (**B**) x_1 and x_3, (**C**) x_2 and x_3, and (**D**) Results of variance analysis of regression equation model and the significance changes of each indivdual independent variable and interaction between the combined independent variables on fruity score.

where, b represents significant difference when be $>b_{123}$; while b represents no significant difference when be $\leq b_{123}$. x_1: time, x_2: temperature, x_3: polyphenols.

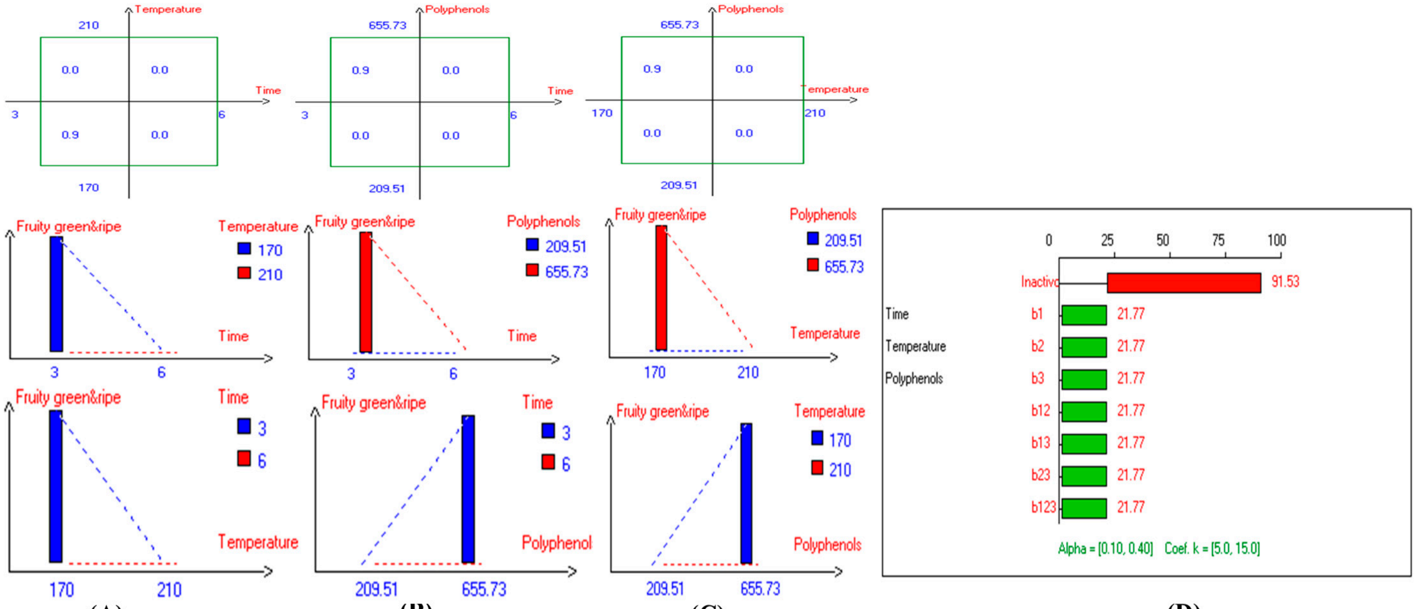

(A) **(B)** **(C)** **(D)**

Figure S18. Combined interactions between the independent variables on fruity in EVOO cv. Hojiblanca: (**A**) x_1 and x_2, (**B**) x_1 and x_3, (**C**) x_2 and x_3, and (**D**) Results of variance analysis of regression equation model and the significance changes of each indivdual independent variable and interaction between the combined independent variables on fruity score.

where, b represents significant difference when be $>b_{123}$; while b represents no significant difference when be $\leq b_{123}$. x_1: time, x_2: temperature, x_3: polyphenols.

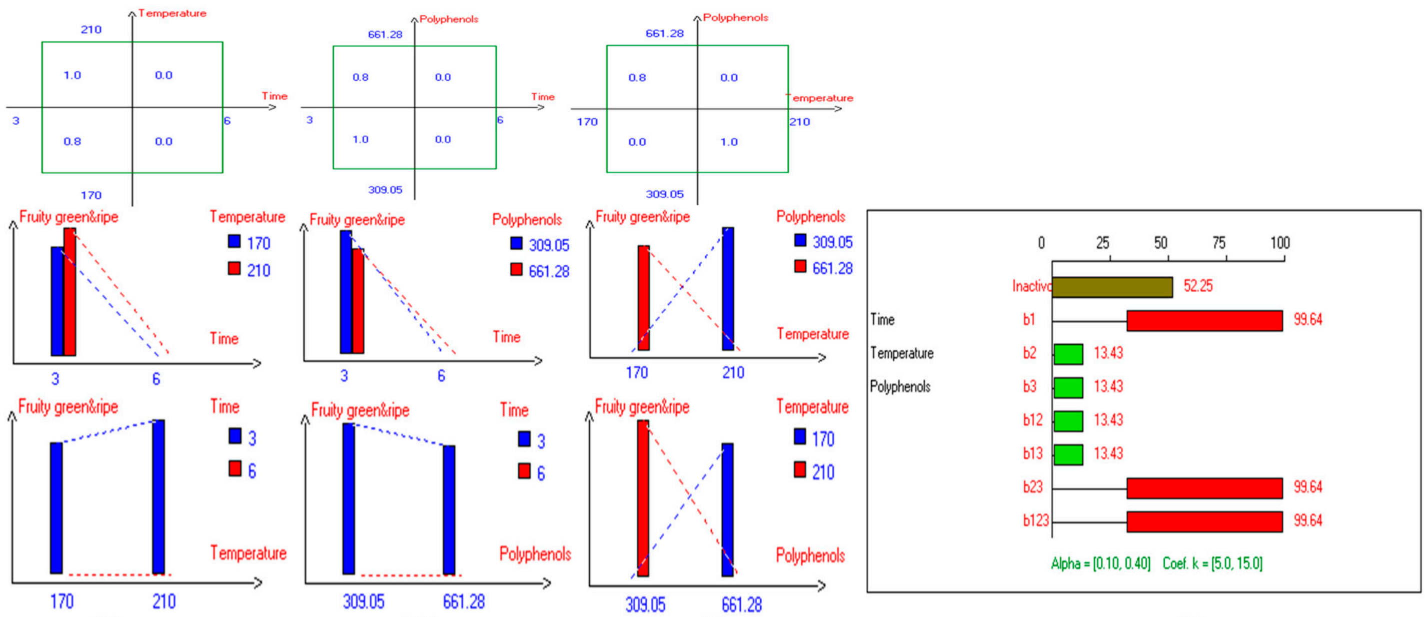

(A) (B) (C) (D)

Figure S19. Combined interactions between the independent variables on fruity in EVOO cv. Manzanilla: (**A**) x_1 and x_2, (**B**) x_1 and x_3, (**C**) x_2 and x_3, and (**D**) Results of variance analysis of regression equation model and the significance changes of each indivdual independent variable and interaction between the combined independent variables on fruity score.

where, b represents significant difference when be >b_{123}; while b represents no significant difference when be ≤b_{123}. x_1: time, x_2: temperature, x_3: polyphenols.

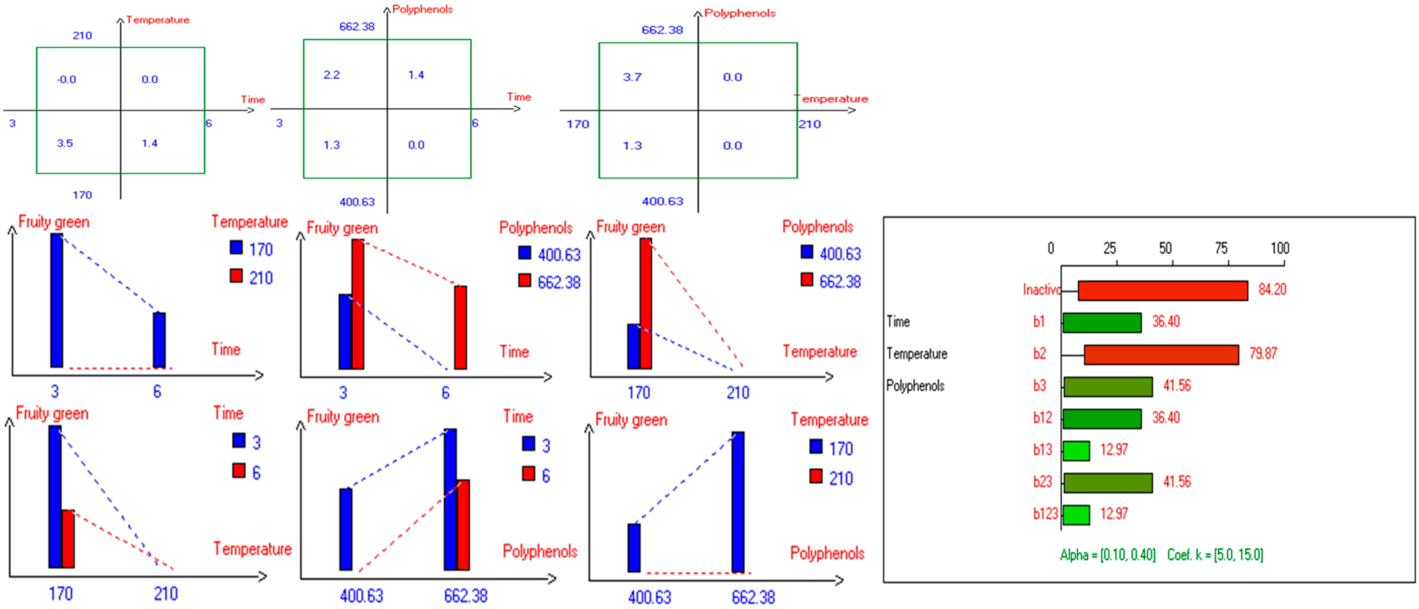

(A) (B) (C) (D)

Figure S20. Combined interactions between the independent variables on fruity green in EVOO cv. Royuela: (**A**) x_1 and x_2, (**B**) x_1 and x_3, (**C**) x_2 and x_3, and (**D**) Results of variance analysis of regression equation model and the significance changes of each indivdual independent variable and interaction between the combined independent variables on fruity green score.

where, b represents significant difference when be >b_{123}; while b represents no significant difference when be ≤b_{123}. x_1: time, x_2: temperature, x_3: polyphenols.

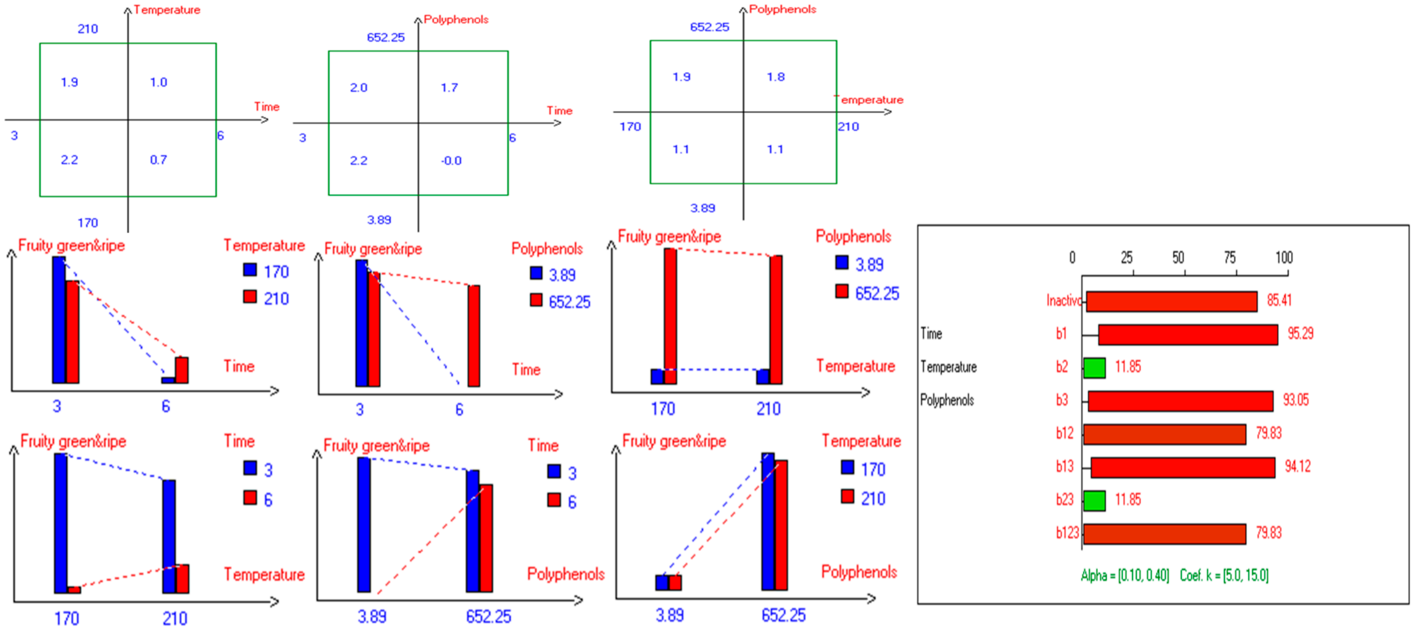

(A) (B) (C) (D)

Figure S21. Combined interactions between the independent variables on fruity in Orujo oil: (**A**) x_1 and x_2, (**B**) x_1 and x_3, (**C**) x_2 and x_3, and (**D**) Results of variance analysis of regression equation model and the significance changes of each indivdual independent variable and interaction between the combined independent variables on fruity score.

where, b represents significant difference when be >b_{123}; while b represents no significant difference when be ≤b_{123}. x_1: time, x_2: temperature, x_3: polyphenols.

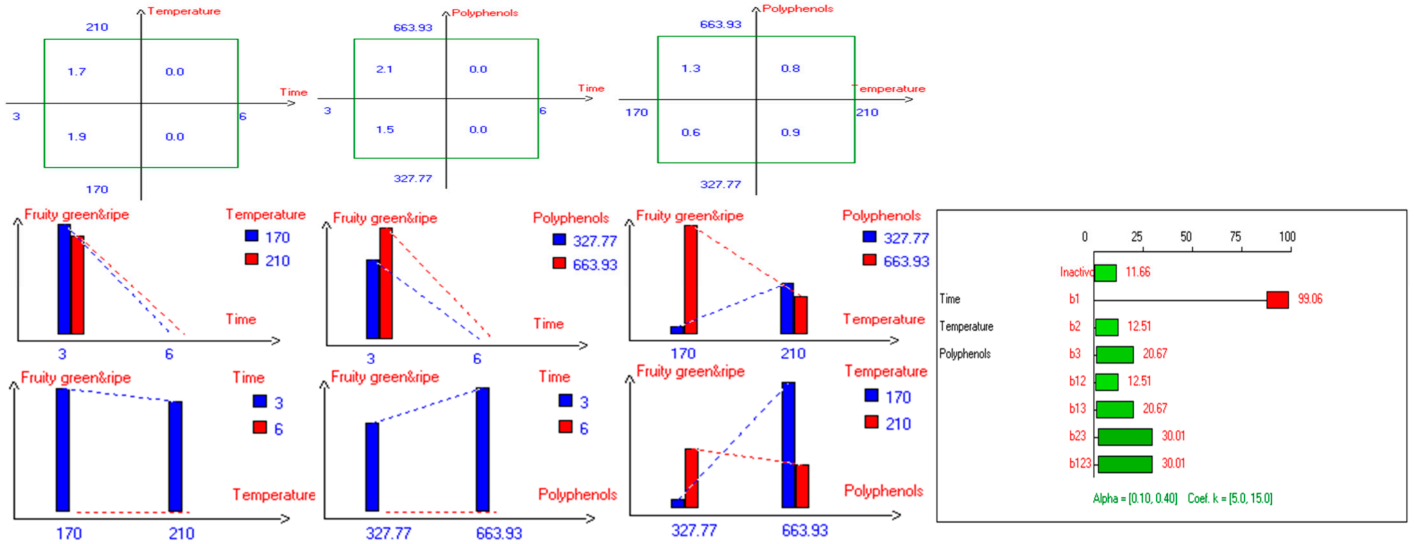

(A) (B) (C) (D)

Figure S22. Combined interactions between the independent variables on fruity in EVOO cv. Koroneiki: (**A**) x_1 and x_2, (**B**) x_1 and x_3, (**C**) x_2 and x_3, and (**D**) Results of variance analysis of regression equation model and the significance changes of each indivdual independent variable and interaction between the combined independent variables on fruity score.

where, b represents significant difference when be >b_{123}; while b represents no significant difference when be ≤b_{123}. x_1: time, x_2: temperature, x_3: polyphenols.

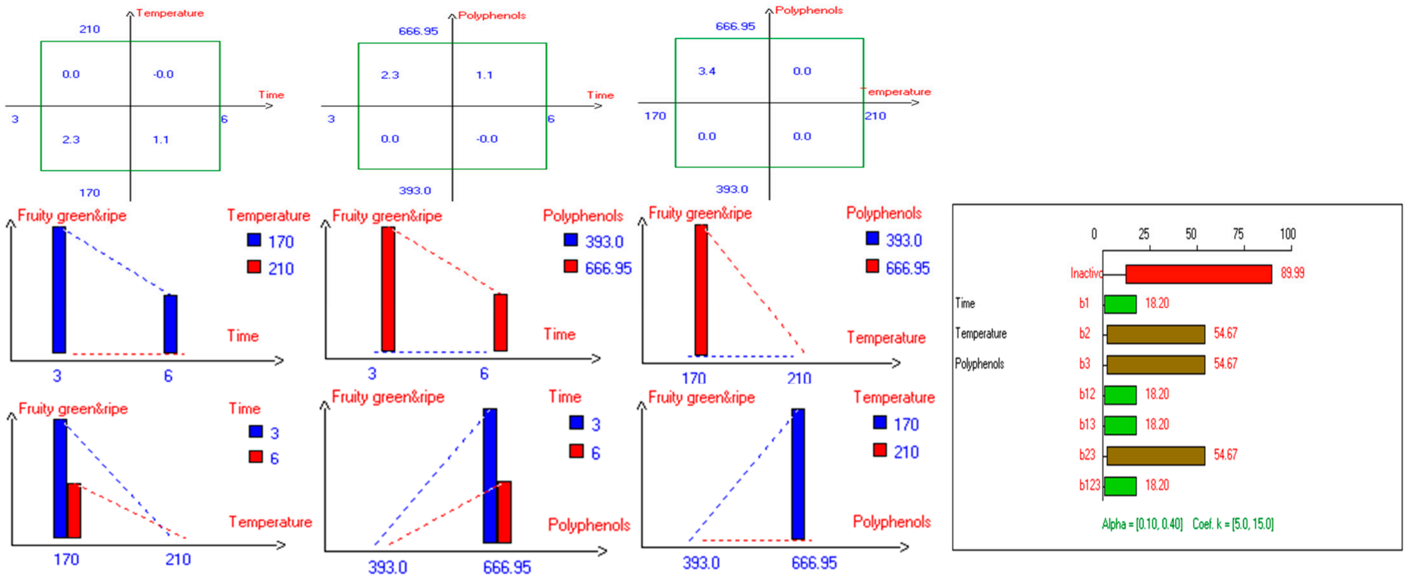

(A) **(B)** **(C)** **(D)**

Figure S23. Combined interactions between the independent variables on fruity in EVOO cv. Arbosana: (**A**) x_1 and x_2, (**B**) x_1 and x_3, (**C**) x_2 and x_3, and (**D**) Results of variance analysis of regression equation model and the significance changes of each indivdual independent variable and interaction between the combined independent variables on fruity score.

where, b represents significant difference when be >b_{123}; while b represents no significant difference when be ≤b_{123}. x_1: time, x_2: temperature, x_3: polyphenols.

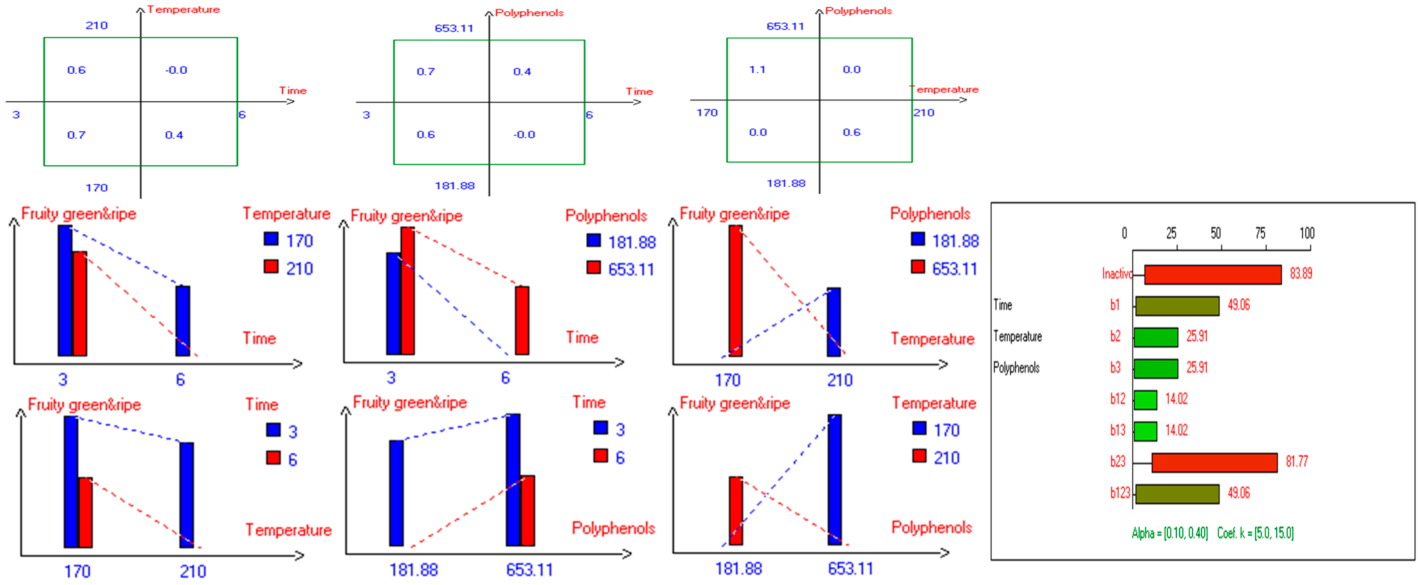

(A) **(B)** **(C)** **(D)**

Figure S24. Combined interactions between the independent variables on fruity in olive oil 1°: (**A**) x_1 and x_2, (**B**) x_1 and x_3, (**C**) x_2 and x_3, and (**D**) Results of variance analysis of regression equation model and the significance changes of each indivdual independent variable and interaction between the combined independent variables on fruity score.

where, b represents significant difference when be >b_{123}; while b represents no significant difference when be ≤b_{123}. x_1: time, x_2: temperature, x_3: polyphenols.

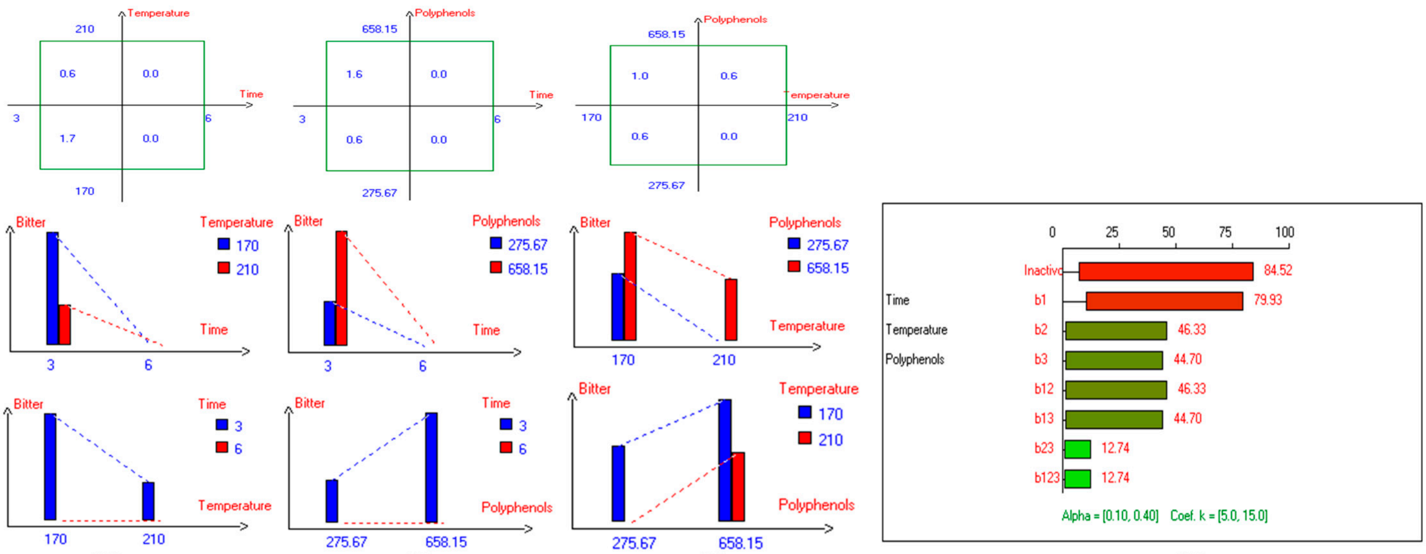

(A) (B) (C) (D)

Figure S25. Combined interactions between the independent variables on bitter in EVOO cv. Cornicabra: (**A**) x_1 and x_2, (**B**) x_1 and x_3, (**C**) x_2 and x_3, and (**D**) Results of variance analysis of regression equation model and the significance changes of each indivdual independent variable and interaction between the combined independent variables on bitter score.

where, b represents significant difference when be >b_{123}; while b represents no significant difference when be ≤b_{123}. x_1: time, x_2: temperature, x_3: polyphenols.

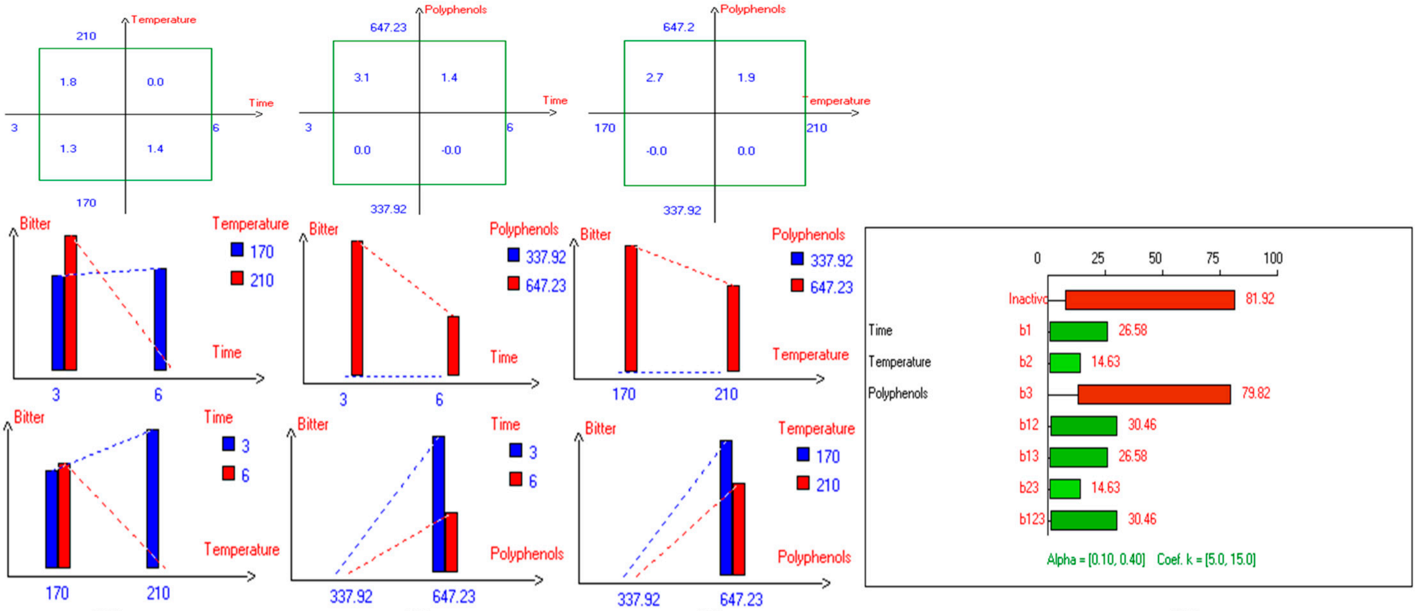

(A) (B) (C) (D)

Figure S26. Combined interactions between the independent variables on bitter in EVOO cv. Empeltre: (**A**) x_1 and x_2, (**B**) x_1 and x_3, (**C**) x_2 and x_3, and (**D**) Results of variance analysis of regression equation model and the significance changes of each indivdual independent variable and interaction between the combined independent variables on bitter score.

where, b represents significant difference when be >b_{123}; while b represents no significant difference when be ≤b_{123}. x_1: time, x_2: temperature, x_3: polyphenols.

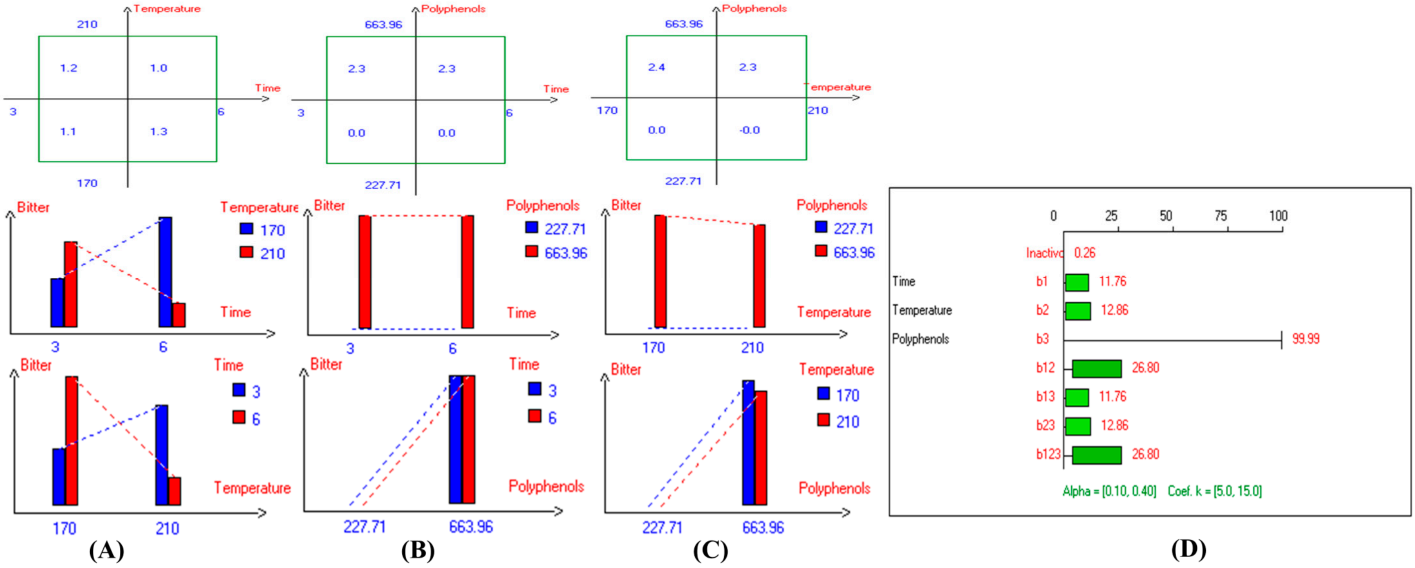

Figure S27. Combined interactions between the independent variables on bitter in EVOO cv. Arbequina: (**A**) x_1 and x_2, (**B**) x_1 and x_3, (**C**) x_2 and x_3, and (**D**) Results of variance analysis of regression equation model and the significance changes of each indivdual independent variable and interaction between the combined independent variables on bitter score.

where, b represents significant difference when be >b_{123}; while b represents no significant difference when be ≤b_{123}. x_1: time, x_2: temperature, x_3: polyphenols.

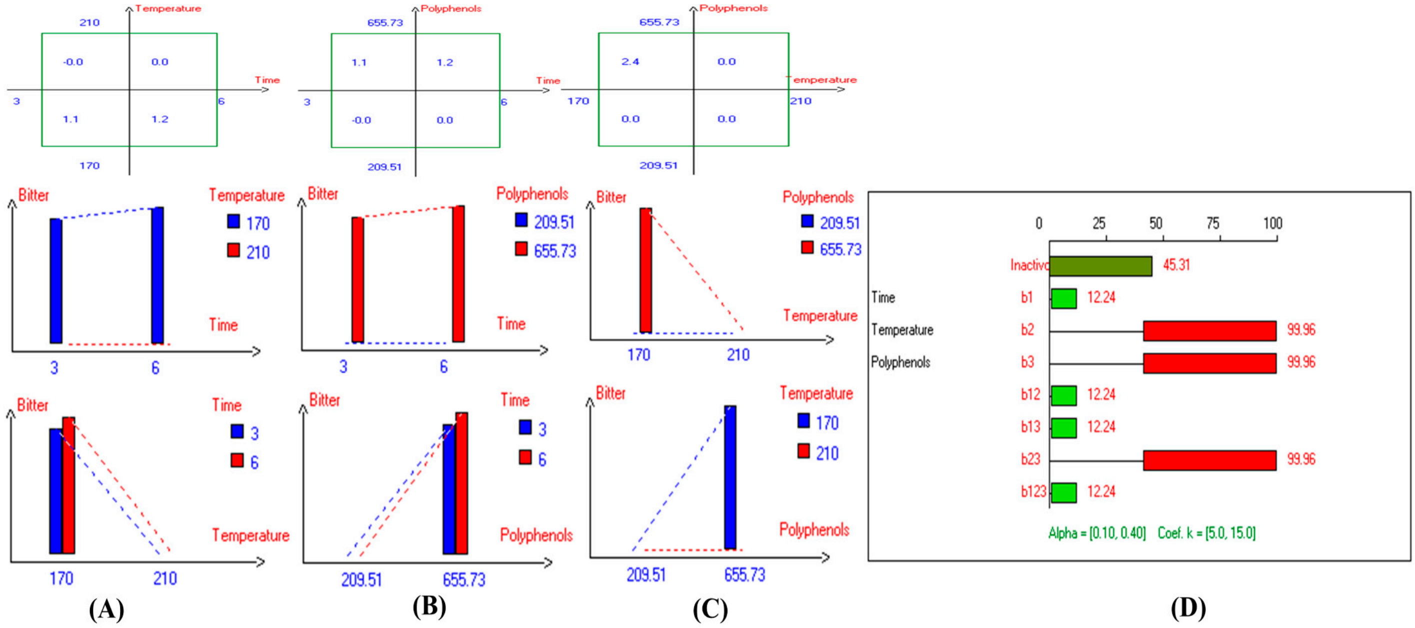

Figure S28. Combined interactions between the independent variables on bitter in EVOO cv. Hojiblanca: (**A**) x_1 and x_2, (**B**) x_1 and x_3, (**C**) x_2 and x_3, and (**D**) Results of variance analysis of regression equation model and the significance changes of each indivdual independent variable and interaction between the combined independent variables on bitter score.

where, b represents significant difference when be >b_{123}; while b represents no significant difference when be ≤b_{123}. x_1: time, x_2: temperature, x_3: polyphenols.

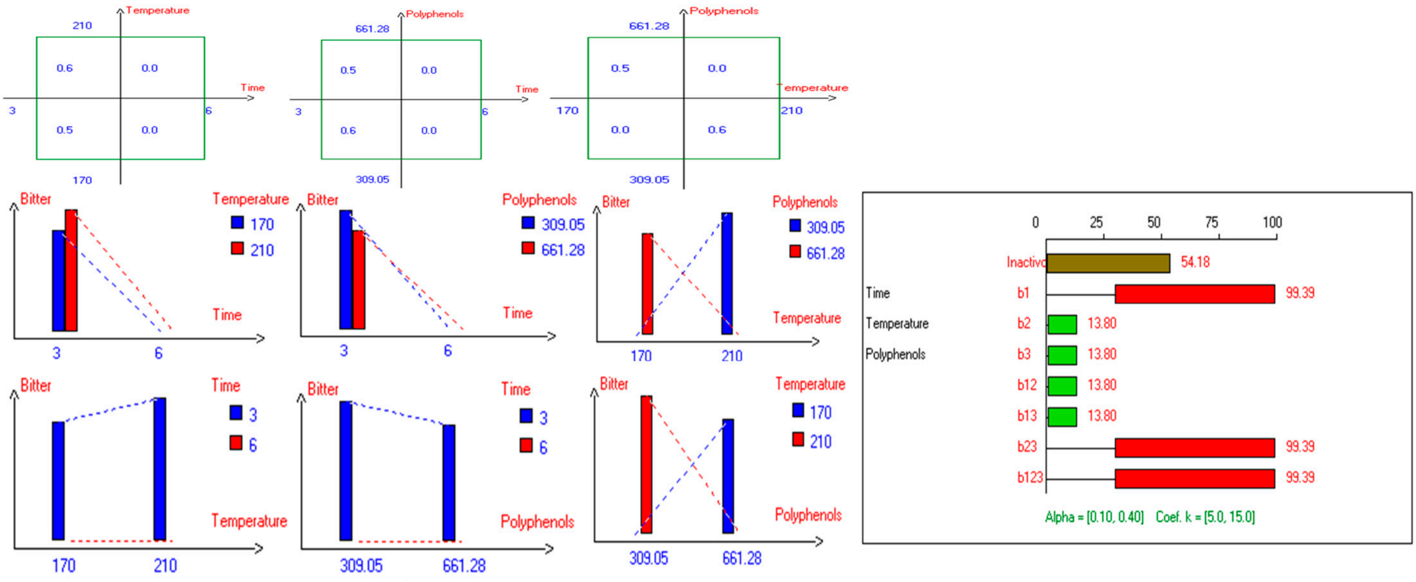

(A) (B) (C) (D)

Figure S29. Combined interactions between the independent variables on bitter in EVOO cv. Manzanilla: (**A**) x_1 and x_2, (**B**) x_1 and x_3, (**C**) x_2 and x_3, and (**D**) Results of variance analysis of regression equation model and the significance changes of each indivdual independent variable and interaction between the combined independent variables on bitter score.

where, b represents significant difference when be >b_{123}; while b represents no significant difference when be ≤b_{123}. x_1: time, x_2: temperature, x_3: polyphenols.

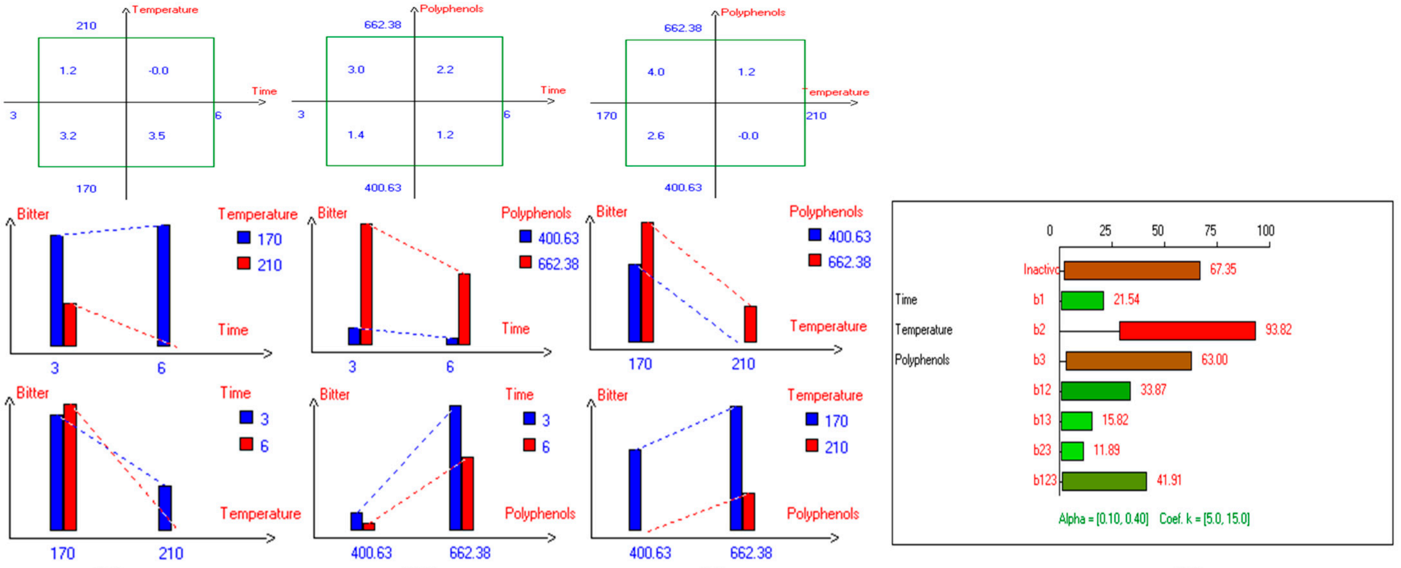

(A) (B) (C) (D)

Figure S30. Combined interactions between the independent variables on bitterness in EVOO cv. Royuela: (**A**) x_1 and x_2, (**B**) x_1 and x_3, (**C**) x_2 and x_3, and (**D**) Results of variance analysis of regression equation model and the significance changes of each indivdual independent variable and interaction between the combined independent variables on bitter score.

where, b represents significant difference when be >b_{123}; while b represents no significant difference when be ≤b_{123}. x_1: time, x_2: temperature, x_3: polyphenols.

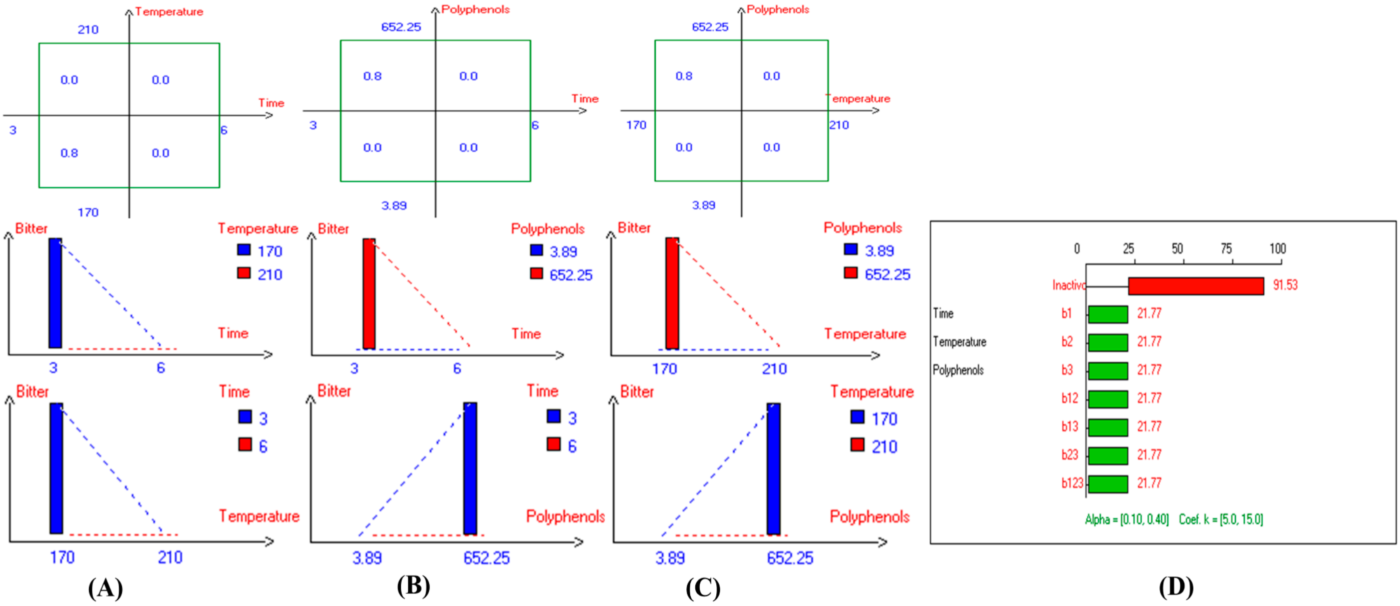

Figure S31. Combined interactions between the independent variables on bitter in Orujo oil: (**A**) x_1 and x_2, (**B**) x_1 and x_3, (**C**) x_2 and x_3, and (**D**) Results of variance analysis of regression equation model and the significance changes of each indivdual independent variable and interaction between the combined independent variables on bitter score.

where, b represents significant difference when be >b_{123}; while b represents no significant difference when be ≤b_{123}. x_1: time, x_2: temperature, x_3: polyphenols.

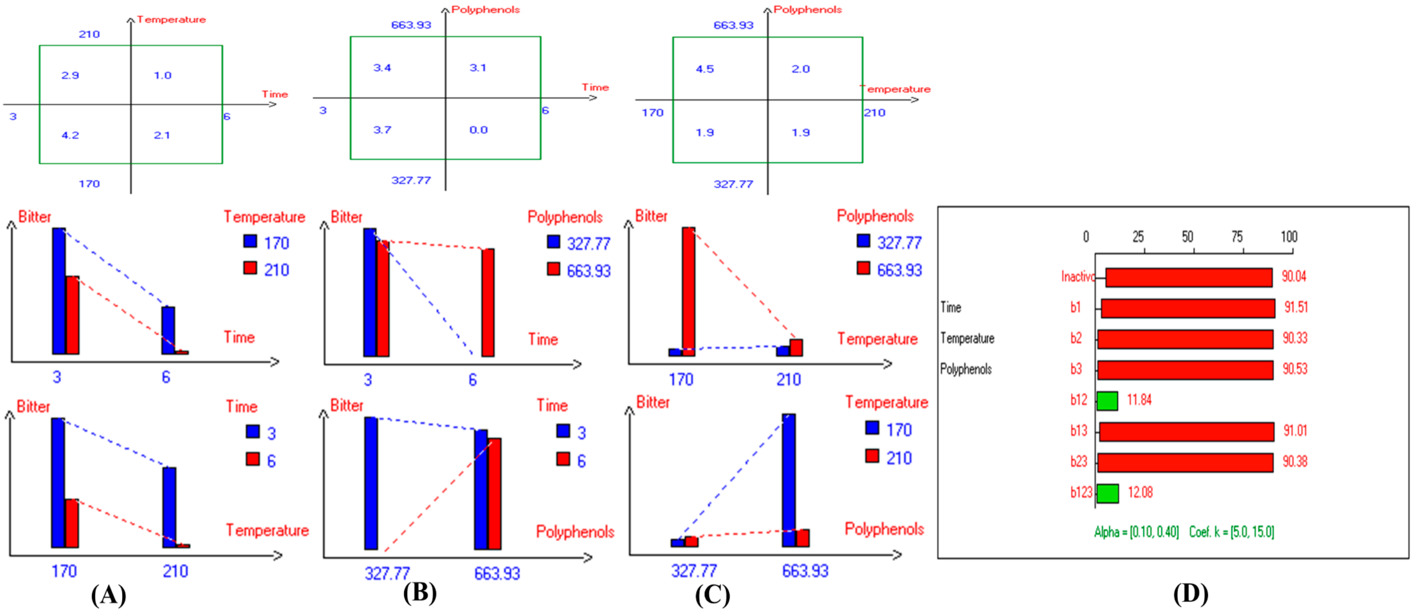

Figure S32. Combined interactions between the independent variables on bitter in EVOO cv. Koroneiki: (**A**) x_1 and x_2, (**B**) x_1 and x_3, (**C**) x_2 and x_3, and (**D**) Results of variance analysis of regression equation model and the significance changes of each indivdual independent variable and interaction between the combined independent variables on bitter score.

where, b represents significant difference when be >b_{123}; while b represents no significant difference when be ≤b_{123}. x_1: time, x_2: temperature, x_3: polyphenols.

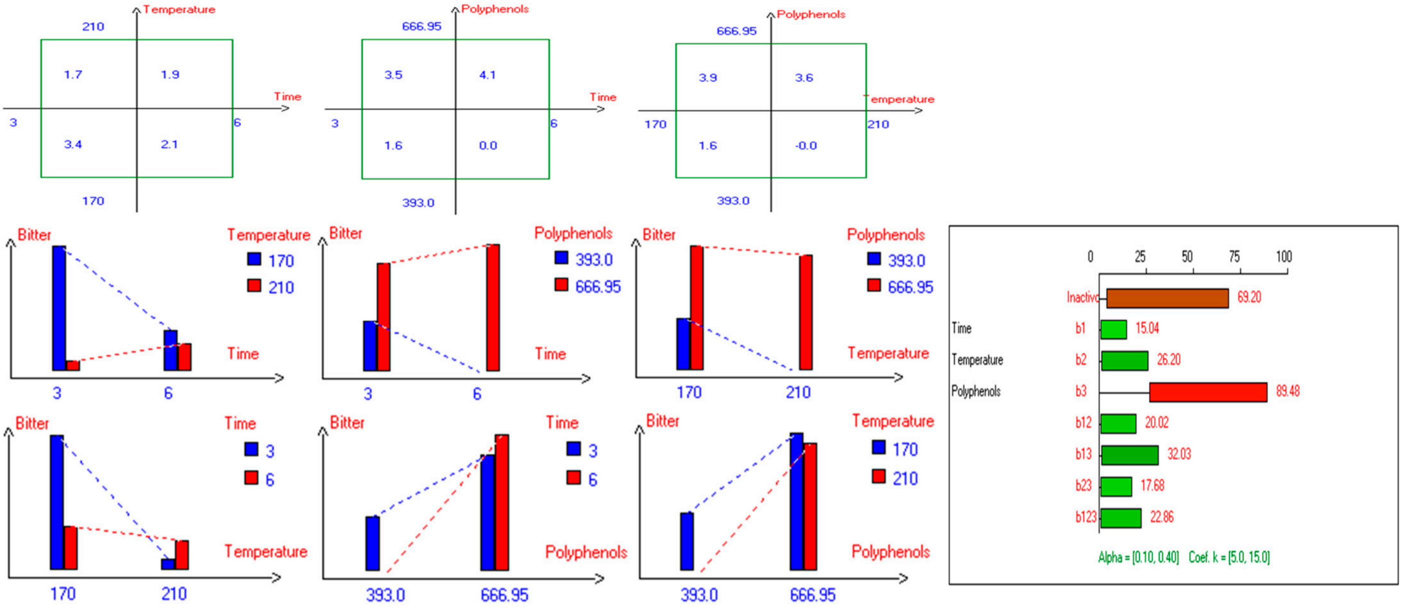

(A) (B) (C) (D)

Figure S33. Combined interactions between the independent variables on bitter in EVOO cv. Arbosana: (**A**) x_1 and x_2, (**B**) x_1 and x_3, (**C**) x_2 and x_3, and (**D**) Results of variance analysis of regression equation model and the significance changes of each indivdual independent variable and interaction between the combined independent variables on bitter score.

where, b represents significant difference when be >b_{123}; while b represents no significant difference when be ≤b_{123}. x_1: time, x_2: temperature, x_3: polyphenols.

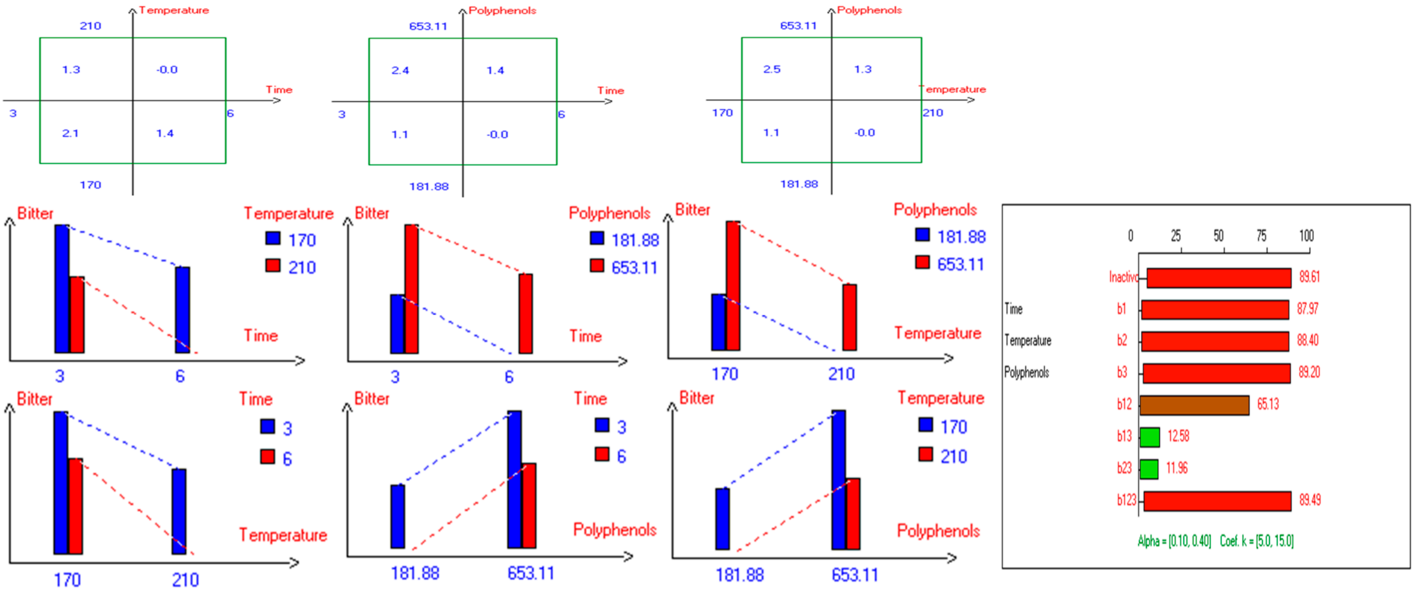

(A) (B) (C) (D)

Figure S34. Combined interactions between the independent variables on bitter in olive oil 1°: (**A**) x_1 and x_2, (**B**) x_1 and x_3, (**C**) x_2 and x_3, and (**D**) Results of variance analysis of regression equation model and the significance changes of each indivdual independent variable and interaction between the combined independent variables on bitter score.

where, b represents significant difference when be >b_{123}; while b represents no significant difference when be ≤b_{123}. x_1: time, x_2: temperature, x_3: polyphenols.

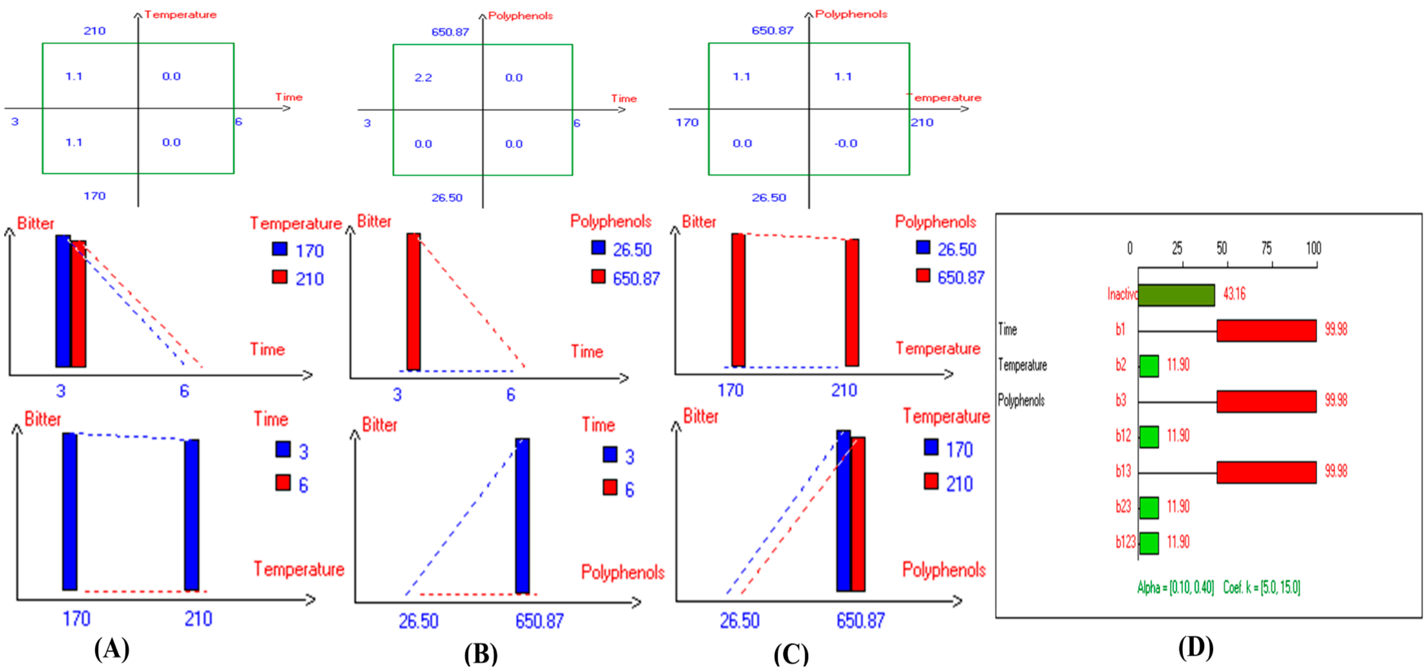

Figure S35. Combined interactions between the independent variables on bitter in olive oil 0.4°: (**A**) x_1 and x_2, (**B**) x_1 and x_3, (**C**) x_2 and x_3, and (**D**) Results of variance analysis of regression equation model and the significance changes of each indivdual independent variable and interaction between the combined independent variables on bitter score.

where, b represents significant difference when be $>b_{123}$; while b represents no significant difference when be $\leq b_{123}$. x_1: time, x_2: temperature, x_3: polyphenols.

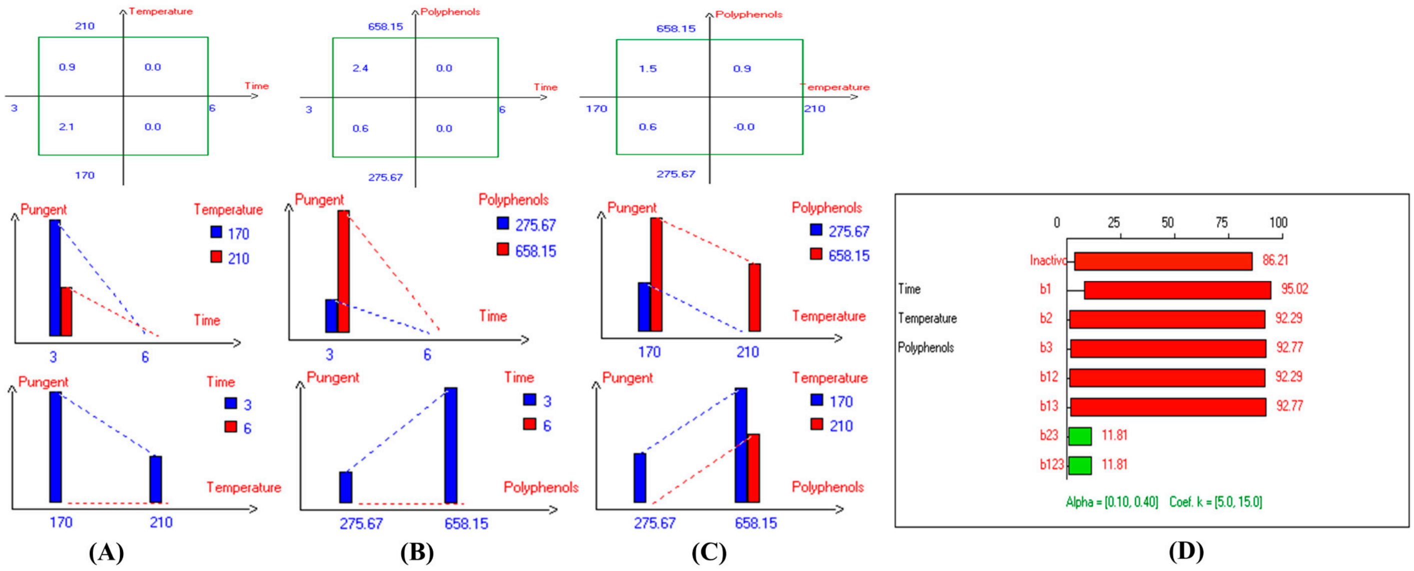

Figure S36. Combined interactions between the independent variables on pungent in EVOO cv. Cornicabra: (**A**) x_1 and x_2, (**B**) x_1 and x_3, (**C**) x_2 and x_3, and (**D**) Results of variance analysis of regression equation model and the significance changes of each indivdual independent variable and interaction between the combined independent variables on pungent score.

where, b represents significant difference when be $>b_{123}$; while b represents no significant difference when be $\leq b_{123}$. x_1: time, x_2: temperature, x_3: polyphenols.

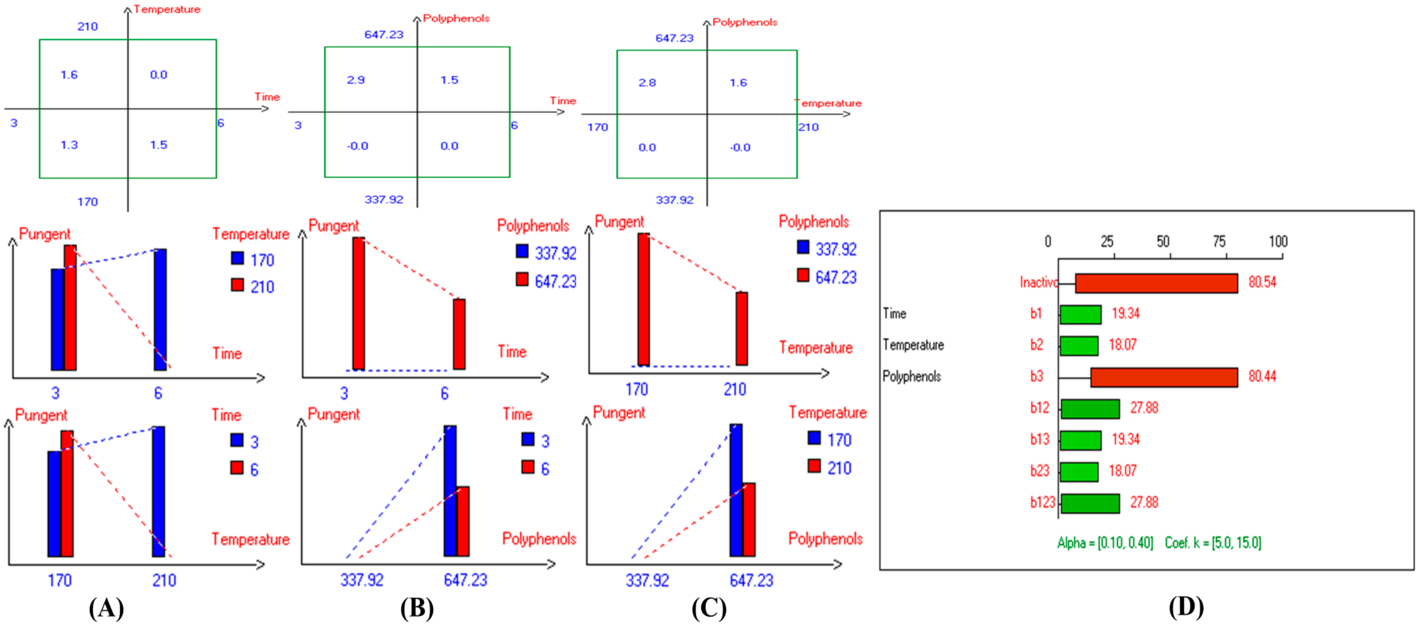

Figure S37. Combined interactions between the independent variables on pungent in EVOO cv. Empeltre: (**A**) x_1 and x_2, (**B**) x_1 and x_3, (**C**) x_2 and x_3, and (**D**) Results of variance analysis of regression equation model and the significance changes of each indivdual independent variable and interaction between the combined independent variables on pungent score.

where, b represents significant difference when be >b_{123}; while b represents no significant difference when be ≤b_{123}. x_1: time, x_2: temperature, x_3: polyphenols.

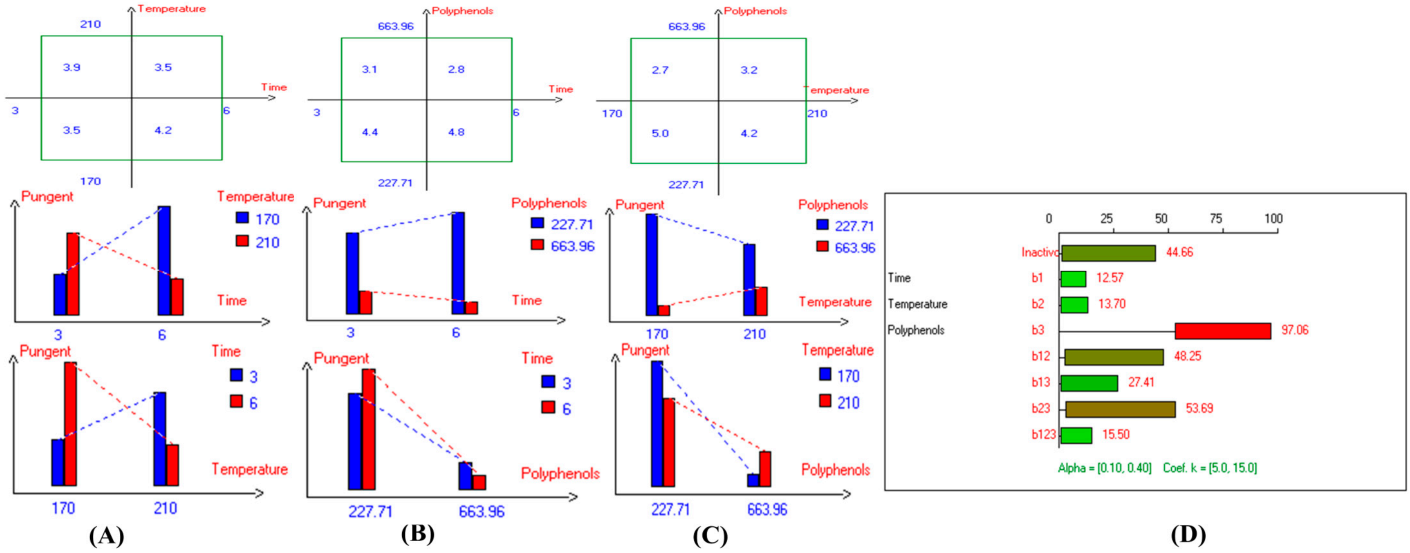

Figure S38. Combined interactions between the independent variables on pungent in EVOO cv. Arbequina: (**A**) x_1 and x_2, (**B**) x_1 and x_3, (**C**) x_2 and x_3, and (**D**) Results of variance analysis of regression equation model and the significance changes of each indivdual independent variable and interaction between the combined independent variables on pungent score.

where, b represents significant difference when be >b_{123}; while b represents no significant difference when be ≤b_{123}. x_1: time, x_2: temperature, x_3: polyphenols.

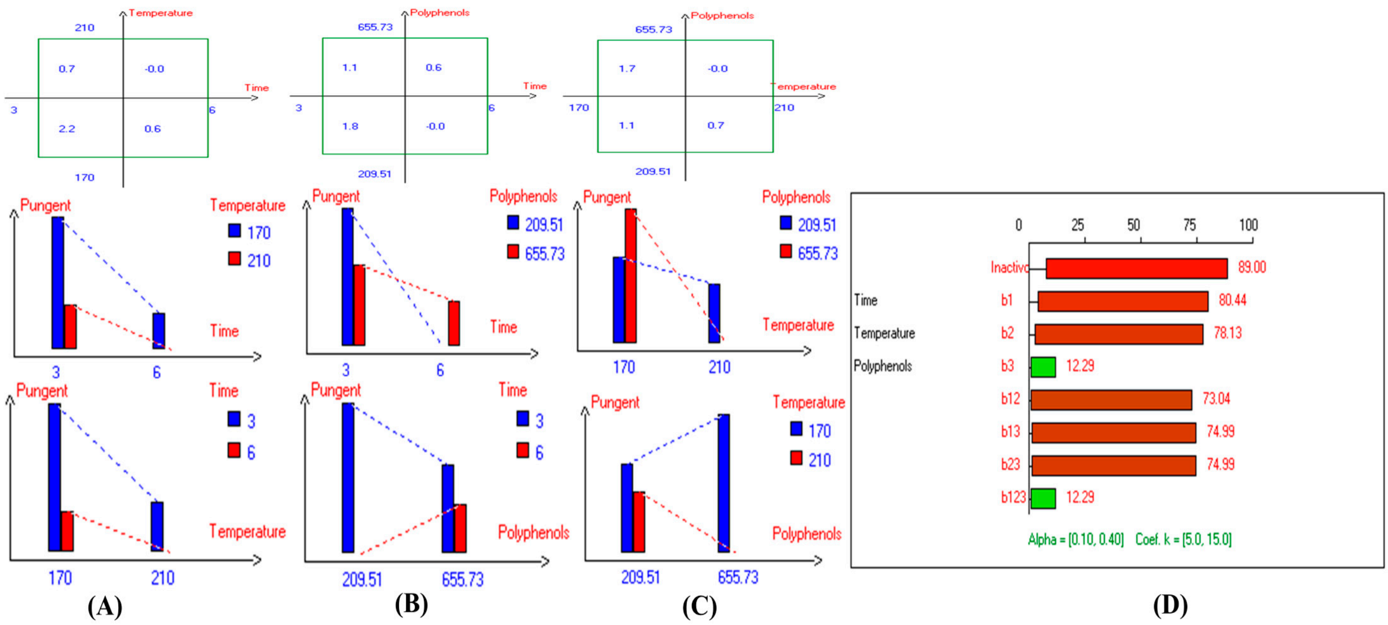

Figure S39. Combined interactions between the independent variables on pungent in EVOO cv. Hojiblanca: (**A**) x_1 and x_2, (**B**) x_1 and x_3, (**C**) x_2 and x_3, and (**D**) Results of variance analysis of regression equation model and the significance changes of each indivdual independent variable and interaction between the combined independent variables on pungent score.

where, b represents significant difference when be $>b_{123}$; while b represents no significant difference when be $\leq b_{123}$. x_1: time, x_2: temperature, x_3: polyphenols.

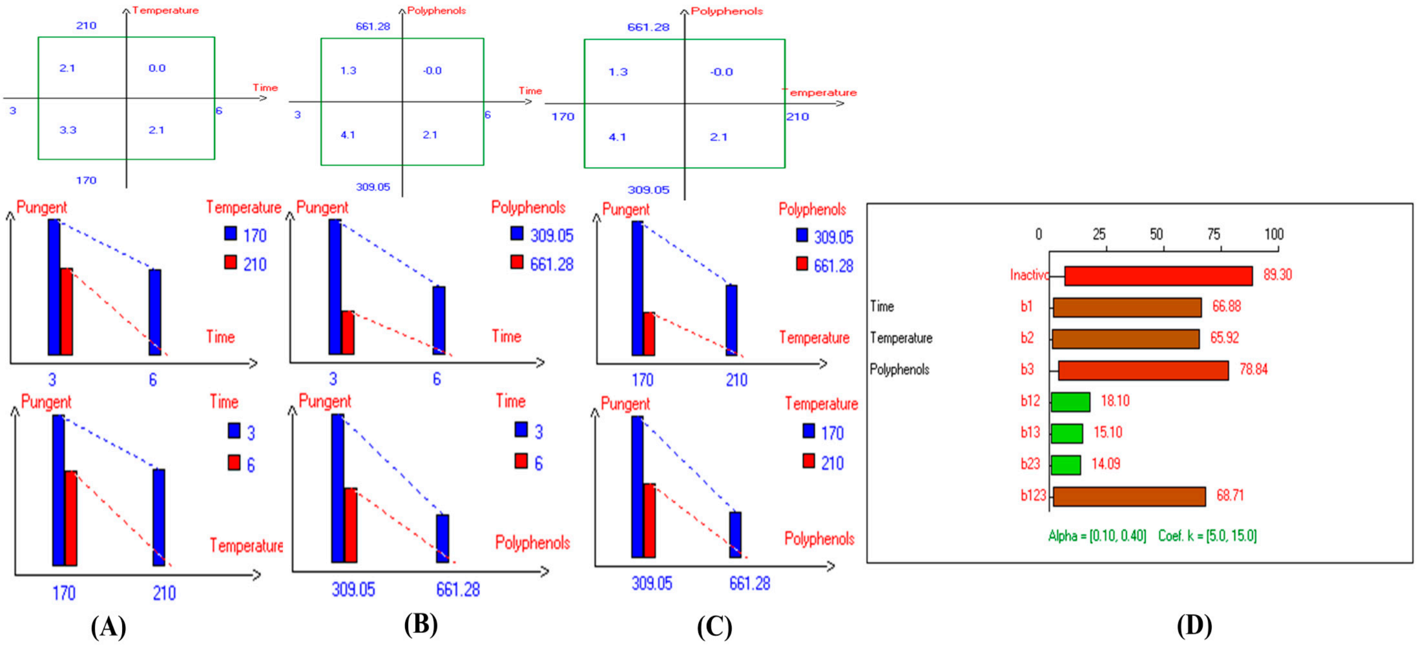

Figure S40. Combined interactions between the independent variables on pungent in EVOO cv. Manzanilla: (**A**) x_1 and x_2, (**B**) x_1 and x_3, (**C**) x_2 and x_3, and (**D**) Results of variance analysis of regression equation model and the significance changes of each indivdual independent variable and interaction between the combined independent variables on pungent score.

where, b represents significant difference when be $>b_{123}$; while b represents no significant difference when be $\leq b_{123}$. x_1: time, x_2: temperature, x_3: polyphenols.

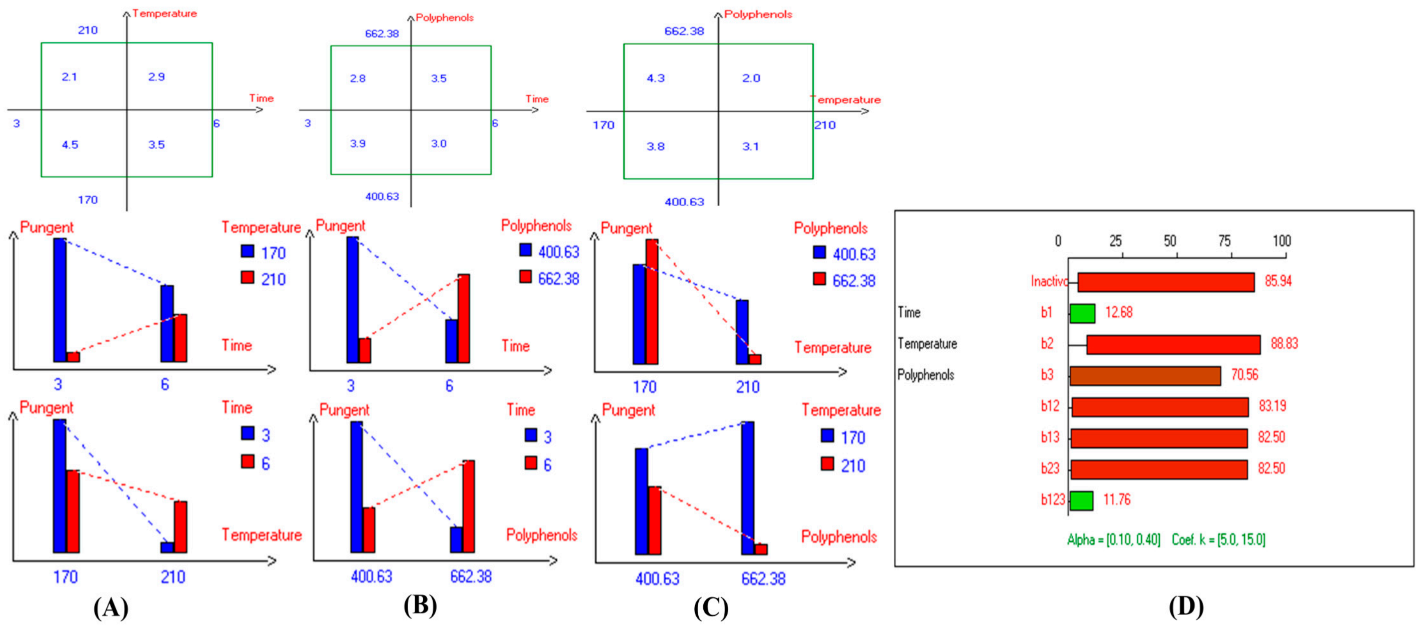

Figure S41. Combined interactions between the independent variables on pungent in EVOO cv. Royuela: (**A**) x_1 and x_2, (**B**) x_1 and x_3, (**C**) x_2 and x_3, and (**D**) Results of variance analysis of regression equation model and the significance changes of each indivdual independent variable and interaction between the combined independent variables on pungent score.

where, b represents significant difference when be $>b_{123}$; while b represents no significant difference when be $\leq b_{123}$. x_1: time, x_2: temperature, x_3: polyphenols.

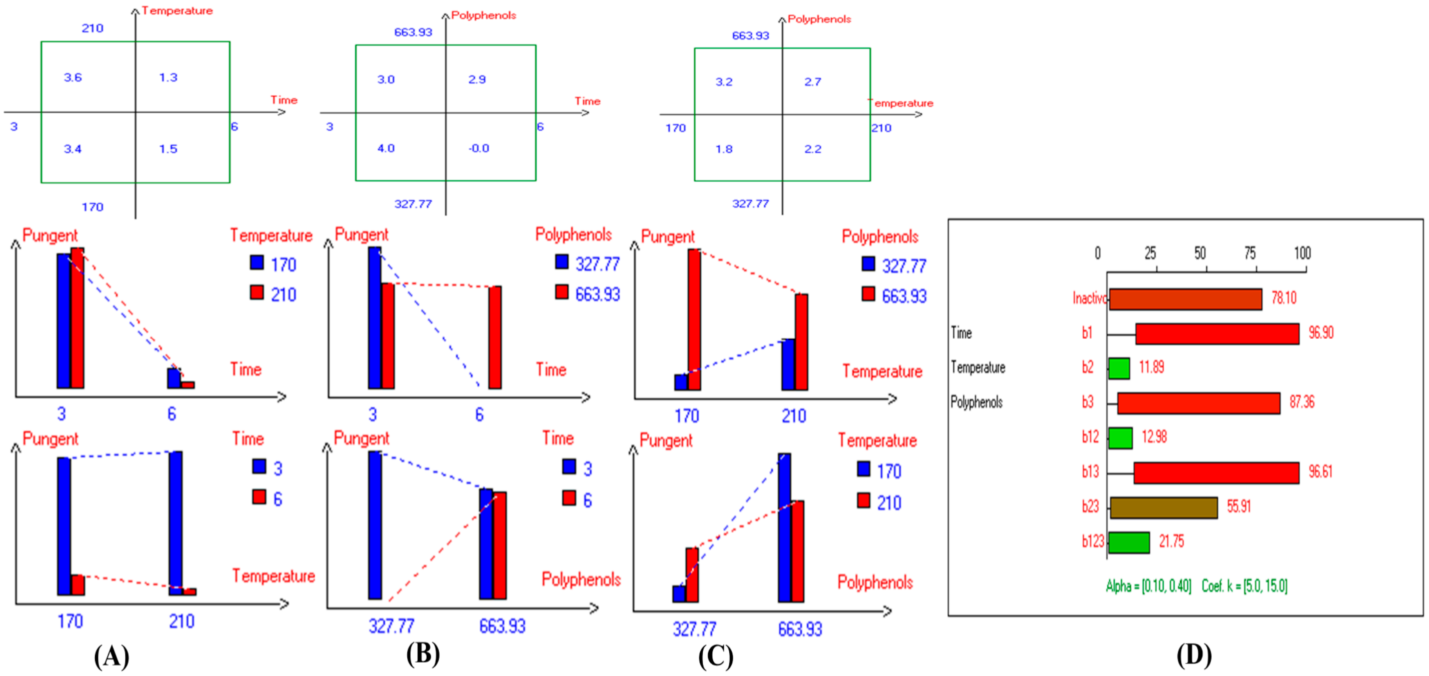

Figure S42. Combined interactions between the independent variables on pungent in EVOO cv. Koroneiki: (**A**) x_1 and x_2, (**B**) x_1 and x_3, (**C**) x_2 and x_3, and (**D**) Results of variance analysis of regression equation model and the significance changes of each indivdual independent variable and interaction between the combined independent variables on pungent score.

where, b represents significant difference when be $>b_{123}$; while b represents no significant difference when be $\leq b_{123}$. x_1: time, x_2: temperature, x_3: polyphenols.

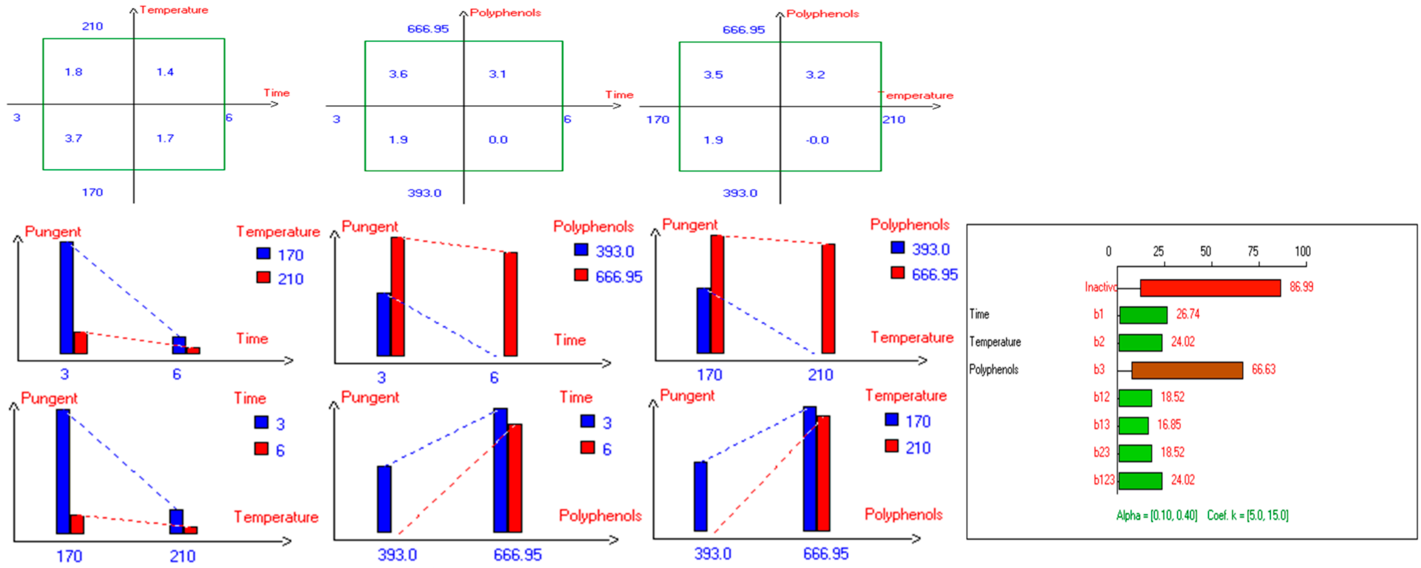

(A) (B) (C) (D)

Figure S43. Combined interactions between the independent variables on pungent in EVOO cv. Arbosana: (**A**) x_1 and x_2, (**B**) x_1 and x_3, (**C**) x_2 and x_3, and (**D**) Results of variance analysis of regression equation model and the significance changes of each indivdual independent variable and interaction between the combined independent variables on pungent score.

where, b represents significant difference when be >b_{123}; while b represents no significant difference when be ≤b_{123}. x_1: time, x_2: temperature, x_3: polyphenols.

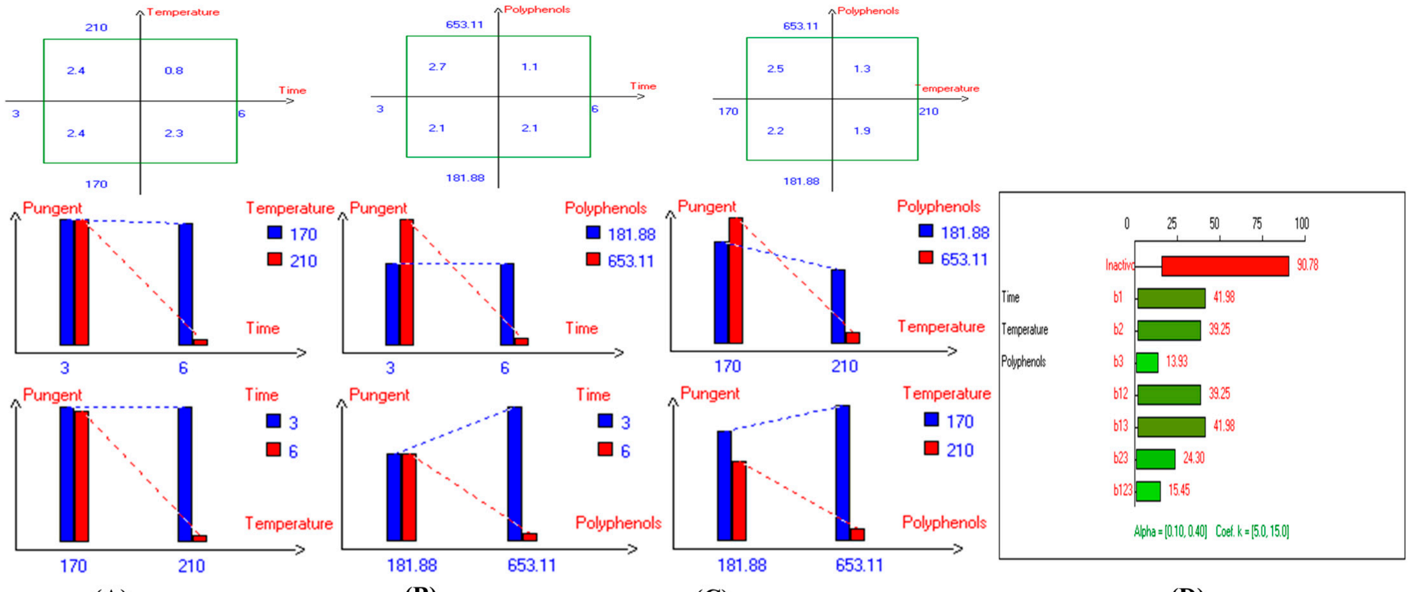

(A) (B) (C) (D)

Figure S44. Combined interactions between the independent variables on pungent in olive oil 1°: (**A**) x_1 and x_2, (**B**) x_1 and x_3, (**C**) x_2 and x_3, and (**D**) Results of variance analysis of regression equation model and the significance changes of each indivdual independent variable and interaction between the combined independent variables on pungent score.

where, b represents significant difference when be >b_{123}; while b represents no significant difference when be ≤b_{123}. x_1: time, x_2: temperature, x_3: polyphenols.
